# Anion Binding and Aggregation of *N*‑Terminal α‑Synuclein Peptides

**DOI:** 10.1021/acsomega.5c02618

**Published:** 2025-05-21

**Authors:** Ruiqing Wang, Busayo D. Alagbe, Henry S. Ashbaugh, Bruce C. Gibb

**Affiliations:** † Department of Chemistry, 5783 Tulane University, New Orleans, Louisiana 70118, United States; ‡ Department of Chemical and Biomolecular Engineering, Tulane University, New Orleans, Louisiana 70118, United States

## Abstract

α-Synuclein (α-Syn) is linked to the pathogenesis of Parkinson’s disease by its misfolding, aggregation, and accumulation in Lewy bodies, the characteristic amyloids of Parkinson’s. *N-*terminal binding to phospholipid membranes and the resulting random-coil to helical transition are key to the aggregation of α-Syn. However, despite the recognized affinity for the *N*-terminal domain for phospholipids, the anion affinity for this region has not been comprehensively examined. To probe the effects of monovalent anion binding to the *N*-terminus, we report here on studies with the 15-mer *N*-terminal peptide of α-Syn and two mutants in which all three lysines of the wild-type sequence are replaced with either arginine or histidine (_1_MDVFM **X** GLS **X** A **X** EGV_15_; **X** = K, R, or H). Our studies reveal that charge-diffuse anions have a measurable affinity, binding weakly to the midsection of the sequences. However, binding does not induce significant long-range ordering. Nevertheless, MD simulations do reveal a compaction of the peptides in the presence of ClO_4_^–^, supporting the conclusion that anion binding screens the positively charged residues, reducing the effective net positive charge of the peptide and inducing aggregation. Aggregation studies revealed that this reverse Hofmeister effect correlates with anion affinity and that at intermediate salt concentrations or low pH, aggregation follows the Finke–Watzky model. Our findings suggest that changes in simple salt concentrations are unlikely to affect the structure of the *N*-terminal region of α-Syn and highlight that multipoint interactions between polyanionic phospholipid membranes are a necessary requirement for the random-coil to helical transition observed in the wild type.

## Graphical abstract

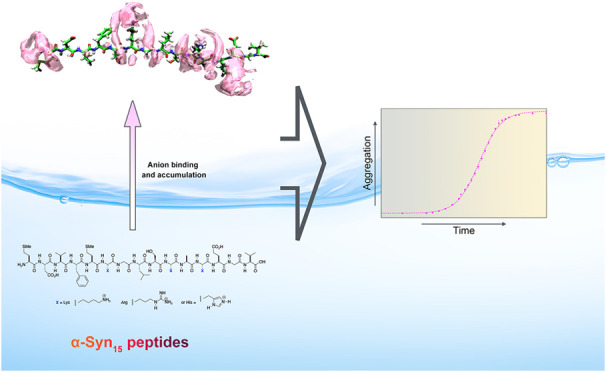

## Introduction

α-Synuclein (α-Syn) is an intrinsically disordered protein (IDP)^5–8^ linked to the pathogenesis of Parkinson’s disease by its misfolding, aggregation, and accumulation in Lewy bodies,^9,10^ the characteristic amyloids of Parkinson’s. The *N-*terminal region of α-Syn (residues 1–60) is regarded as a key factor in aggregation and fibril formation,^1–4^ and phospholipid binding of the terminus is associated with both fibril formation^9–13^ and the triggering of aggregation and neuronal death.^14,15^

Exogenous factors can frequently induce secondary structure formation in IDPs,^16–18^ and α-Syn is itself predisposed^19^ under certain conditions to form α-helix-rich tetramers and related oligomers.^11,20,21^ Thus, phospholipid membrane binding of the *N-*terminal region triggers a random-coil to helical transition down the length of the protein.^22–28^ More generally, the effects of buffers and salts on the α-Syn structure have been investigated.^29,30^ For example, the *N*-terminus—specifically the _1_MDVFMKGLS_9_ and _48_VAHGV_52_ regions^30^—has a high affinity for Cu­(II); binding that induces structural changes in the protein. Copper also binds weakly to the *C*-terminal region of α-Syn, as do other divalent metal ions.^29^ However, despite the recognized affinity for the *N*-terminal domain to bind to phospholipid membranes, anion affinity to α-Syn has not been comprehensively examined.^31^

Here, we report on the affinity and effects of binding monovalent anions to the 15-mer *N*-terminal peptide of α-Syn and two mutants in which all three lysines of the wild-type sequence are replaced with either arginine or histidine (_1_MDVFM **X** GLS **X** A **X** EGV_15_; **X** = K, R, or H). With these three peptides, we wished to determine if simple monovalent anions, from charge-dense Cl^–^ to charge-diffuse ReO_4_^–^, were capable of inducing observable structural changes and/or whether these had an effect on the aggregation propensity of the peptides. We opted for 15-mer sequences to maximize the likelihood of anion binding by including three positive charges and additionally examined the two triple mutants as the anion binding properties of Lys, Arg, and His are known to be very different.^32–35^ Our results demonstrate that although monovalent anion affinity could be qualified and in select cases quantified, none of the anions investigated were capable of inducing a discernible secondary structure in the peptides. Nevertheless, anion binding did affect the aggregation kinetics of the peptides in a manner that followed the reverse Hofmeister effect.^31,35–41^

## Results and Discussion

Peptides **1**–**3** are shown in Figure 1. A sequence of 2D NMR spectroscopy experiments, namely, total correlation spectroscopy (TOCSY), rotating-frame Overhauser effect spectroscopy (ROESY), correlation spectroscopy (COSY), and ^1^H–^15^N heteronuclear single quantum correlation (HSQC), was employed to characterize each (see the Supporting Information (SI), Section 2). All peptide solutions were 5 mM, utilizing either 50 mM phosphate buffer (pH = 2.3) or 50 mM sodium acetate (pH = 5.2) in 9:1 H_2_O/D_2_O.

**Figure 1:**
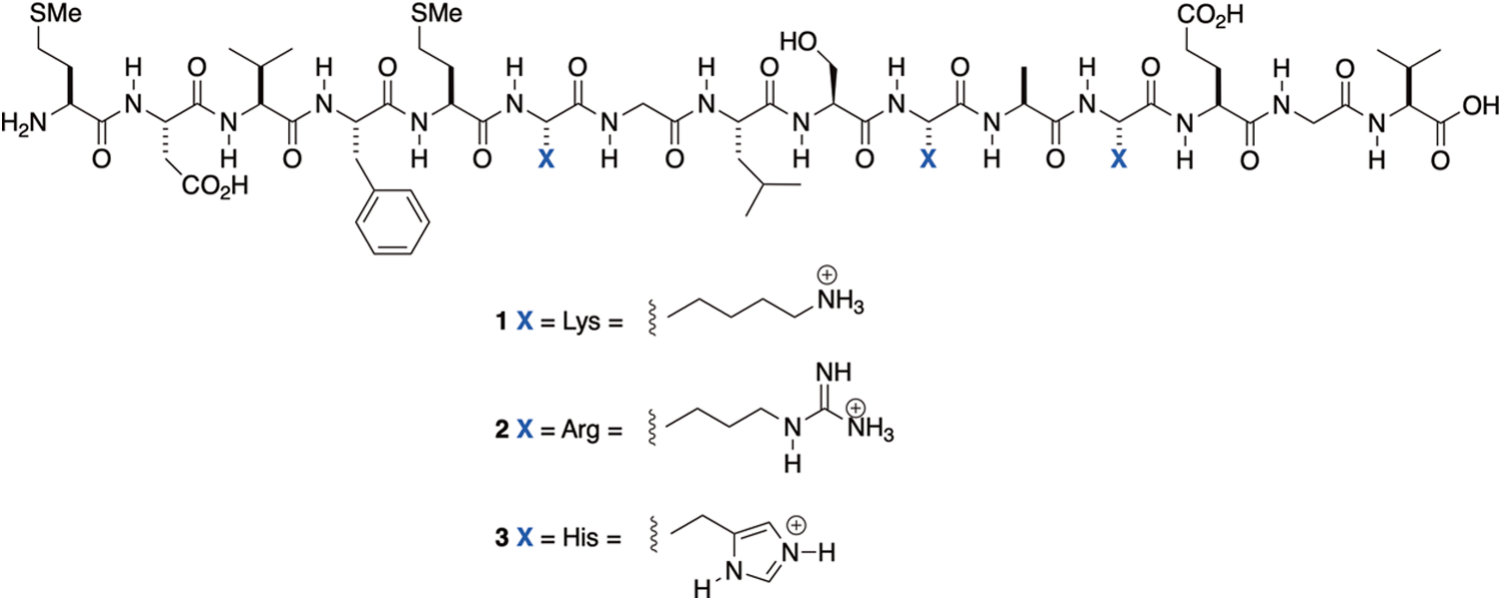
Peptides **1**–**3**.

CD spectroscopy (SI, Section 5) demonstrated that, as anticipated, the three peptides possessed random-coil structures; each peptide possessed a negative peak around 200 nm. To confirm this, we used TROSY NMR^42,43^ to measure the coupling constant between each amide N–H and C_α_H of the same residue (^3^ *J*_HN–Hα_) and hence estimate the corresponding phi (φ) dihedral angle. We focused on peptide **3** because it offered excellent signal anisotropy (SI, Section 3.4), revealing *J* values between 5.67 and 8.23 Hz. Residues G7, A11, and G14 were clustered between 5.67 and 6.30 Hz—indicating an essentially random-coil structure—whilst the other residues were clustered between 6.65 and 8.23, reflecting that these residues sampled more extended or helical regions of the Ramachandran space.^42,44^ Indeed, the three highest coupling constants were all β-branched residues (V3, L8, and V15) that possess a higher propensity for the extended or sheet structure. We also probed for structure development using VT NMR (SI, Section 3.3). This revealed that peptides **1**–**3** maintained their largely random-coil structure at lower temperatures. For example, with peptide **1**, the temperature dependence of each amide N–H signal was linear between 5 and 35 °C and displayed a narrow range of temperature coefficients, from 2.14 to 4.99 ppb/K. In other words, NMR demonstrated that each peptide possessed a random structure and that this remained the case at lower temperatures.

To probe the binding of anions to peptides **1**–**3**, we first turned to ^1^H NMR chemical shifts to map the association of four anions: Cl^–^, Br^–^, I^–^, and ClO_4_^–^. As an example, Figure 2a shows the chemical shifts of each amide proton of recipient^45^ **1** in response to the addition of 93 equiv of the corresponding sodium salt. The area of each “bubble” on or adjacent to each amide N–H group is proportional to the signal shift between the free and bound states (Δδ = (δ_N–H_ values for **1** + salt) – (δ_N–H_ values for **1**)), with the largest shift (0.136 ppm) being observed for residue K12 upon the addition of NaClO_4_.

**Figure 2:**
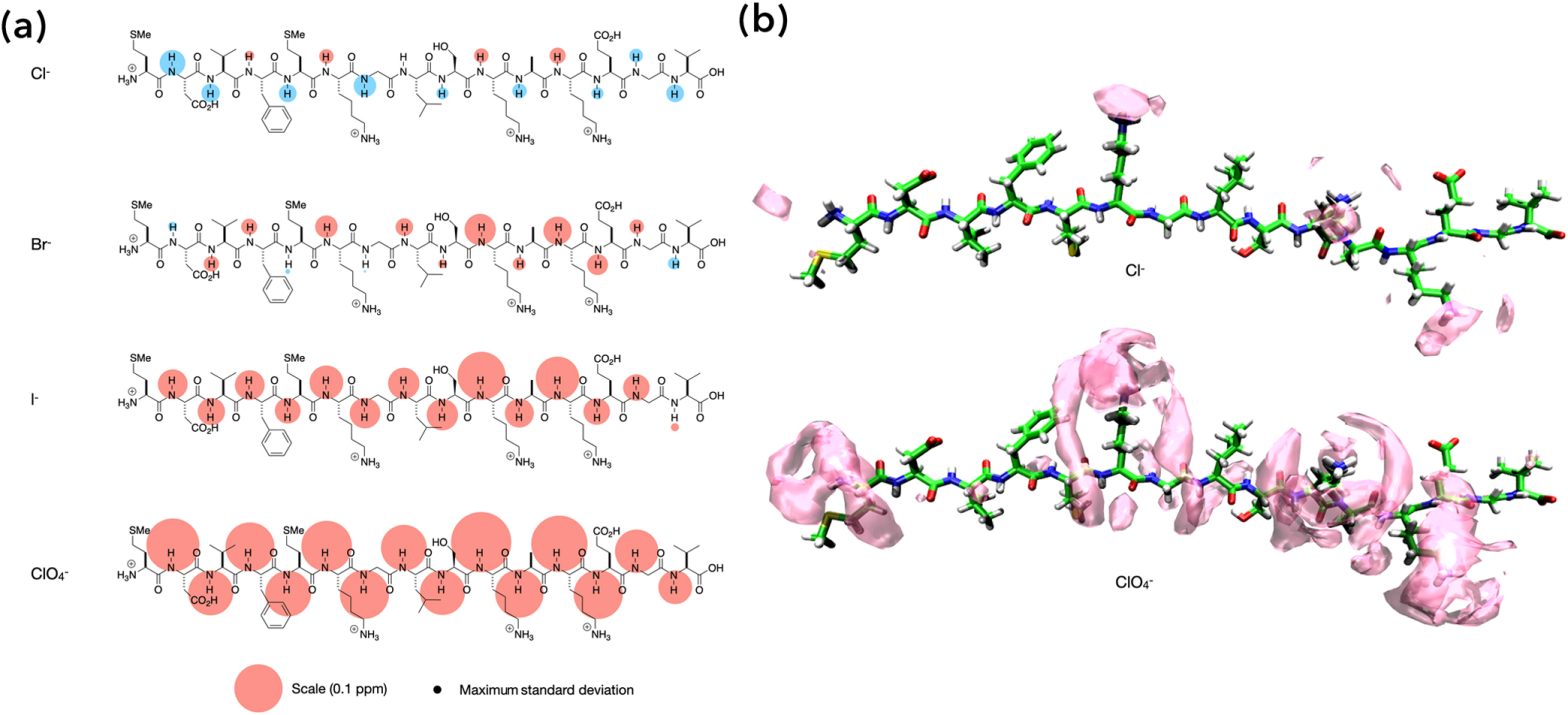
(a) Bubble maps showing the unreferenced signal shifts of the main-chain amide groups of recipient **1** for the addition of 93 equiv of NaCl, NaBr, NaI, and NaClO_4_ at pH = 2.3. In each map, the effect of adding an anion to the recipient to form a complex is defined by Δδ = (δ_N–H_ values for **1** + salt) – (δ_N–H_ values for **1**), with the area of each bubble proportional to the observed Δδ value from the ^1^H NMR spectra. Upfield signals are shown in red, and downfield shifts are shown in blue. Where shifts are small, the circle is shown above or below the amide H atom. A scale and error bubble (±0.005 ppm) is shown at the foot of the figure. (b) Spatial distribution functions revealing the association of Cl^–^ and ClO_4_^–^ to recipient **1** in an extended conformation obtained from MD simulations. All anionic “clouds” (magenta) correspond to probability thresholds set to 8 × bulk density.

It is well understood that charge-dense anions such as Cl^–^ preferentially form hydrogen bonds with amide N–H groups,^46–48^ whilst charge-diffuse anions such as ClO_4_^–^ associate with nonpolar moieties.^35,38,41,49–52^ Nevertheless, we have previously found that amide ^1^H NMR shifts are good proxies for mapping general anion binding in simple peptides.^34^ Small downfield shifts, such as those of the majority of amide groups of **1** upon the addition of NaCl, are attributed to magnetic susceptibility and other non-specific ionic strength effects.^34^ In contrast, anion association is characterized by upfield shifts. In the case of Cl^–^, any upfield shifts are small and only observed at F4, K6, K10, and K12. This reveals that the small degree of association with recipient **1** is primarily controlled by Coulombic interactions, though it may be the case that the relatively poor solvation of F4 combines with the electrostatic field of K6 to lead to anion binding and an upfield shift of F4. This noted, the NMR shift data did not provide any evidence of Cl^–^ binding at the positively charged *N*-terminus.

As anticipated, Br^–^ binding was more evident, with most of the N–H amide groups of **1** undergoing upfield shifts. The exceptions were E2, M5, and V15. These data perhaps best demonstrate that anion association is greatest when the anion can make multiple contacts with the peptide recipient, i.e., in its midsection. In contrast, binding to the termini is not evident. This preference for binding to the midsection is supported by prior work probing the binding of anions to poly­(ethylene oxide)­s.^53^ Moreover, as was the case with Cl^–^ binding, association is the strongest, proximal to the positively charged residues. Interestingly, this data also shows that the signal shift of N–H on the *N*–terminal side of each positively charged side chain (N–H_*i*_) is larger than that for N–H on the *C*–terminal side (N–H_*i*+1_). This may simply be a reflection of the two-bond separation between N–H_*i*_ and C_α,*i*_ (possessing the charged side chain) versus the three-bond separation between C_α,*i*_ and N–H_*i*+1_. However, we surmise that the partial negative charge on C=O_*i*_—situated between the charged group and N–H_*i*+1_—also disfavors anion association toward the *C*-terminal side of a charged side chain.

The chemical shift data suggest that I^–^ and ClO_4_^–^ have much higher affinity, with more substantial upfield shifts reported by all amide signals. Although the subtle observations obtained from Cl^–^ and Br^–^ association are somewhat lost with the higher affinity anions, in both cases, the larger upfield shifts in K10 and K12 and the smallest shift in V15 were still apparent.

We were not able to assess anion binding to recipient **2**, nor binding to **3** at pH = 5.2. However, for **1** at both pH values and **3** at pH = 2.3, all of the above trends held (SI, Section 3). Thus, the more charge-diffuse anions bound more strongly, with the focus on the affinity in the midsection of the peptide adjacent to positively charged residues. Additionally, from the thirty-six examples available, there was only one exception (K6 of **1** binding I^–^ at pH 5.2) where anion association proximal to a positively charged residue (*i*) wasn’t stronger at N–H_*i*_ than at N–H_*i*+1_. Thus, it seems to be a general rule that the combination of the extra bond separation between N–H_*i*+1_ and the charge, and intervening C=O_*i*_, results in a stronger association of the anion to the *N*–H_*i*_ charged residue in random coils.

An alternative visualization of anion binding was obtained from explicit water model MD simulations. These were performed in bulk water (using TIP4P2005 waters^54^) at 25 °C and 1 bar, with the peptides modeled using the Amber-03ws all-atom force field,^55–57^ the ions modeled using the generalized Amber force field (GAFF),^58^ and their partial charges obtained from AM1-BCC calculations.^59^ Each simulation included one peptide in the 1+ state (with a chloride counter ion for overall neutrality to represent their approximate protonation state at pH = 5.2) and if added—thirty-three equivalents (260 mM) of either NaCl or NaClO_4_. All simulations were run for 200 ns (generating 100,000 timeframes) in the isothermal-isobaric ensemble, with the temperature and pressure maintained using the Nosé–Hoover thermostat^60,61^ and the Parrinello–Rahman barostat.^62^ Further details are given in the SI (Section 6).

Figure 2b shows examples of the anion trajectories obtained from the MD simulations for recipient **1** in a fully extended conformation.^63^ The magenta anionic “clouds” correspond to probability thresholds set to eight times the bulk anion density and were produced using VMD^64^ and rendered with POV-Ray 3.7.^65,66^ These reveal the greater affinity of ClO_4_^–^ over Cl^–^ and how ClO_4_^–^ binding is focused in the “channels” formed by the residue side chains proximal to positively charged groups. This is perhaps most evident with ClO_4_^–^ accumulation around the charged headgroup of K6 (center) and the grooves between the K6 side chain and the side chains of F4 and L8.

We anticipated that the association constants for anion binding would be weak, and this was confirmed by carrying out ^1^H NMR titration experiments in which the amide N–H groups were tracked as a function of the mole fraction of the added salt (SI, Section 3.2). Recipient **2** underwent precipitation during these experiments, so the focus was with recipients **1** and **3** and the two expected extremes of affinity: Cl^–^ and ClO_4_^–^. Chloride proved to be too weak of a binder to obtain reliable affinity data for either peptide. However, *K*_a_ values could be obtained for ClO_4_^–^ association to both peptides (SI, Section 3.2). In the case of **1**, obtained *K*_a_ values ranged from 1–7 M^–1^, with V15 reporting the weakest affinity and K10 reporting the strongest. The binding constants of K6 and K12 were also relatively high (6 M^–1^). The values for each residue were in line with the anion mapping depicted in Figure 2a/b, as well as previous work revealing how the termini of polymers are not sites of anion affinity.^53^ The *K*_a_ values reported by **3** were similarly small, ranging from 2 M^–1^ (V15) to 6 M^–1^ (9S), and generally in line with those of peptide **1**. In summary, more charge-diffuse anions have a small but significant affinity for peptides **1**–**3**, with binding focused near the charge groups and away from the termini.

To determine whether such weak affinity could induce structural development in the peptides, we turned to a combination of CD and NMR spectroscopy and MD simulations. CD spectroscopy (SI, Section 5) for recipients **1** and **3** revealed that different equivalents of ClO_4_^–^ did not change the character of the CD spectrum of each (SI, Section 5). Thus, although in some cases, the minima shifted somewhat, each peptide maintained a negative peak at around 200 nm. TROSY NMR provided a more nuanced view of structure changes that, *en masse*, also demonstrated little in the way of the anion-induced extended structure (SI, Section 3.4). Thus, in the presence of Cl^–^ (ClO_4_^–^), *J* values for peptide **3** were found to be between 5.67 (5.60) and 8.19 (8.75) Hz. For example, in the presence of ClO_4_^–^ residues, H6, A11, E13, and G14 were clustered between 5.60 and 6.37 Hz—indicating essentially random-coil structures—whilst the other residues were clustered between 6.79 and 8.75, reflecting that these residues sampled more extended or helical regions of the Ramachandran space.^42,44^ Overall, Cl^–^ and ClO_4_^–^ each led to higher coupling constants for six residues, and although ClO_4_^–^ had a more significant effect than Cl^–^ (the RMSD relative to the salt-free data were 0.26 and 0.51 for Cl^–^ and ClO_4_^–^, respectively), the small changes did not signify any appreciable development of the extended structure. Similarly, the changes in the signal-shift temperature coefficients of each amide N–H in peptides **1** and **3** as a function of salt indicated only incremental changes in the structure (SI, Section 3.3). Thus, both data sets were linear and displayed a narrow range, and although in the case of ClO_4_^–^, there was only one exception (M5 in **1**) where the temperature coefficients in **1** and **3** did not increase, the average temperature coefficient increases were small. For example, in the case of **3** and ClO_4_^–^, the coefficient only increased from 4.04 to 4.69 ppb/K.

Finally, we extracted the radius of gyration (*R*_G_) of peptides **1**–**3** from the aforementioned molecular dynamics studies in the absence of salt and in the presence of NaCl and NaClO_4_. Although the errors in these calculations led to no significant difference between the absence of salt and the presence of NaCl, the presence of NaClO_4_ did reliably lead to a smaller average particle size over a wide temperature range (SI, Section 6), and correspondingly a change in the *R*_G_ distribution at 298 K. Interestingly, although there was no evidence of the well-defined extended structure in the ClO_4_^–^ data, there was evidence of a bimodal distribution within the pool of structures. Regardless of how these subpools differ, it is evident that ClO_4_^–^ association led to a significant degree of compaction via a combination of anion insertion between positive charges and the formation of weak noncovalent interactions (NCIs) between anions and peptides. However, these interactions did not induce any extended helical structure in the peptides.

We used ^1^H NMR spectroscopy to assess how salts affected the rate of aggregation and precipitation of peptides **1**–**3** (SI, Section 4). In these studies, the concentration of the peptide in D_2_O was 2 mM, in either 50 mM sodium acetate buffer (pD = 5.2) or 50 mM phosphate buffer (pH = 2.3). The loss of peptides to large soluble aggregates and/or precipitates was determined by referencing the methyl group signals of 3V, 8L, and 15V to an external reference (sodium ethyl sulfate). In the preliminary work, it was noted that over the 24 h timeframe of the experiment, the solution of wild-type peptide **1** did not undergo any observable loss in the presence of ClO_4_^–^, regardless of the pH. On the other hand, in the case of peptide **3**, solubility issues precluded studies at pH 5.2, whilst no precipitation was observed in the presence of ClO_4_^–^ at pH = 2.3. In contrast, at both pH values, aggregation of peptide **2** was observed for ClO_4_^–^. These results emphasize the intrinsic differences between how the cationic K, R, and H residues interact (charge-pair) with counteranions.^35^

As a first set of experiments, aggregation data for **2** was collected at pH 5.2 in the presence of 60 mM Cl^–^, I^–^, ClO_4_^–^, ReO_4_^–^, and PF_6_^–^. No loss of peptide was noted for weakly binding Cl^–^, but Figure 3a shows the aggregation profiles (average of three runs) in the presence of the other anions examined. Similarly, Figure 3b shows the effect of different concentrations of ClO_4_^–^ upon peptide **2**.

**Figure 3:**
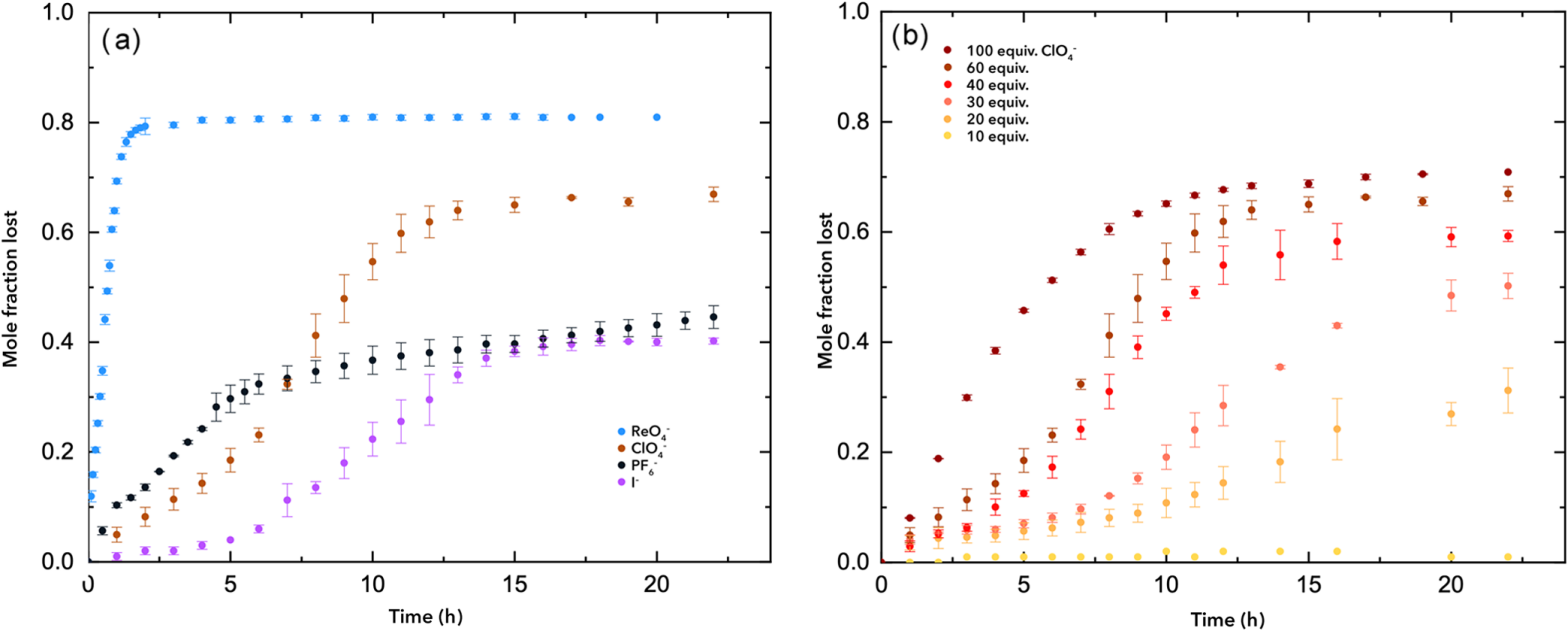
(a) Loss of the soluble, monomeric peptide from a 2 mM solution of recipient **2** in 50 mM sodium acetate buffer (pH = 5.2) in the presence of 120 mM NaCl, NaI, NaClO_4_, NaReO_4_, and NaPF_6_. Aggregation was assessed by monitoring the remaining peptide via integration of the 3V, 8L, and 15V methyl signals with an external reference of sodium ethyl sulfate. (b) Aggregation data for 2 mM recipient **2** in 50 mM sodium acetate buffer (pH = 5.2) in the presence of different concentrations of NaClO_4_.

The formation of peptide fibrils typically involves an initial lag-phase characteristic of nucleation-dependent aggregation (SI, Section 7).^67,68^ Thus, in the case of α-Syn, an initial reversible nucleation step to generate a library of nuclei of *n*-mers leads to the formation of protofibrils, which finally undergo irreversible growth/elongation to form fibrils.^67–72^ We assessed how well the aggregation profile of peptide **2** fitted using the Finke–Watzky (F–W) two-step model of aggregation. The F–W model is a minimalist kinetic model that assumes a slow continuous nucleation process (A → B, *k*_1_) and fast, autocatalytic growth (A + B → 2B, *k*_2_). This approach yields: (a) the induction period (*t*_1_); (b) the time to the maximum rate of growth, *t*_max_ (the inflection point of the curve), and; (c) the plateau time *t*_2_ (SI, Section 4.2). Specifically, to define *t*_1_ and *t*_2_, we used Bentea, Watzky, and Finke’s^73^ approach of identifying where the third derivative (or jerk) of the concentration of the product versus time equals zero. Thus, the induction period, *t*_1_, is defined as the time to maximum acceleration (zero jerk), corresponding to the point of transition between the lag and exponential growth phases, whereas *t*_2_ is the time of greatest deceleration (zero jerk), after *t*_max_, i.e., the point of transition between the exponential growth and the plateau phase.

In attempting to fit the data shown in Figure 3b, we observed that only at intermediate concentrations of ClO_4_^–^ was there a good fit of the data to the F–K model (Table 1). In these cases, the obtained inflection point, *t*_max_, decreased from 11.86 h at 60 mM salt to 7.13 h at 120 mM. The root cause of this change is the much slower nucleation process (*k*_1_) relative to the faster autocatalytic growth (*k*_2_ and *k*_max_). In contrast, the poor fits to the remaining data shown in Figure 3b suggest that at high and low salt concentrations, the rates of nucleation and autocatalytic growth are similar. Examining the fit of the data from the other salts at pH = 5.2 leads to similar conclusions. Thus, relative to ClO_4_^–^, I^–^, and ReO_4_^–^ are weak and strong promoters of precipitation (the reverse Hofmeister effect).^35^ Correspondingly, in the conditions examined, only relatively high I^–^ concentrations (120 mM) and low ReO_4_^–^ concentrations (20 mM) yielded good fits to the F–K model.

**Table 1:** Kinetic Parameters from the Finke–Watzky (F–W) Two-Step Model for the Aggregation of Recipient **2** (2 mM) in the Presence of a ClO_4_^–^ Anion

**ClO**_**4**_^**–**^**conc**. (mM)	***k***_**1**_ (h^–1^)	***t***_**1**_ (jerk, h)	***k***_**max**_ (mM^–1^ h^–1^)	***t***_**max**_ (h)	***k***_**2**_ (mM^–1^ h^–1^)	***t***_**2**_ (h)
**60**	0.01	6.38	0.13	11.86	0.11	17.34
**80**	0.03	3.47	0.14	8.68	0.11	13.90
**120**	0.03	2.57	0.16	7.13	0.13	11.68

We also investigated recipient **2** at pH = 2.3. Here, it is evident that much higher concentrations were required to induce precipitation. For example, 200 mM ClO_4_^–^ was required to induce a fast and classically sigmoidal aggregation process (*t*_max_ = 2.31 h). This data fit well because of a very slow nucleation process and a much faster autocatalytic growth step, suggesting that the former is more influenced by charge repulsion at the lower pH value than the latter.

Fitting for the data from the other anions investigated is shown in the Supporting Information (Section 4.2). In some cases, a considerable amount of precipitate was formed upon the addition of salt. This was particularly the case with ReO_4_^–^, and we confirmed the presence of the anion in the precipitate by using X-ray energy-dispersive spectroscopy (X-EDS; SI, Section 4.3). For example, using a sample of recipient **2** precipitated with ReO_4_^–^; both point- and area-elemental analyses confirmed the presence of rhenium. Thus, ReO_4_^–^ is a useful precipitator of proteins not only because it does so relatively quickly and at such low concentrations but also because it is theoretically possible to readily use techniques such as X-EDS to map for heterogeneities within samples.

## Conclusions

Our studies with the 15-mer *N*-terminal peptide of α-synuclein (α-Syn) and two mutants in which the three Lys residues of the wild type were replaced with either Arg or His residues have revealed a number of key points. First, although phospholipid membrane binding induces helicity in α-Syn, monovalent anions do not induce significant long-range ordering of the essentially random-coil 15-mer. Charge-diffuse anions do nevertheless bind to the three peptides, especially to triple Arg peptide **2**. Where binding could be studied, association was found to be at the midsections of the sequences and away from the positively charged *N*-terminus (and as expected, the negatively charged *C*-terminus). Binding constants were obtained for the more strongly associating anions and were found to be as high as 10 mM^–1^. Presumably, binding constants to **2** are even higher but aggregation prevented quantification. Despite no significant long-range structure development with anion binding, MD simulations did reveal a compaction of the peptides in the presence of ClO_4_^–^. Moreover, the combination of NMR spectroscopy and MD supported the conclusion that although anions do not have one specific binding site, association is focused on the midsection of the peptide between positively charged residues and their nearest neighbors. The effect of this binding is to reduce the effective net positive charge of the peptide and induce compaction and ultimately the formation of large, soluble *n*-mers and/or precipitates. Finally, our aggregation studies reveal that in general the reverse Hofmeister effect is followed and that at intermediate salt concentrations, aggregation follows the Finke–Watzky two-step model.

## Methods

Peptides were obtained from Genescript with a certified purity of 98%. Each was purified via a two-step process of anion exchange and size exclusion chromatography. Structural characterization involved a sequence of 2D NMR spectroscopy experiments, namely, total correlation spectroscopy (TOCSY), rotating-frame Overhauser effect spectroscopy (ROESY), correlation spectroscopy (COSY), and ^1^H–^15^N heteronuclear single quantum correlation (HSQC). CD spectra were obtained using a Jasco J-810 spectropolarimeter. Elemental analysis was conducted using X-ray energy-dispersive spectroscopy (X-EDS) with an Oxford Instruments system and AZtec software.

Anion binding was investigated with ^1^H NMR, as were temperature-dependent chemical shifts. TROSY NMR was used to measure the coupling constants to determine each φ angle. For the aggregation studies, fitting relied on the Finke–Watzky model.

All simulations in this work were carried out using GROMACS 2016.3. For the replica exchange simulations, the peptides were modeled using the Amber-03ws all-atom force field, water was modeled using the TIP4P2005 potential, the ions were modeled using the generalized Amber force field (GAFF), and their partial charges were obtained from AM1-BCC calculations.

## Supplementary Material



## Notes

Materials and instrumentation, peptide characterization, chemical shift data with excess salts, aggregation studies and data fitting, elemental analyses, circular dichroism, and details of the computational work (PDF)
