# Supplementary material for: Anion Binding and Aggregation of N‑Terminal α‑Synuclein Peptides
Source: ACS Omega. 2025 May 21;10(21):22216–23. doi: 10.1021/acsomega.5c02618 (PMC12138707; doi:10.1021/acsomega.5c02618)
Supplement: Supplementary file 1 [file ao5c02618_si_001.pdf]

Supporting Information

Anion binding and aggregation of *N*-terminal α-Synuclein peptides

Ruiqing Wang, Busayo D Alagbe, Henry S. Ashbaugh,[2] Bruce C. Gibb[1]

[1]Department of Chemistry, Tulane University, New Orleans, LA 70118, USA.

[2]Department of Chemical and Biomolecular Engineering, Tulane University, New Orleans, LA, 70118, USA

Ruiqing Wang: https://orcid.org/0009-0007-8978-4153

Busayo D Alage: https://orcid.org/0000-0003-1513-5015

Henry S. Ashbaugh: https://orcid.org/0000-0001-9869-1900

Bruce C. Gibb: https://orcid.org/0000-0002-4478-4084

Table of Contents

1. Materials and instrumentation 3
2. 2D NMR characterization of peptides 3
   2.1 Wild-type backbone N-H peak assignment 3
   2.2 Arginine mutant backbone N-H peak assignment 5
   2.3 Histidine mutant backbone N-H peak assignment 7
3. Binding studies 9
   3.1 Chemical shift data with excess salt 9
   3.2 Binding constant determinations 12
   3.3 VT NMR experiments of peptides with/without salts 24
   3.4 Peptide conformations define from *J*-coupling constants 32
4. Aggregation studies 33
   4.1 Aggregation of arginine mutant with salts 33
   4.2 Aggregation curve fitting 48
   4.3 Elemental analysis of arginine mutant aggregation particles 53
5. Circular Dichroism studies 57
   5.1 Three peptide derivatives at pH 2.3 57
   5.2 CD spectra in response to added anions 57
6. Computational studies 59
   6.1 R_g characterization 59
   6.2 RMSD characterization 61
   6.3 Anion binding visualization 66
8. References 69

1. Materials and instrumentation

The peptides were procured from Genescript with a certified purity of 98%. Each peptide was purified via a two-step process of anion exchange and size exclusion chromatography and confirmed by ^{1}H NMR (see below). The peptides corresponded to the initial fifteen residues of α-synuclein (^{1}MDVFMKGLSKAKEGV$^{15;}$ α-Syn$_{15}$), a triple arginine mutant (^{1}MDVFMRGLSRARE GV^{15}; α-SynR), and a triple histidine mutant (^{1}MDVFMHGLSHAHEGV^{15}; α-SynH. Their sequences are shown below (Figure S1).

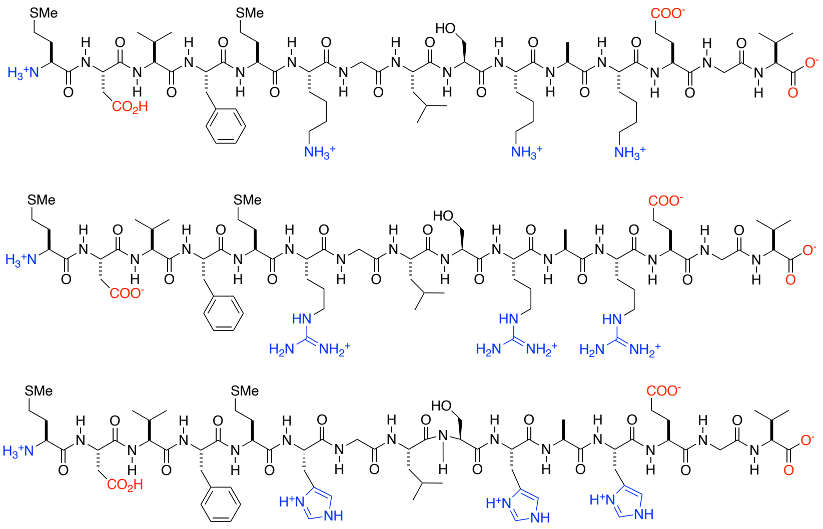

Figure S1: Structures of the wild-type α-Syn$_{15}$ (*top*), arginine mutant α-SynR (*middle*), and histidine mutant α-SynH (*bottom*). Positively and negatively charged resides are shown in blue and red.

2. 2D NMR characterization of peptides

A sequence of 2D NMR spectroscopy experiments, namely Total Correlation Spectroscopy (TOCSY), Rotating-Frame Overhauser Effect Spectroscopy (ROESY), Correlation Spectroscopy (COSY), and ^{1}H-^{15}N Heteronuclear Single Quantum Correlation (HSQC), was employed to characterize each peptide. These investigations were conducted utilizing a 700 MHZ spectrometer located at Louisiana State University (LSU). For aggregation studies, 1D NMR experiments were conducted on a 400 MHz NMR at Tulane University. All peptide solutions were 5 mM, utilizing either 50 mM phosphate buffer (pH = 2.3) or 50 mM sodium acetate (pH = 5.2) in 9:1 H_2O:D_2O.

2.1 Wild-type backbone N-H peak assignment

The HSQC, TOCSY, ROSY, and ^{1}H NMR spectra of the wild-type α-synuclein$_{15}$ (α-Syn$_{15}$) at pH 2.3 are shown below. All samples were run at 25°C. Following this is a summary table of chemical shift data.

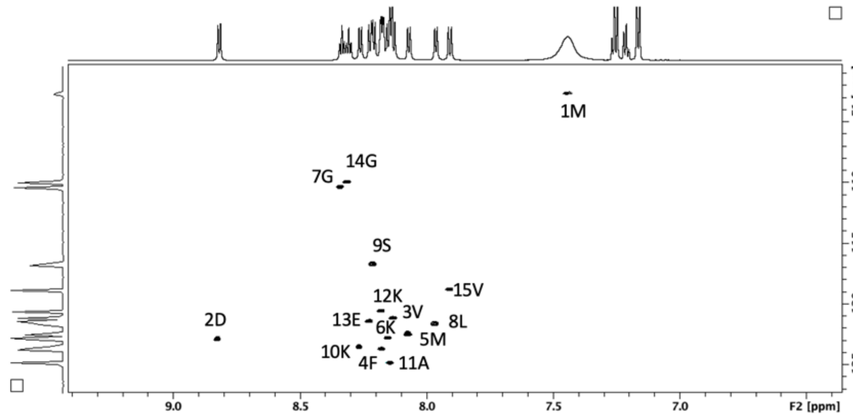

Figure S2: Assignment of backbone N-H in ^{1}H-^{15}N HSQC NMR of wild-type peptide (α-Syn$_{15}$) at pH 2.3.

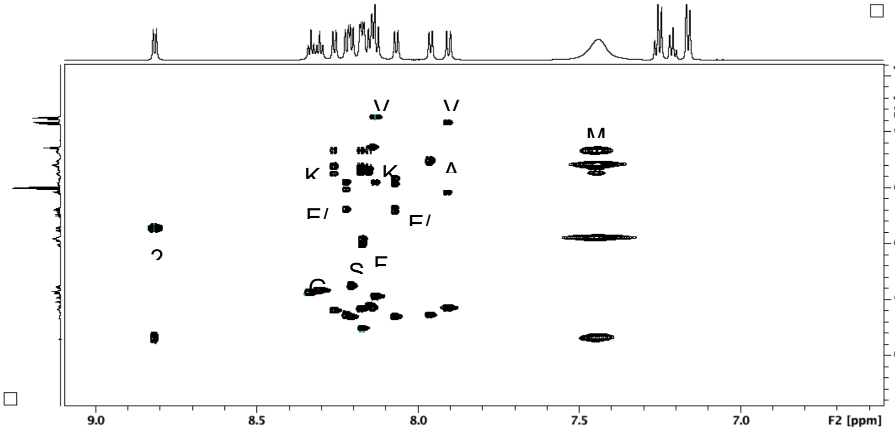

Figure S3: General assignment of backbone N-H in TOCSY NMR of wild-type peptide (α-Syn$_{15}$) at pH 2.3.

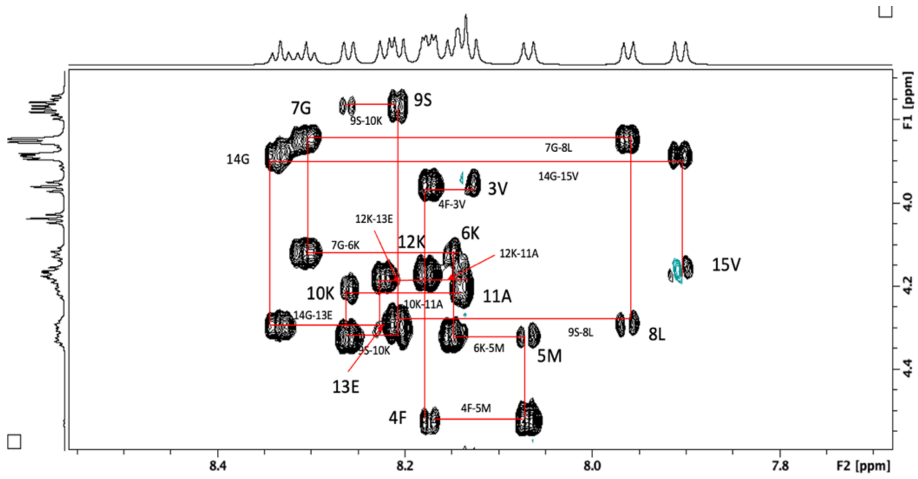

Figure S4: Cross-peak assignment of backbone N-H in ROESY NMR of wild-type peptide (α-Syn$_{15}$).

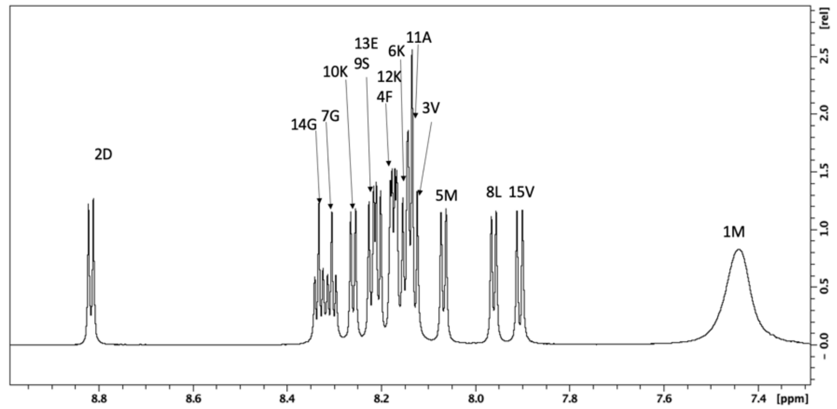

Figure S5: Assignment of the amide ^{1}H NMR signals of the wild-type peptide α-Syn$_{15}$ at pH 2.3. Water suppression utilized the Watergate suppression pulse sequence.

Table S1: Chemical shift data of the wild-type α-Syn$_{15}$ at 25°C and pH at 2.3.

Residual	^{15}N	^{1}H^{N}	^{1}H$^{\alpha}$	^{1}H$^{\beta}$	^{1}H$^{\gamma}$	^{1}H$^{\delta}$
M1	--	--				
D2	122.94	8.82	4.72	2.75		
V3	122.11	8.13	3.98	1.93	0.75	
F4	123.64	8.18	4.64	2.99		
M5	122.43	8.07	4.32	2.41	1.96	1.82
K6	122.79	8.15	4.14	1.76	1.72	1.36
G7	109.85	8.31	3.85			
L8	121.55	7.96	4.29	1.59	1.51	0.78
S9	116.69	8.21	4.34	3.78		
K10	123.5	8.26	4.22	1.78	1.63	1.35
A11	124.81	8.15	4.17	1.31		
K12	120.55	8.18	4.17	1.74	1.68	1.37
E13	121.35	8.23	4.31	2.41	2.06 (1.90)	
G14	110.31	8.34	3.89			
V15	118.79	7.91	4.18	2.09	0.86	

2.2 Arginine mutant backbone N-H peak assignment

The HSQC, TOCSY, ROSY, and ^{1}H NMR spectra of the arginine mutant (α-SynR) at pH 2.3 are shown below. All samples were run at 25°C. Following this is a summary table of the chemical shift data.

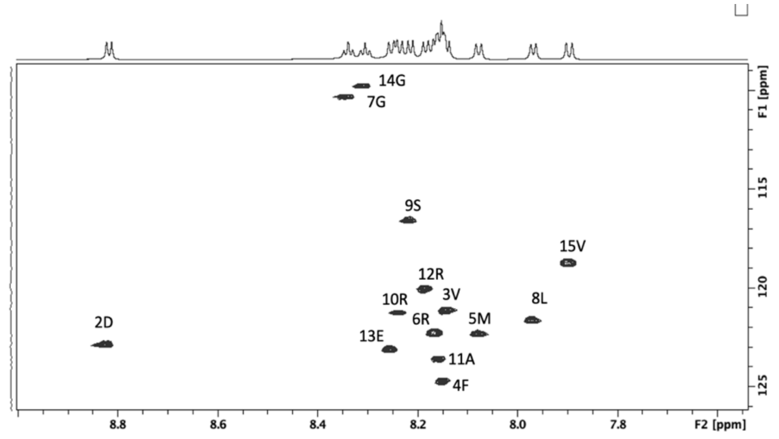

Figure S6: Assignment of backbone N-H in ^{1}H-^{15}N HSQC NMR of the arginine mutant (α-SynR) at pH 2.3.

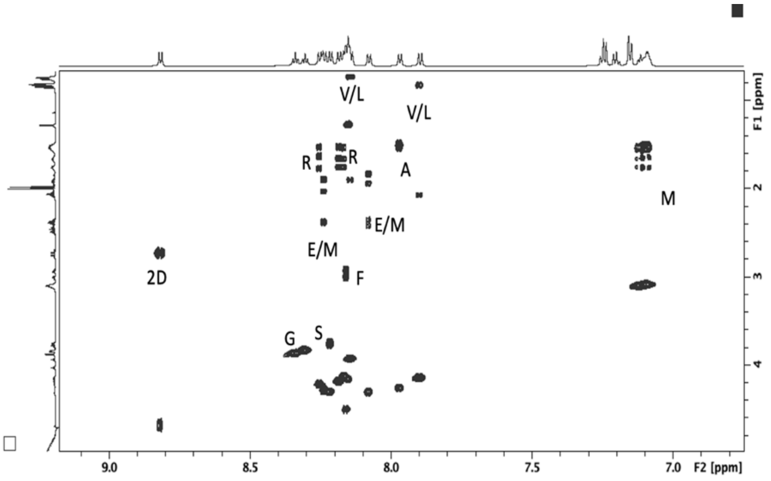

Figure S7: General assignment of backbone N-H in TOCSY NMR of the arginine mutant (α-SynR) at pH 2.3.

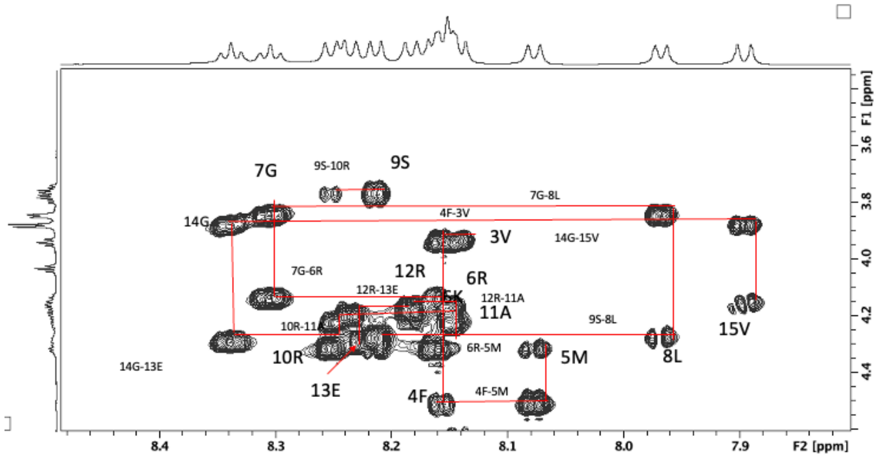

Figure S8: Cross-peak assignment of backbone N-H in ROESY NMR of the arginine mutant (α-SynR).

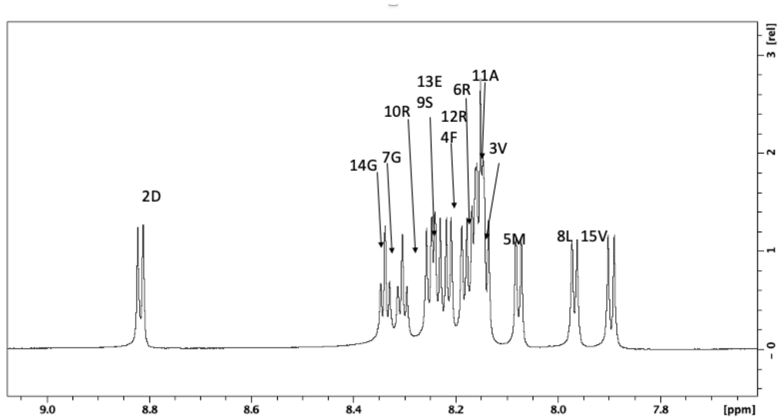

Figure S9: Assignment of the amide ^{1}H NMR signals of the arginine mutant (α-SynR) at pH 2.3. Water suppression utilized the Watergate suppression pulse sequence.

Table S2: Chemical shift of the arginine mutant peptide (α-SynR) at 25°C and pH at 2.3.

Residual	^{15}N	^{1}H^{N}	^{1}H$^{\alpha}$	^{1}H$^{\beta}$	^{1}H$^{\gamma}$	^{1}H$^{\delta}$
M1	--	--				
D2	122.91	8.84	4.70	2.74		
V3	121.18	8.14	3.95	1.92	0.75	
F4	124.72	8.15	4.52	2.98		
M5	122.23	8.08	4.31	2.42	1.94	1.85
R6	122.28	8.17	4.14	1.77	1.68	1.55
G7	109.72	8.32	3.86			
L8	122.62	7.98	4.28	1.57	1.49	0.75
S9	116.55	8.22	4.32	3.78		
R10	121.34	8.24	4.25	1.79	1.69	1.58
A11	123.59	8.16	4.18	1.27		
R12	120.02	8.18	4.20	1.77	1.70	1.54
E13	123.13	8.25	4.29	2.40	2.03 (1.90)	
G14	110.41	8.34	3.88			
V15	118.80	7.90	4.17	2.08	0.84	

2.3 Histidine mutant backbone N-H peak assignment

The HSQC, TOCSY, ROSY, and ^{1}H NMR spectra of the histidine mutant peptide (α-SynH) at pH 2.3 are shown below. All samples were run at 25°C. Following this is a summary table of chemical shift data.

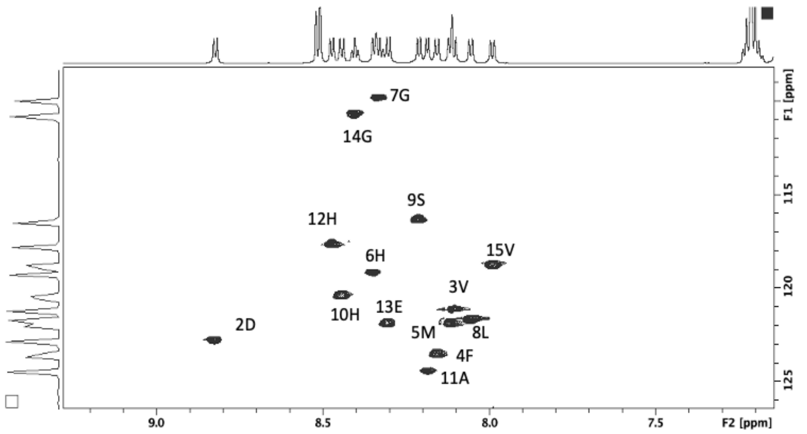

Figure S10: Assignment of backbone N-H in ^{1}H-^{15}N HSQC NMR of the histidine mutant (α-SynH) at pH 2.3.

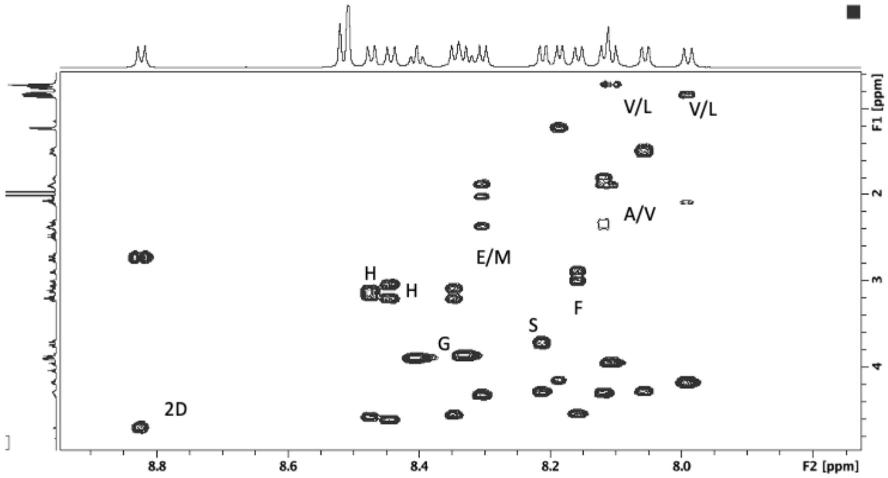

Figure S11: General assignment of backbone N-H in TOCSY NMR of the histidine mutant (α-SynH) at pH 2.3.

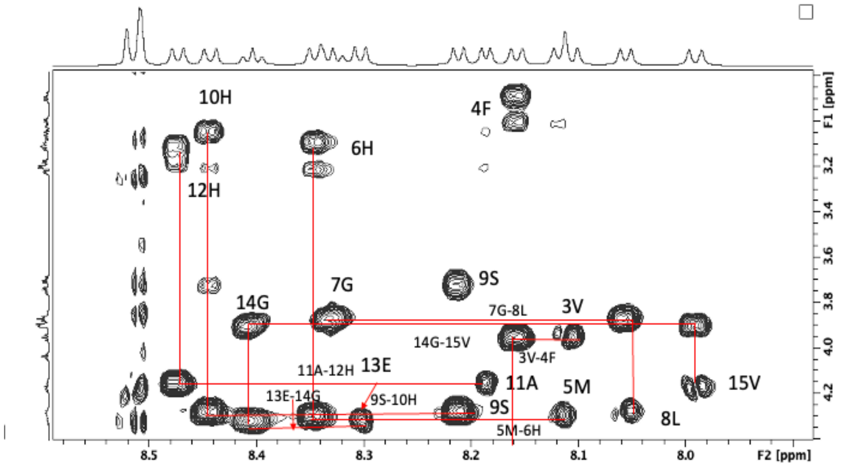

Figure S12: Cross-peak assignment of backbone N-H in ROESY NMR of the histidine mutant (α-SynH).

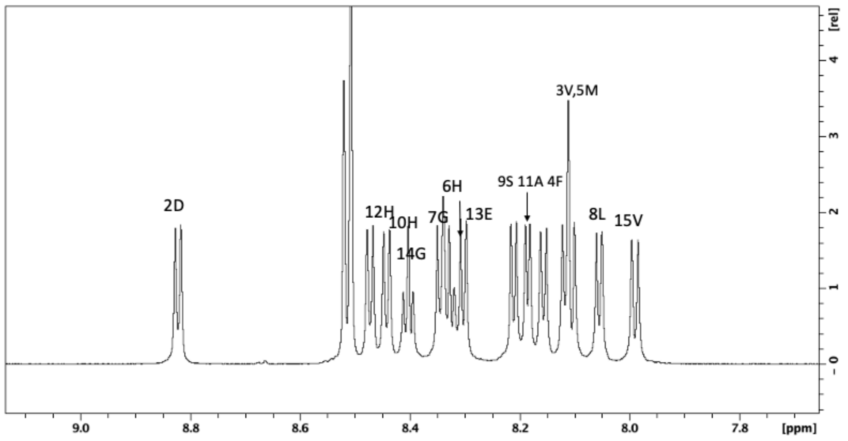

Figure S13: Assignment of the amide ^{1}H NMR signals of the the histidine mutant (α-SynH) at pH 2.3. Water suppression utilized the Watergate suppression pulse sequence.

Table S3: Chemical shift of the histidine mutant (α-SynH) at 25°C and pH at 2.3.

Residual	^{15}N	^{1}H^{N}	^{1}H$^{\alpha}$	^{1}H$^{\beta}$	^{1}H$^{\gamma}$	^{1}H$^{\delta}$
M1	--	--				
D2	122.79	8.82				
V3	121.15	8.11	3.90	1.96	0.71	
F4	123.47	8.15	4.54	2.94		
M5	121.15	8.11	4.30	2.31	1.89	1.82
H6	119.32	8.35	4.58	3.22		3.10
G7	109.77	8.33	3.87			
L8	121.59	8.05	4.26	1.53	1.45	0.78
S9	116.24	8.21	4.31	3.72		
H10	120.32	8.44	4.62	3.25		3.03
A11	124.39	8.19	4.18	1.22		
H12	117.60	8.47	4.61	3.17		3.11
E13	121.75	8.31	4.35	2.38	2.04 (1.91)	
G14	110.65	8.40	3.88			
V15	118.68	7.99	4.20	2.10	0.84	

3. Binding studies

The effects of the addition of a series of sodium salts were investigated with ^{1}H NMR. Initial studies simply added an excess of salt. The peptides investigated included the wild-type α-Syn$_{15}$ at pH 2.3 and 5.2, and the triple histidine-mutant α-SynH at pH 2.3. All data was collected at 25°C.

3.1 Chemical shift data with excess salt

The chemical shifts (Δδ) arising from the addition of 93 equivalents of NaCl, NaBr, NaI, and $NaClO_4$ are summarized below in table form and using bubble maps (Figures S14-S16). In these maps, the area of each circle is proportional to the chemical shift of each N-H upon addition of salt. A red (blue) circle signifies a negative (positive) chemical shift upfield (downfield).

Table S4: Δδ of the N–H signals of the wild-type α-Syn$_{15}$ with 93 equiv. of various salts at 25°C and pH at 2.3.

aa/Δδ(ppm)	NaCl	NaBr	NaI	$NaClO_4$
2D	0.028	0.003	-0.038	-0.120
3V	0.014	-0.010	-0.032	-0.086
4F	-0.004	-0.011	-0.036	-0.102
5M	0.013	0.001	-0.029	-0.090
6K	0.008	-0.022	-0.048	-0.109
7G	0.020	0.000	-0.039	-0.100
8L	0.000	-0.013	-0.042	-0.092
9S	0.006	-0.003	-0.043	-0.087
10K	-0.009	-0.042	-0.095	-0.155
11A	0.008	-0.007	-0.036	-0.099
12K	0.010	-0.034	-0.080	-0.136
13E	0.006	-0.019	-0.029	-0.101
14G	0.008	-0.009	-0.030	-0.080
15V	0.014	0.006	-0.003	-0.048

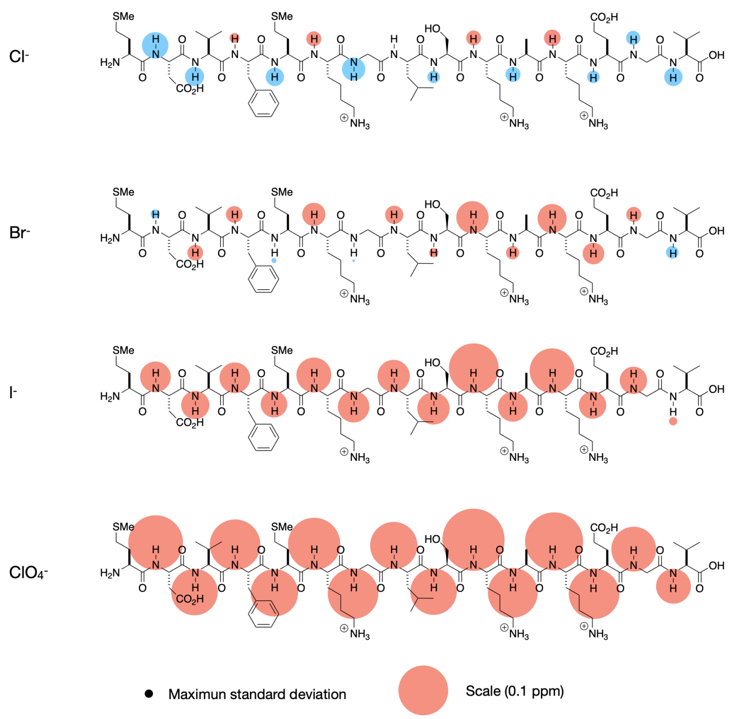

Figure S14: Chemical shift of backbone amide N-H groups of wild-type αSyn$_{15}$ with various salts at pH 2.3. A scale bubble is shown at the foot of the figure. Errors are ± 0.005 ppm.

Table S5: Δδ of the N–H signals of the wild-type α-Syn_{15} with 93 equiv. various salts at 25°C and pH at 5.2.

aa/Δδ(ppm)	NaCl	NaBr	NaI	$NaClO_4$
2D	0.025	0.021	-0.001	-0.040
3V	0.010	0.010	-0.007	-0.033
4F	0.017	-0.002	0.011	-0.027
5M	0.020	0.021	0.010	-0.034
6K	-0.010	-0.014	-0.017	-0.066
7G	0.025	0.012	-0.027	-0.079
8L	0.009	0.000	-0.027	-0.069
9S	0.005	-0.002	-0.005	-0.055
10K	-0.015	-0.018	-0.035	-0.097
11A	-0.006	-0.016	-0.029	-0.075
12K	-0.005	-0.015	-0.031	-0.078
13E	-0.007	-0.009	-0.024	-0.053
14G	0.023	0.023	0.015	-0.010
15V	0.031	0.035	0.027	-0.007

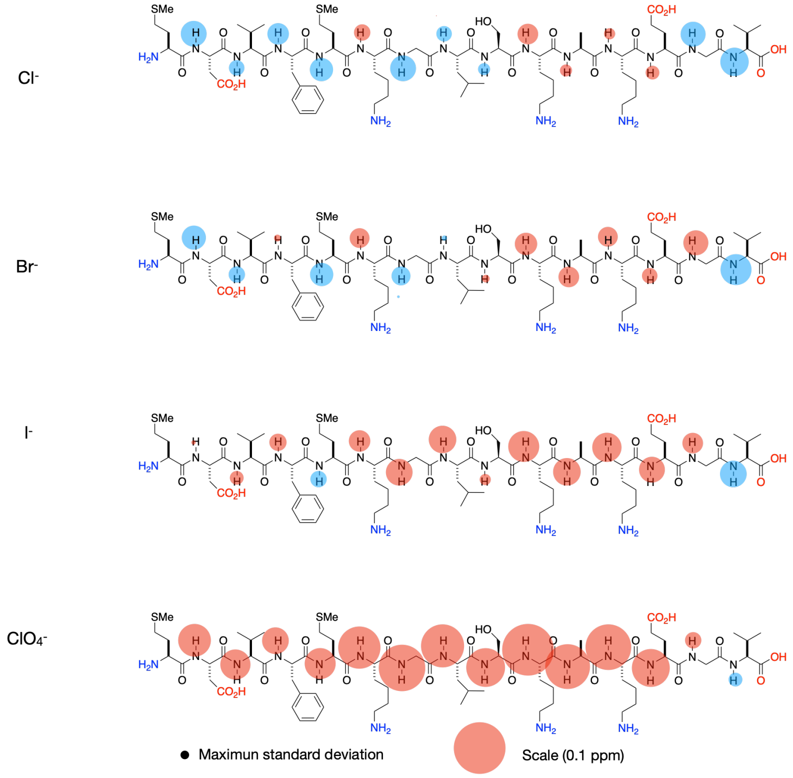

Figure S15: Chemical shift of backbone amide N-H groups of wild-type αSyn_{15} with various salts at pH 5.2. A scale bubble is shown at the foot of the figure. Errors are ± 0.005 ppm.

Table S6: Δδ of the N–H signals of the histidine-mutant α-SynH with 93 equiv. salts at 25°C and pH at 2.3.

Aa/Δδ(ppm)	NaCl	NaBr	NaI	$NaClO_4$
2D	0.032	0.006	-0.036	-0.035
3V	0.015	-0.006	-0.032	-0.050
4F	0.018	-0.006	-0.039	-0.059
5M	0.020	-0.011	-0.032	-0.036
6H	0.008	-0.017	-0.049	-0.059
7G	0.020	-0.012	-0.041	-0.056
8L	0.007	-0.011	-0.043	-0.050
9S	0.014	-0.009	-0.039	-0.048
10H	-0.012	-0.041	-0.083	-0.095
11A	0.001	-0.017	-0.041	-0.051
12H	0.006	-0.017	-0.053	-0.069
13E	0.015	-0.003	-0.027	-0.034
14G	0.021	-0.009	-0.013	-0.012
15V	0.017	0.014	-0.005	-0.010

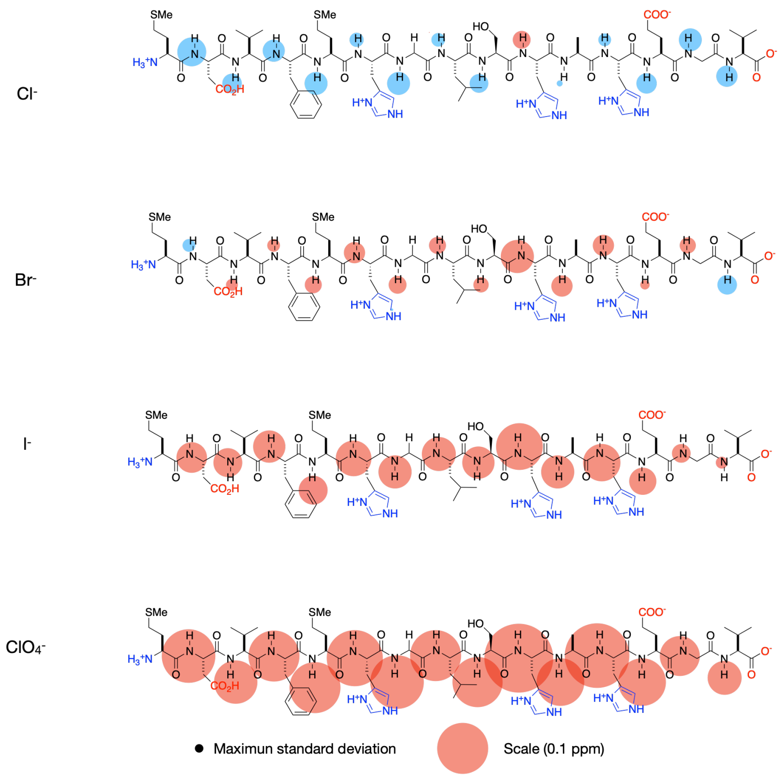

Figure S16: Chemical shift of backbone amide N-H groups of histidine-mutant aSyn$_{15}$ with various salts of at pH 2.3. A scale bubble is shown at the foot of the figure. Errors are ± 0.005 ppm.

3.2 Binding constant determinations

To determine anion affinity, we selected two representative salts: NaCl and $NaClO_4$. These titrations involved monitoring N-H signal shifts as a function of the peptide-salt ratio. In each study the peptide concentration was 5 mM, and the salt ranged from 0-93 eq. All data was collected at 25°C. Using the methyl signal shifts from 3V, 8L and 15V as internal references, fitting to the 1:1 model was possible with $NaClO_4$. However, binding of Cl^- was too weak for a reasonable fit.

For both the wild-type peptide and the triple histidine mutant, the following is shown: 1) Stacks of the resultant NMR spectra; 2) fitting data and their corresponding residuals for $NaClO_4$. (BindFit, http://app.supramolecular.org), and; 3) the obtained binding constants.

a) wild-type α-Syn$_{15}$

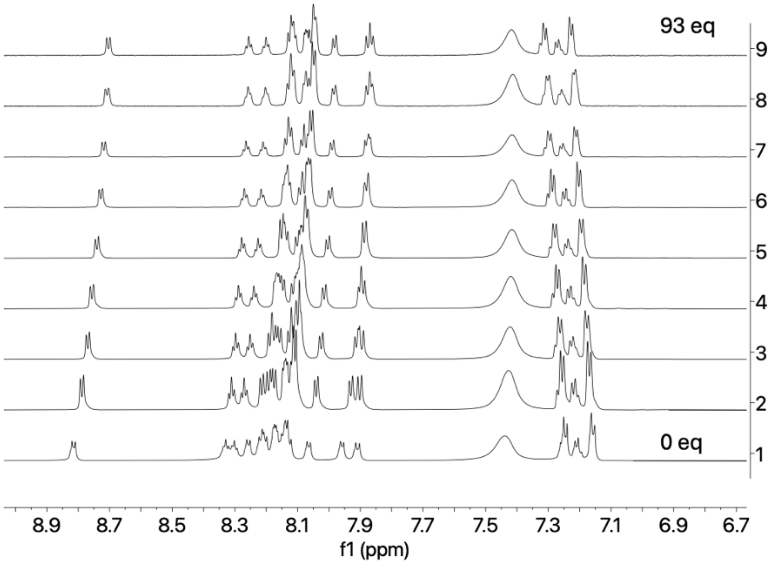

Figure S17: ^{1}H NMR chemical shift of the amide N–H signals of the wild-type peptide titrated with $NaClO_4$ at pH 2.3.

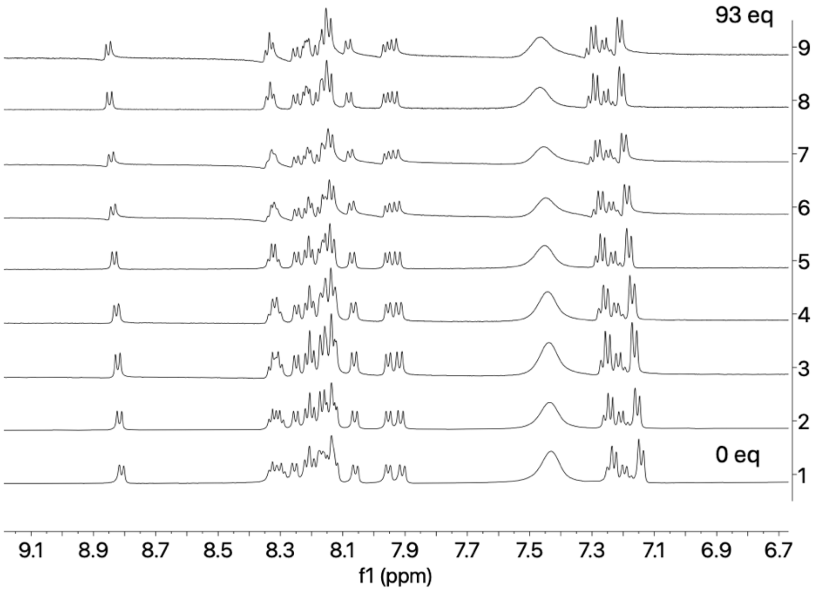

Figure S18: ^{1}H NMR chemical shift of the amide N–H signals of the wild-type peptide titrated with NaCl at pH 2.3.

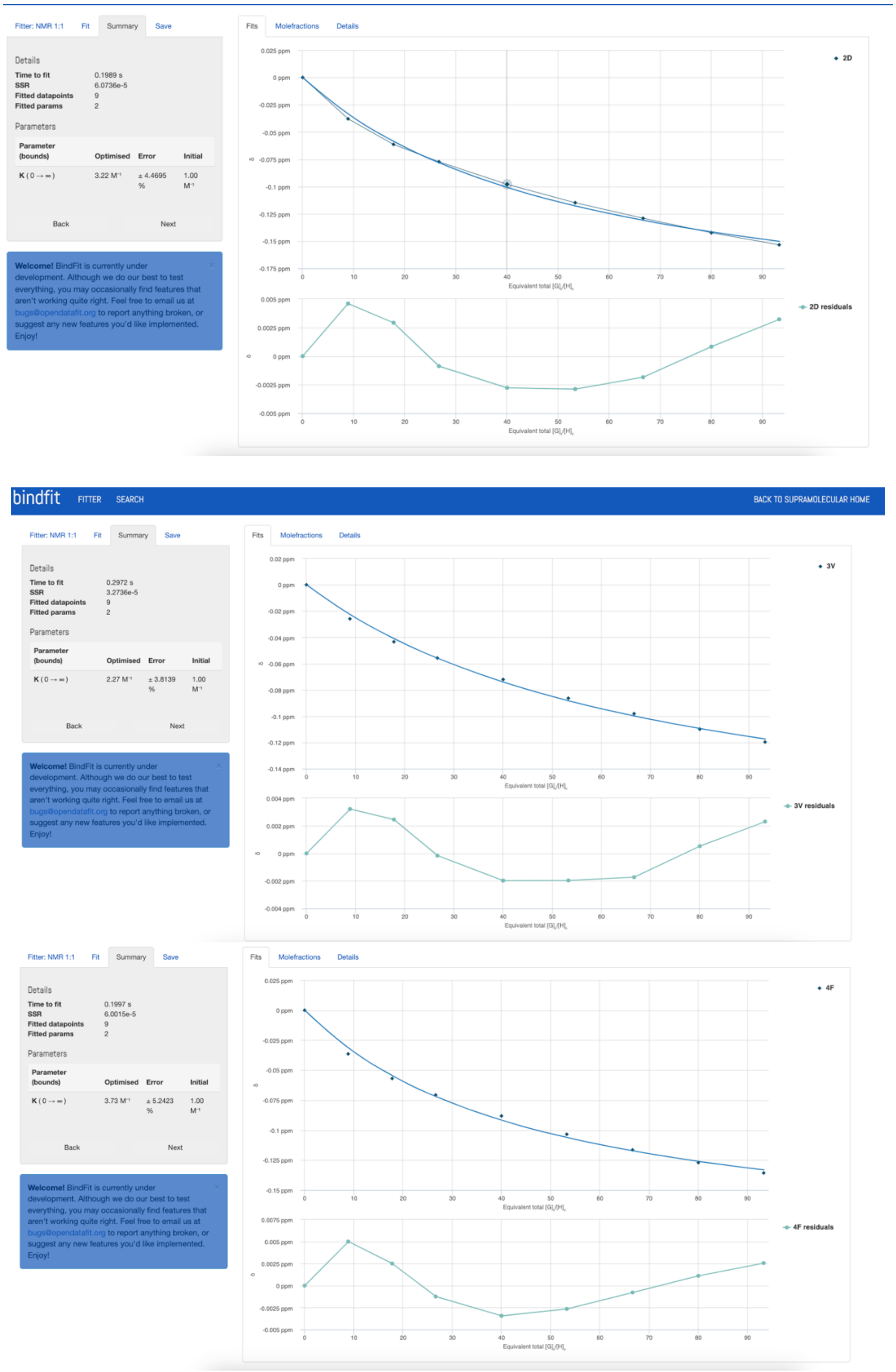

Figure S19: Fitting data of wild-type backbone amide N-H group chemical shifts (top: 2D, middle: 3V, bottom F4) titrated with $NaClO_4$ at pH 2.3.

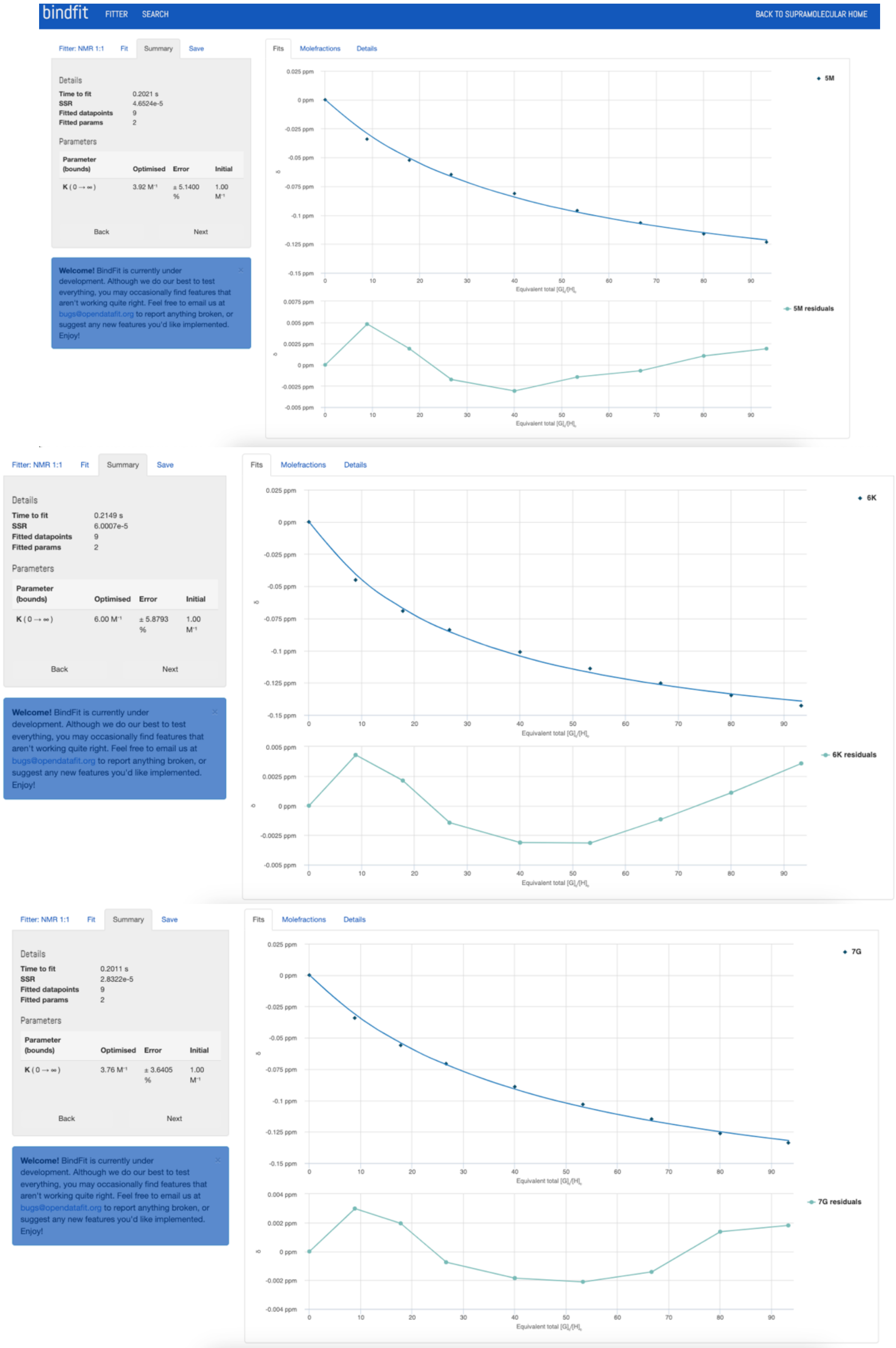

Figure S20: Fitting data of wild type backbone amide N-H group chemical shifts (top: 5M, middle 6K, bottom: 7G) titrated with $NaClO_4$ at pH 2.3.

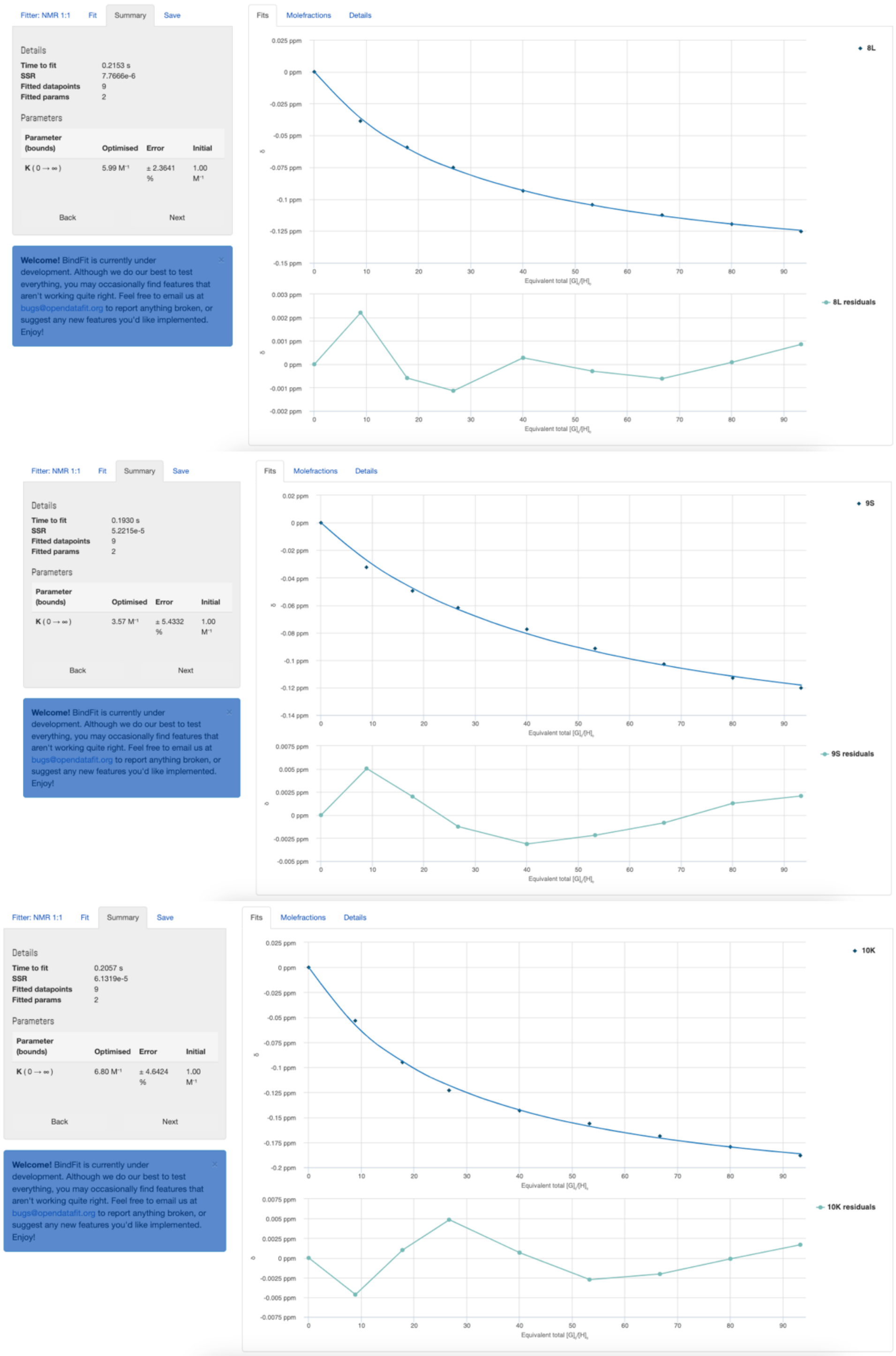

Figure S21: Fitting data of wild type backbone amide N-H group chemical shifts (top: 8L, middle 9S, bottom: 10K) titrated with $NaClO_4$ at pH 2.3.

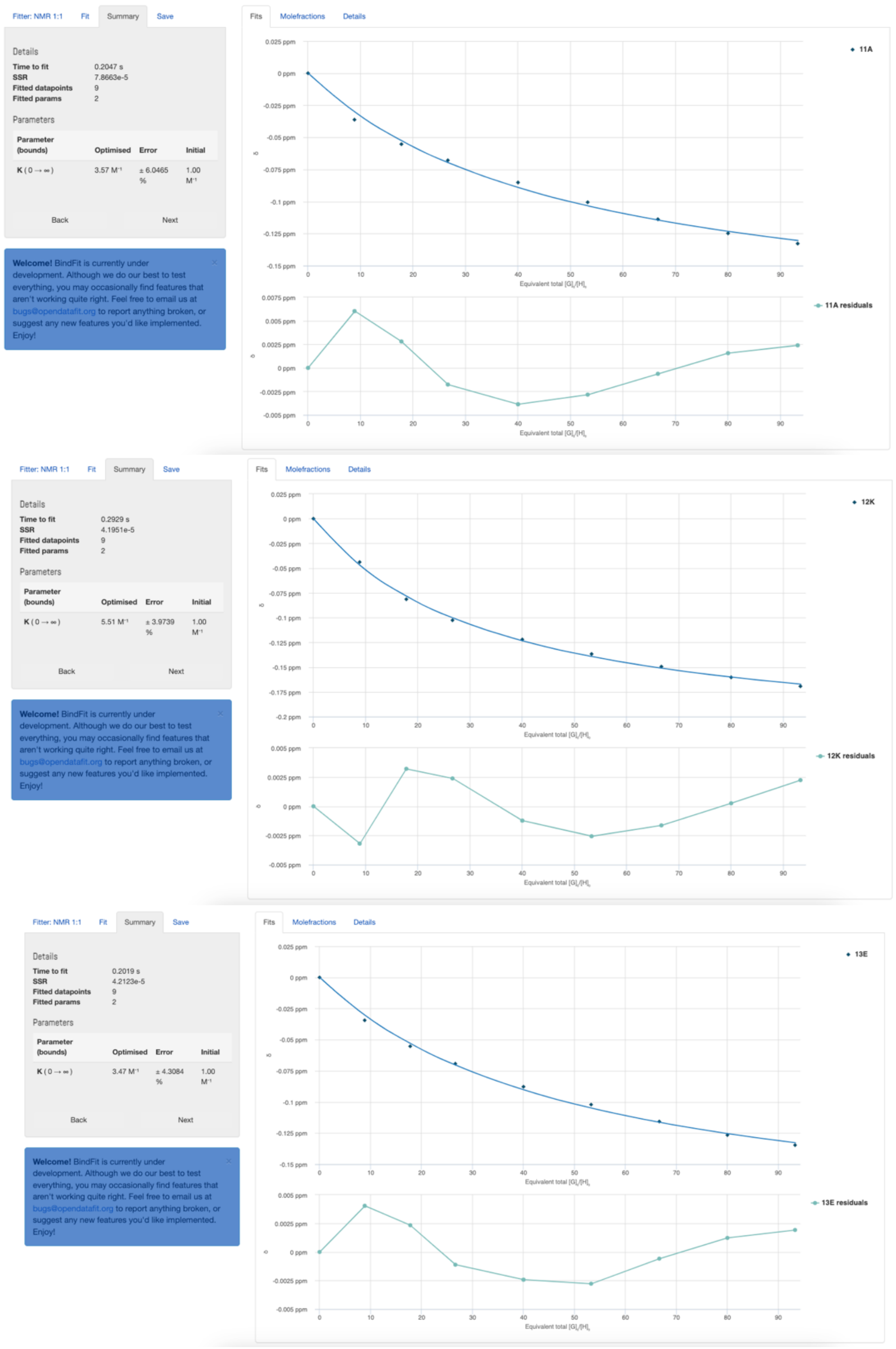

Figure S22: Fitting data of wild type backbone amide N-H group chemical shifts (top: 11A, middle 12K, bottom 13E) titrated with $NaClO_4$ at pH 2.3.

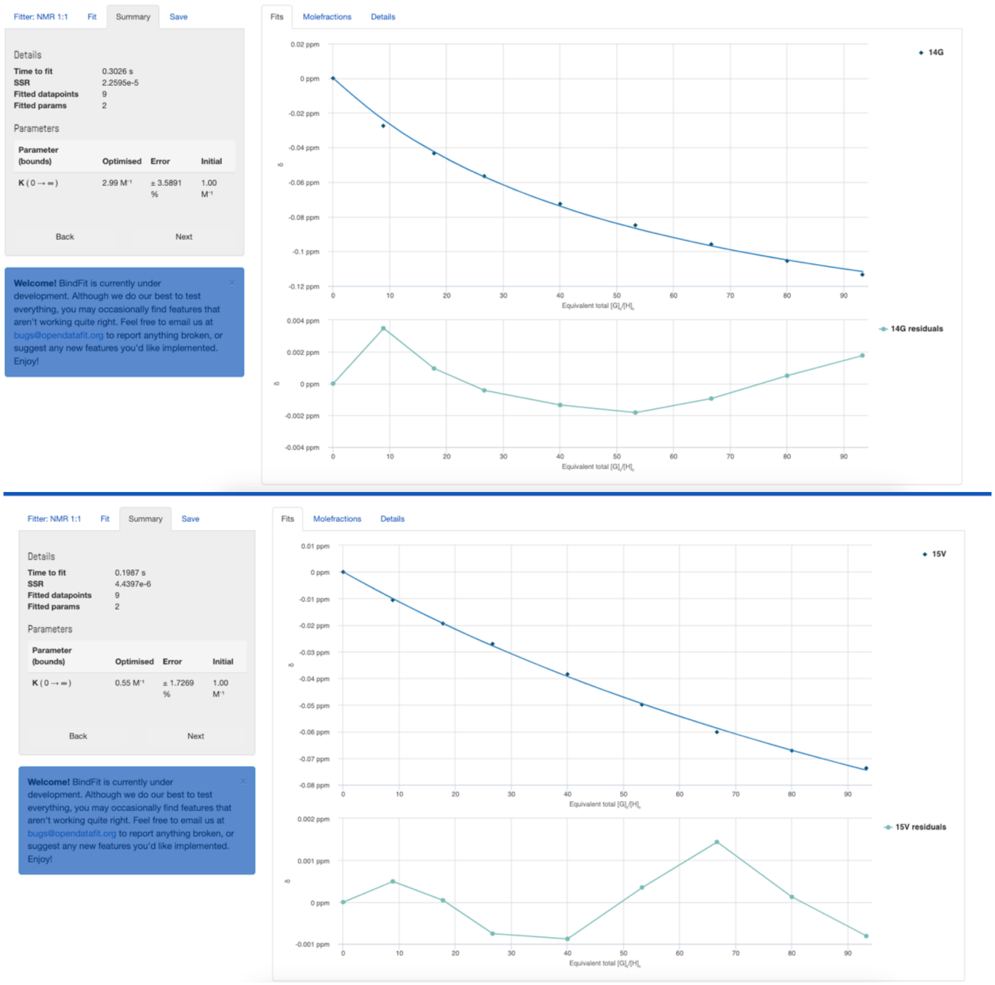

Figure S23: Fitting data of wild type backbone amide N-H group chemical shifts (top: 14G, bottom: 15V) titrated with $NaClO_4$ at pH 2.3.

The resulting binding constants for ClO_4^- are shown in Table S7:

Table S7: Binding constant of α-Syn$_{15}$ with 93 equiv. of $NaClO_4$ at 25°C and pH at 2.3.

	**2D**	**3V**	**4F**	**5M**	**6K**	**7G**	**8L**
***K*$_a$**	3	2	4	4	6	4	6
**Error (%)**	5	4	5	5	6	4	2
	**9S**	**10K**	**11A**	**12K**	**13E**	**14G**	**15V**
***K*$_a$**	4	7	4	6	4	3	1
**Error (%)**	5	5	6	4	4	4	2

b) Triple histidine-mutant α-SynH

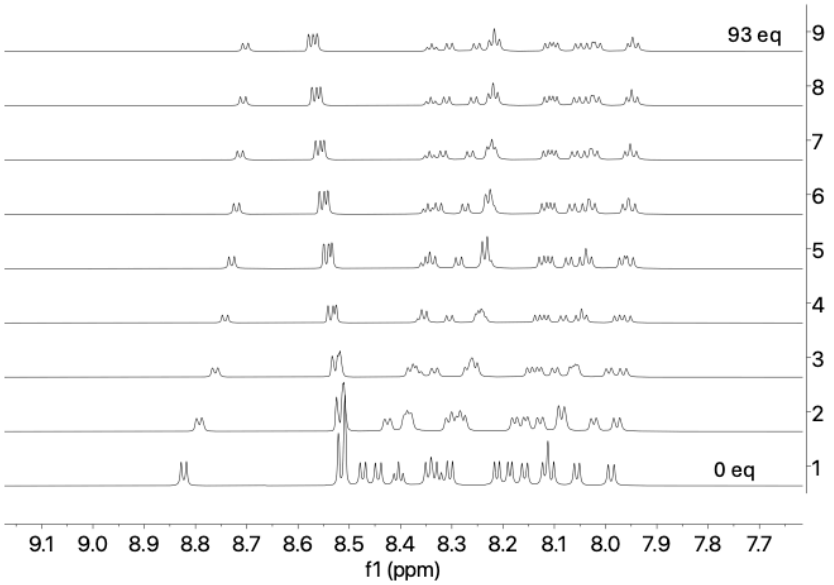

Figure S24: ^{1}H NMR chemical shift of the amide signals of the histidine-mutant peptide titrated with $NaClO_4$ at pH 2.3.

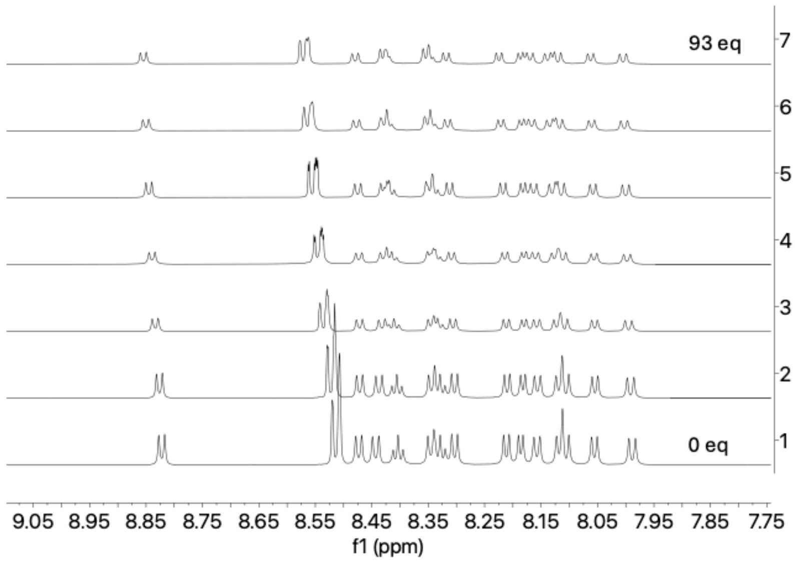

Figure S25: ^{1}H NMR chemical shift of the amide signals of the histidine mutant peptide titrated with NaCl at pH 2.3.

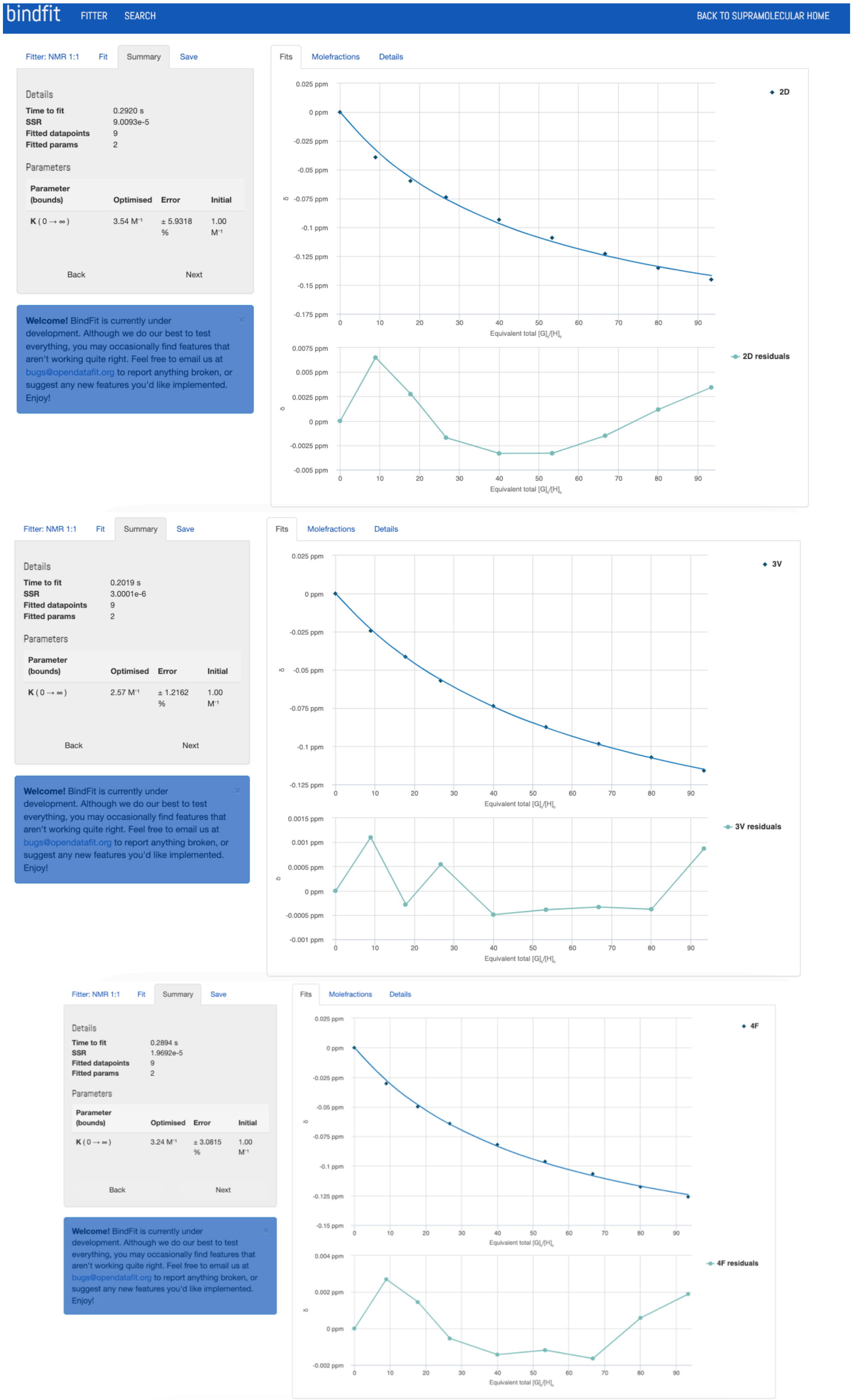

Figure S26: Fitting data of histidine mutant backbone amide N-H group chemical shifts (top: 2D, middle: 3V, bottom 4F) titrated with $NaClO_4$ at pH 2.3.

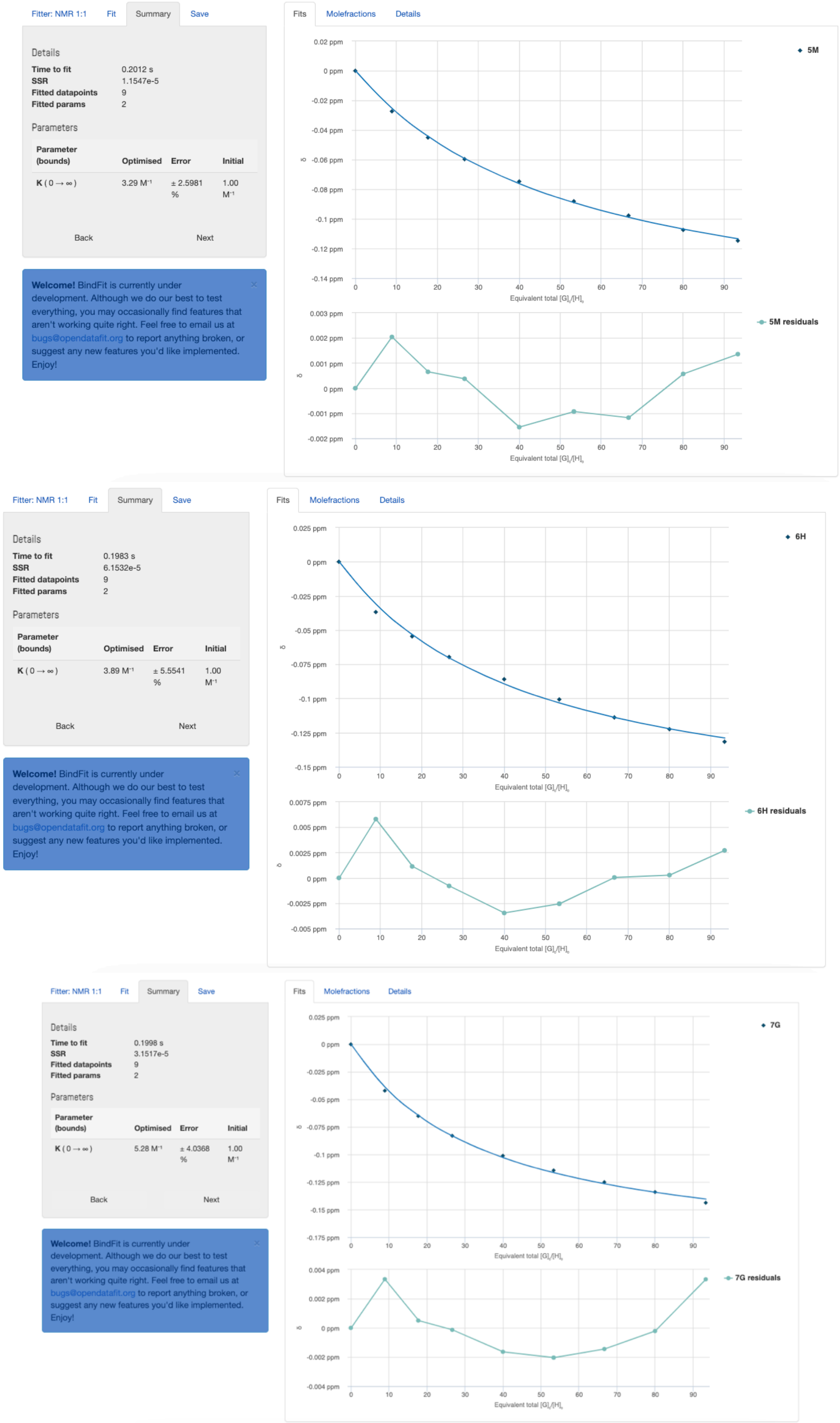

Figure S27: Fitting data of histidine mutant backbone amide N-H group chemical shifts (top: 5M, middle: 6H, bottom 7G) titrated with $NaClO_4$ at pH 2.3.

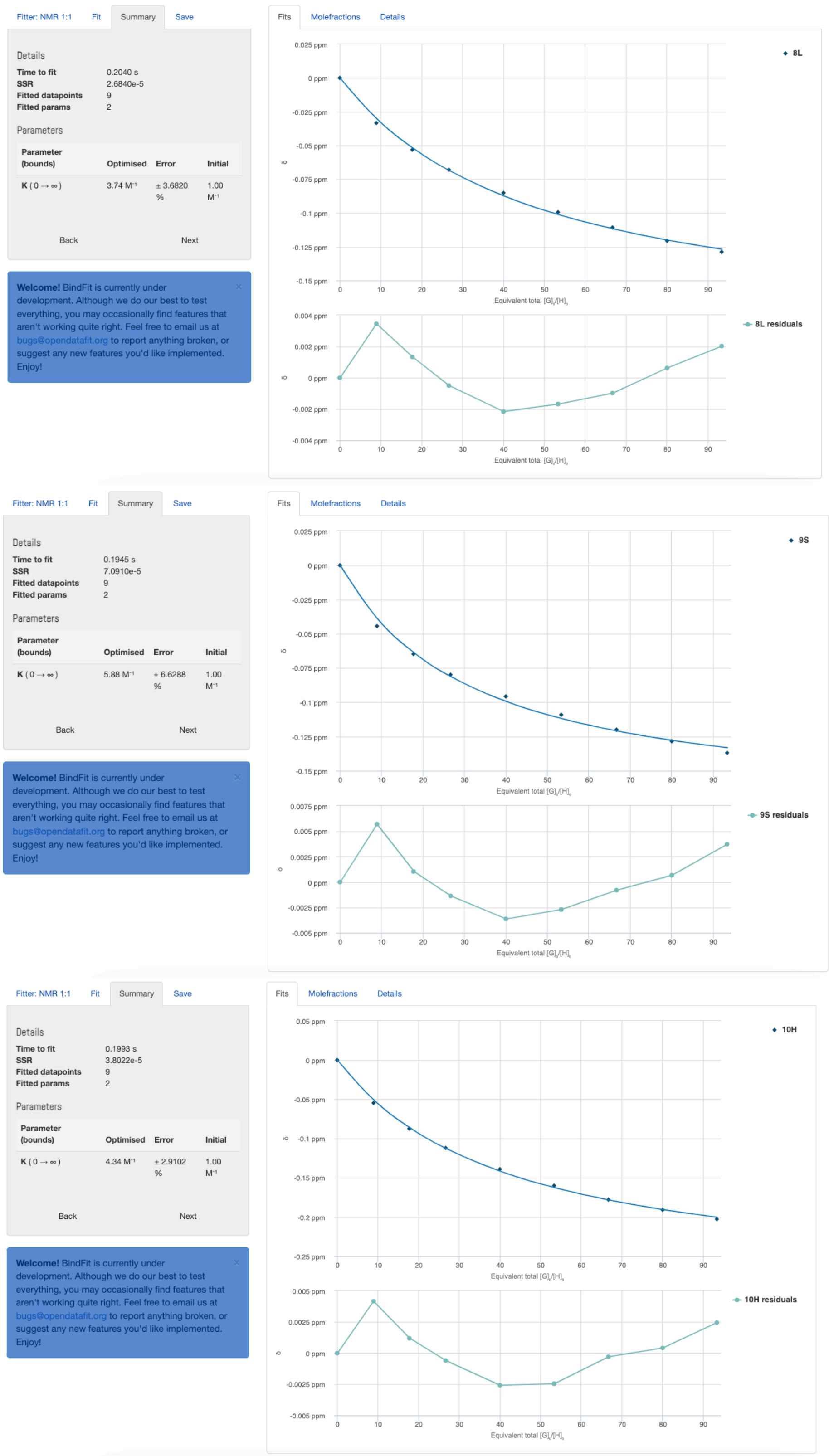

Figure S28: Fitting data of histidine mutant backbone amide group chemical shifts (top: 8L, middle: 9S, bottom 10H) titrated with $NaClO_4$ at pH 2.3.

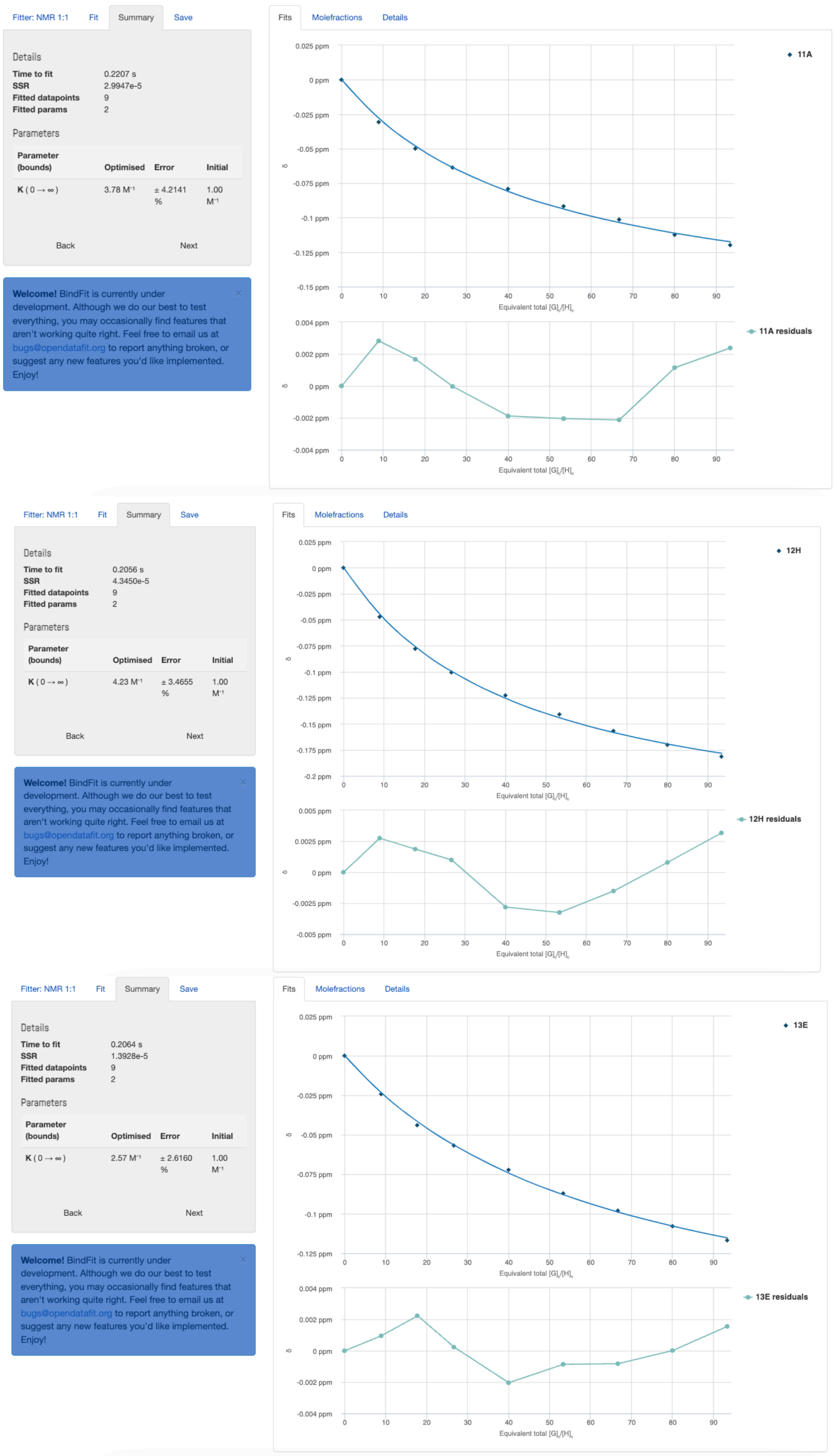

Figure S29: Fitting data of histidine mutant backbone amide N-H group chemical shifts (top: 11A, middle: 12H, bottom 13E) titrated with $NaClO_4$ at pH 2.3.

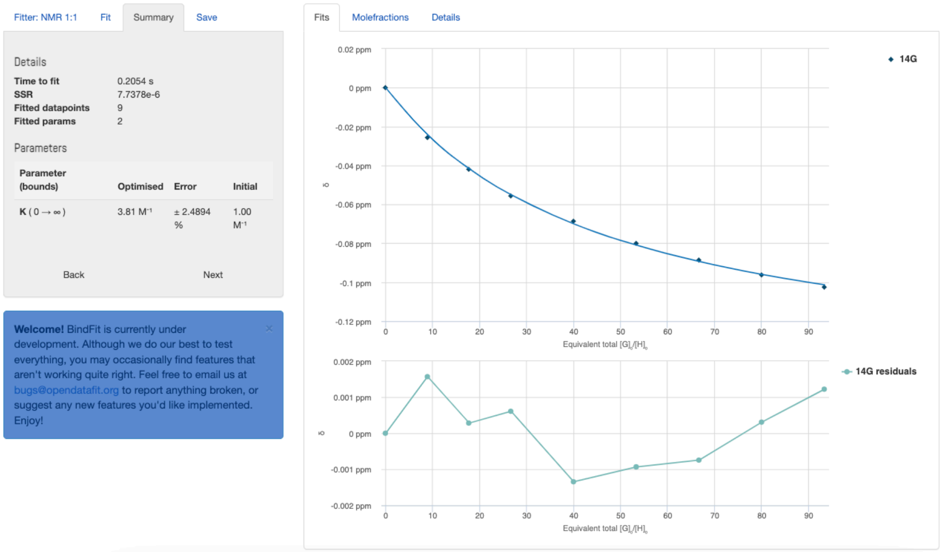

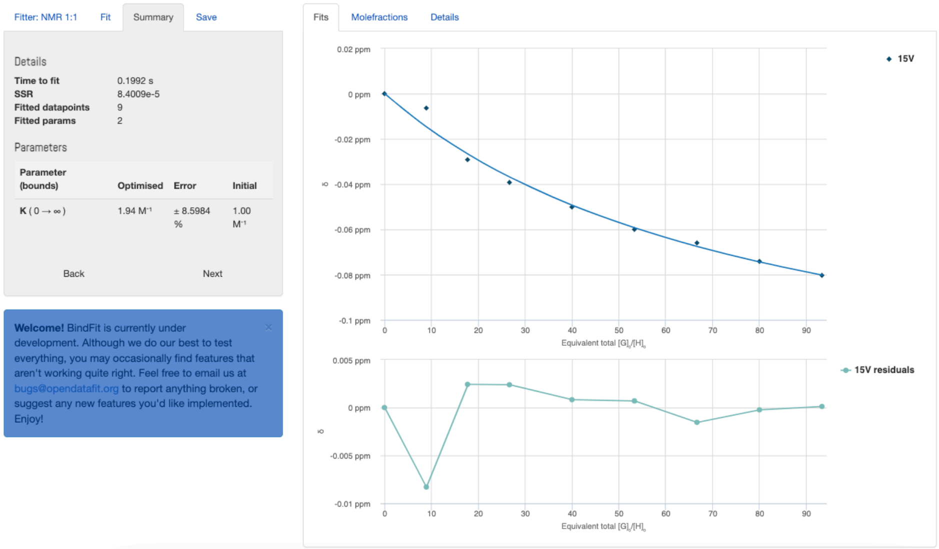

Figure S30: Fitting data of histidine mutant backbone amide N-H group chemical shifts (top: 14G, bottom: 15V) titrated with $NaClO_4$ at pH 2.3.

Table S8: Binding constant of histidine mutant α-SynH_{15} with 93 equiv. of $NaClO_4$ at 25°C and pH at 2.3.

	**2D**	**3V**	**4F**	**5M**	**6H**	**7G**	**8L**
K K_a	4	3	3	3	4	5	4
**Error (%)**	6	1	3	3	6	4	4
	**9S**	**10H**	**11A**	**12H**	**13E**	**14G**	**15V**
K_a	6	4	4	4	3	4	2
**Error (%)**	6	3	4	4	4	2	9

3.3 VT NMR experiments of peptides with/without salts

Chemical shifts for the wild-type α-Syn_{15} were determined as a function of temperature from 283 K to 313 K (5 K increments). These studies were carried out in the absence of salt and in the presence of NaCl and $NaClO_4$. Each signal shift was verified by ^1H-^{15}N HSQC NMR. Shown below is the shift data in tabular form, plots of shift versus temperature, and the tabulated summary of the linear fitting of the plot data.

1) Wild type α-Syn_{15} peptide

Table S9: Δδ of wild-type α-Syn_{15} with no salts from 283 K to 313 K at pH 5.2.

T(K) /Δδ(ppm)	2D	3V	4F	5M	6K	7G	8L
313	0.059	0.049	0.047	0.068	0.051	0.054	0.054
308	0.040	0.039	0.022	0.043	0.031	0.034	0.039
303	0.022	0.020	0.014	0.014	0.007	0.012	0.013
298	0.000	0.000	0.000	0.000	0.000	0.000	0.000
293	-0.025	-0.011	-0.013	-0.029	-0.019	-0.026	-0.022
288	-0.052	-0.024	-0.029	-0.057	-0.042	-0.049	-0.049
283	-0.072	-0.041	-0.051	-0.084	-0.059	-0.071	-0.071
**T(K) /Δδ(ppm)**	**9S**	**10K**	**11A**	**12K**	**13E**	**14G**	**15V**
313	0.051	0.035	0.044	0.028	0.049	0.052	0.050
308	0.026	0.016	0.029	0.023	0.029	0.032	0.020
303	0.011	0.000	0.004	0.006	0.009	0.010	0.010
298	0.000	0.000	0.000	0.000	0.000	0.000	0.000
293	-0.029	-0.026	-0.030	-0.016	-0.030	-0.019	-0.039
288	-0.052	-0.046	-0.046	-0.026	-0.047	-0.049	-0.058
283	-0.068	-0.056	-0.065	-0.032	-0.066	-0.064	-0.063

Table S10: Δδ of wild-type α-Syn_{15} with 50 equiv. NaCl from 283 K to 313 K at pH 5.2.

T(K) /Δδ(ppm)	2D	3V	4F	5M	6K	7G	8L
313	0.065	0.044	0.053	0.071	0.053	0.061	0.064
308	0.043	0.031	0.038	0.048	0.034	0.040	0.041
303	0.022	0.017	0.024	0.024	0.018	0.021	0.023
298	0.000	0.000	0.000	0.000	0.000	0.000	0.000
293	-0.023	-0.013	-0.015	-0.023	-0.017	-0.020	-0.023
288	-0.043	-0.028	-0.033	-0.049	-0.035	-0.042	-0.042
283	-0.060	-0.034	-0.043	-0.064	-0.047	-0.056	-0.057
**T(K) /Δδ(ppm)**	**9S**	**10K**	**11A**	**12K**	**13E**	**14G**	**15V**
313	0.061	0.050	0.054	0.036	0.057	0.055	0.067
308	0.039	0.035	0.036	0.023	0.036	0.039	0.043
303	0.022	0.021	0.019	0.011	0.019	0.020	0.022
298	0.000	0.000	0.000	0.000	0.000	0.000	0.000
293	-0.020	-0.020	-0.018	-0.012	-0.018	-0.018	-0.013
288	-0.040	-0.035	-0.034	-0.024	-0.037	-0.037	-0.028
283	-0.054	-0.044	-0.047	-0.031	-0.049	-0.049	-0.034

Table S11: Δδ of wild-type α-Syn_{15} with 50 equiv. $NaClO_4$ from 283 K to 313 K at pH 5.2.

T(K) /Δδ(ppm)	2D	3V	4F	5M	6K	7G	8L
313	0.065	0.042	0.054	0.070	0.060	0.071	0.078
308	0.044	0.026	0.043	0.049	0.038	0.046	0.052
303	0.019	0.009	0.022	0.033	0.019	0.024	0.030
298	0.000	0.000	0.000	0.000	0.000	0.000	0.000
293	-0.028	-0.022	-0.010	-0.029	-0.012	-0.021	-0.016
288	-0.053	-0.035	-0.032	-0.048	-0.034	-0.048	-0.047
283	-0.083	-0.054	-0.049	-0.078	-0.056	-0.075	-0.069
**T(K) /Δδ(ppm)**	**9S**	**10K**	**11A**	**12K**	**13E**	**14G**	**15V**
313	0.070	0.061	0.057	0.050	0.062	0.057	0.071
308	0.040	0.038	0.037	0.036	0.041	0.041	0.050
303	0.022	0.026	0.011	0.019	0.020	0.016	0.020
298	0.000	0.000	0.000	0.000	0.000	0.000	0.000
293	-0.025	-0.025	-0.017	-0.010	-0.028	-0.032	-0.022
288	-0.050	-0.036	-0.040	-0.018	-0.036	-0.042	-0.035
283	-0.073	-0.057	-0.072	-0.040	-0.061	-0.070	-0.051

1a): Δδ of amide N–H groups in wild type α-Syn$_{15}$ in the absence of salt.

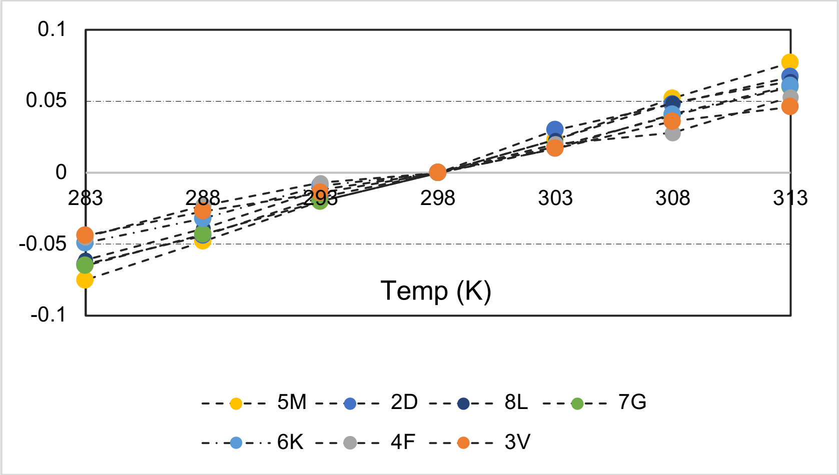

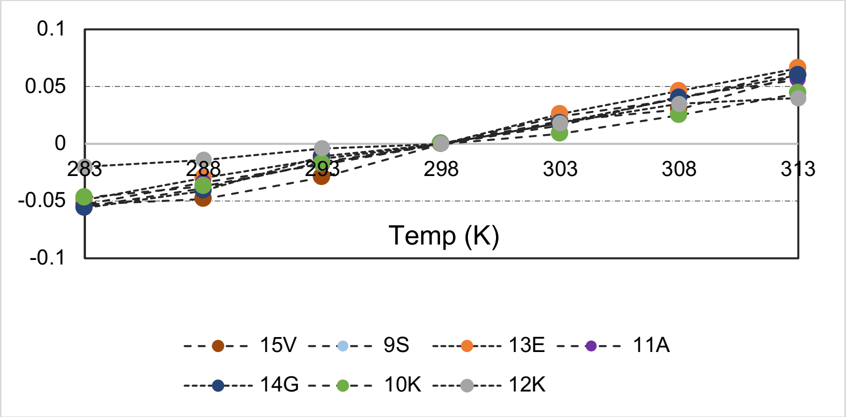

Figure S31: Δδ of backbone amide N-H groups of wild-type α-Syn$_{15}$ without salt at pH 5.2; Top 2D-8L, bottom 9S-15V.

Table S12: Linear-fitting data of Δδ with temperature without salt.

AA	Slope	y-intercept	R^2
2D	4.46E-03	-1.332	0.996
3V	3.05E-03	-0.904	0.994
4F	3.02E-03	-0.902	0.985
5M	4.99E-03	-1.494	0.996
6K	3.59E-03	-1.073	0.991
7G	4.14E-03	-1.239	0.995
8L	4.19E-03	-1.253	0.993
9S	3.95E-03	-1.186	0.990
10K	3.02E-03	-0.911	0.974
11A	3.65E-03	-1.097	0.987
12K	2.14E-03	-0.641	0.985
13E	3.83E-03	-1.149	0.992
14G	3.85E-03	-1.153	0.990
15V	3.89E-03	-1.169	0.966

1b): Δδ of amide N–H groups in wild type α-Syn$_{15}$ in the presence of NaCl.

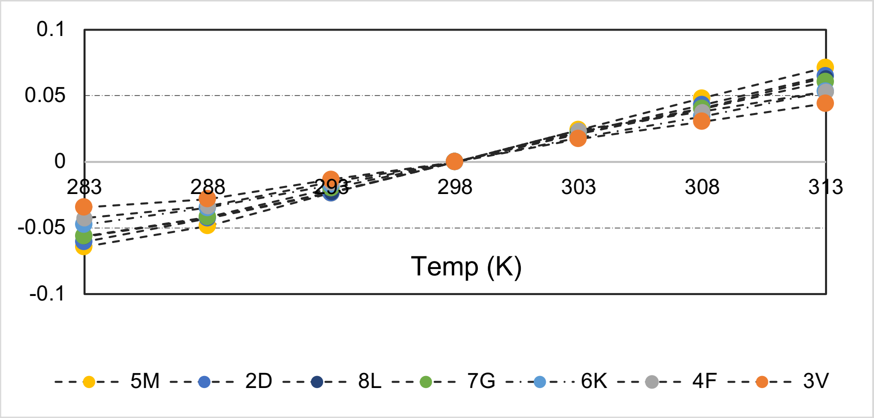

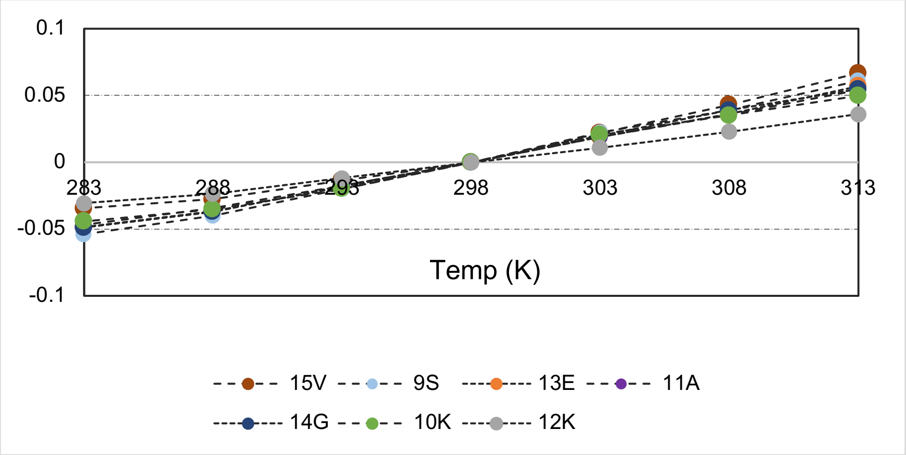

Figure S32: Δδ of backbone amide N-H with wild-type α-Syn$_{15}$ with NaCl at pH 5.2. Top, 2D-8L; bottom, 9S-15V.

Table S13: Linear-fitting data of Δδ with temperature in the presence of NaCl.

AA	Slope	y-intercept	R^2
2D	4.24E-03	-1.262	0.999
3V	2.74E-03	-0.816	0.992
4F	3.35E-03	-0.995	0.992
5M	4.63E-03	-1.378	0.998
6K	3.40E-03	-1.012	0.998
7G	3.97E-03	-1.183	0.999
8L	4.09E-03	-1.219	0.997
9S	3.89E-03	-1.157	0.998
10K	3.31E-03	-0.984	0.993
11A	3.43E-03	-1.020	0.998
12K	2.25E-03	-0.671	0.996
13E	3.57E-03	-1.062	0.997
14G	3.58E-03	-1.064	0.997
15V	3.43E-03	-1.015	0.973

1c): Δδ of amide N–H groups in wild type α-Syn$_{15}$ in the presence of $NaClO_4$.

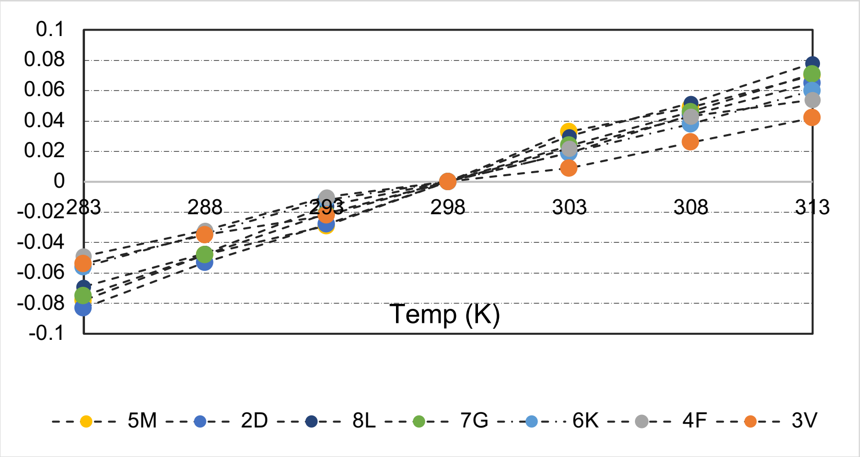

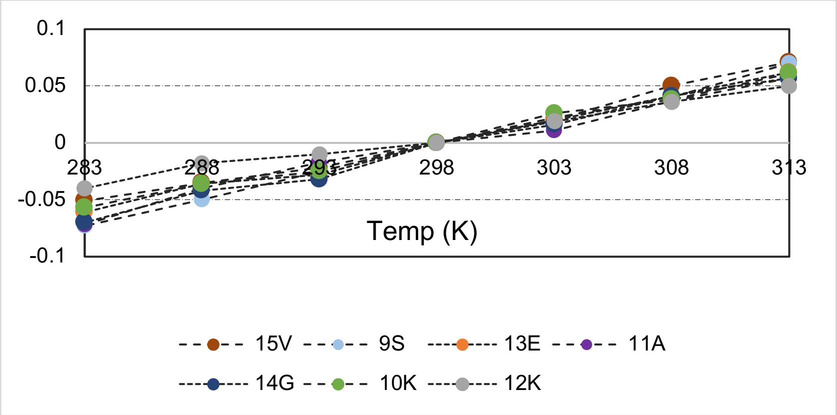

Figure S33: Δδ of backbone amide N-H with wild-type α-Syn$_{15}$ with $NaClO_4$ at pH 5.2. Top, 2D-8L; bottom, 9S-15V.

Table S14: Linear-fitting data of Δδ with temperature in the presence of $NaClO_4$.

AA	Slope	y-intercept	R^2
2D	4.89E-03	-1.463	0.996
3V	3.15E-03	-0.944	0.995
4F	3.51E-03	-1.041	0.994
5M	5.00E-03	-1.490	0.993
6K	3.74E-03	-1.111	0.996
7G	4.79E-03	-1.429	0.999
8L	4.89E-03	-1.454	0.997
9S	4.69E-03	-1.399	0.998
10K	3.95E-03	-1.176	0.993
11A	4.06E-03	-1.215	0.986
12K	2.91E-03	-0.861	0.988
13E	4.08E-03	-1.216	0.993
14G	4.25E-03	-1.271	0.992
15V	4.13E-03	-1.226	0.987

2) Triple histidine mutant α-Syn$_{15}$H peptide

Similar studies have been carried out to determine the chemical shifts for the triple histidine mutant α-Syn$_{15}$H as a function of temperature from 283 K to 313 K (5 K increments).

Table S15: Δδ of triple histidine mutant α-Syn$_{15}$H with no salts from 283 K to 313 K at pH 2.3.

T(K) /Δδ(ppm)	2D	3V	4F	5M	6H	7G	8L
313	0.068	0.04	0.053	0.062	0.071	0.067	0.069
308	0.044	0.024	0.037	0.041	0.052	0.046	0.043
303	0.023	0.008	0.021	0.026	0.031	0.02	0.022
298	0.000	0.000	0.000	0.000	0.000	0.000	0.000
293	-0.027	-0.016	-0.017	-0.017	-0.017	-0.025	-0.017
288	-0.049	-0.028	-0.036	-0.036	-0.036	-0.048	-0.044
283	-0.077	-0.048	-0.056	-0.052	-0.064	-0.068	-0.068
**T(K) /Δδ(ppm)**	**9S**	**10H**	**11A**	**12H**	**13E**	**14G**	**15V**
313	0.054	0.069	0.073	0.055	0.069	0.062	0.049
308	0.034	0.048	0.051	0.034	0.046	0.044	0.036
303	0.013	0.024	0.027	0.019	0.024	0.024	0.019
298	0.000	0.000	0.000	0.000	0.000	0.000	0.000
293	-0.022	-0.012	-0.022	-0.014	-0.018	-0.013	-0.01
288	-0.041	-0.04	-0.044	-0.036	-0.044	-0.037	-0.024
283	-0.062	-0.069	-0.072	-0.060	-0.056	-0.065	-0.036

Table S16: Δδ of triple histidine mutant α-Syn$_{15}$H with NaCl from 283 K to 313 K at pH 2.3.

T(K) /Δδ(ppm)	2D	3V	4F	5M	6H	7G	8L
313	0.072	0.04	0.053	0.06	0.065	0.073	0.06
308	0.048	0.024	0.037	0.044	0.044	0.056	0.044
303	0.024	0.008	0.024	0.024	0.025	0.028	0.024
298	0.000	0.000	0.000	0.000	0.000	0.000	0.000
293	-0.029	-0.016	-0.016	-0.012	-0.028	-0.02	-0.02
288	-0.049	-0.028	-0.032	-0.032	-0.044	-0.044	-0.048
283	-0.077	-0.044	-0.052	-0.052	-0.072	-0.076	-0.072
**T(K) /Δδ(ppm)**	**9S**	**10H**	**11A**	**12H**	**13E**	**14G**	**15V**
313	0.052	0.064	0.069	0.052	0.056	0.052	0.045
308	0.04	0.04	0.045	0.036	0.044	0.04	0.033
303	0.016	0.027	0.029	0.02	0.02	0.016	0.021
298	0.000	0.000	0.000	0.000	0.000	0.000	0.000
293	-0.028	-0.024	-0.02	-0.016	-0.024	-0.029	-0.004
288	-0.045	-0.049	-0.048	-0.032	-0.044	-0.049	-0.016
283	-0.073	-0.061	-0.072	-0.048	-0.068	-0.065	-0.032

Table S17: Δδ of triple histidine mutant α-Syn$_{15}$H with $NaClO_4$ from 283 K to 313 K at pH 2.3.

T(K) /Δδ(ppm)	2D	3V	4F	5M	6H	7G	8L
313	0.076	0.045	0.056	0.063	0.076	0.079	0.073
308	0.051	0.028	0.039	0.041	0.052	0.051	0.048
303	0.027	0.016	0.013	0.02	0.027	0.027	0.024
298	0.000	0.000	0.000	0.000	0.000	0.000	0.000
293	-0.032	-0.018	-0.025	-0.02	-0.029	-0.026	-0.033
288	-0.048	-0.026	-0.042	-0.047	-0.051	-0.056	-0.057
283	-0.093	-0.049	-0.063	-0.072	-0.083	-0.087	-0.083
**T(K) /Δδ(ppm)**	**9S**	**10H**	**11A**	**12H**	**13E**	**14G**	**15V**
313	0.075	0.079	0.073	0.064	0.065	0.062	0.044
308	0.047	0.056	0.051	0.042	0.046	0.04	0.031
303	0.024	0.033	0.026	0.021	0.018	0.017	0.014
298	0.000	0.000	0.000	0.000	0.000	0.000	0.000
293	-0.025	-0.031	-0.025	-0.024	-0.029	-0.025	-0.02
288	-0.046	-0.053	-0.053	-0.04	-0.05	-0.041	-0.037
283	-0.077	-0.095	-0.082	-0.074	-0.08	-0.076	-0.05

2a): Δδ of amide N–H groups in triple histidine-mutant α-Syn$_{15}$H in the absence of salt.

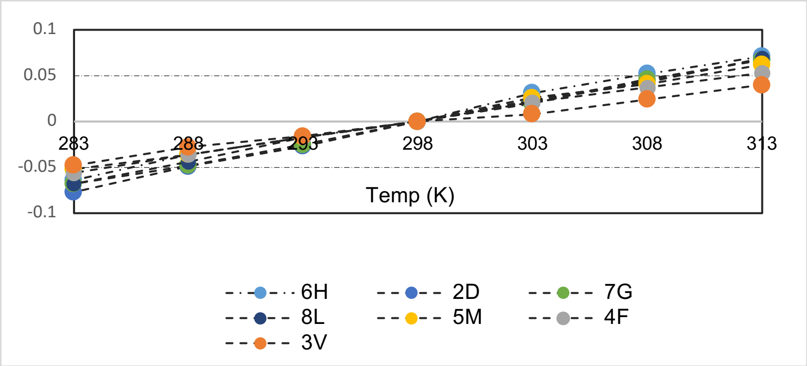

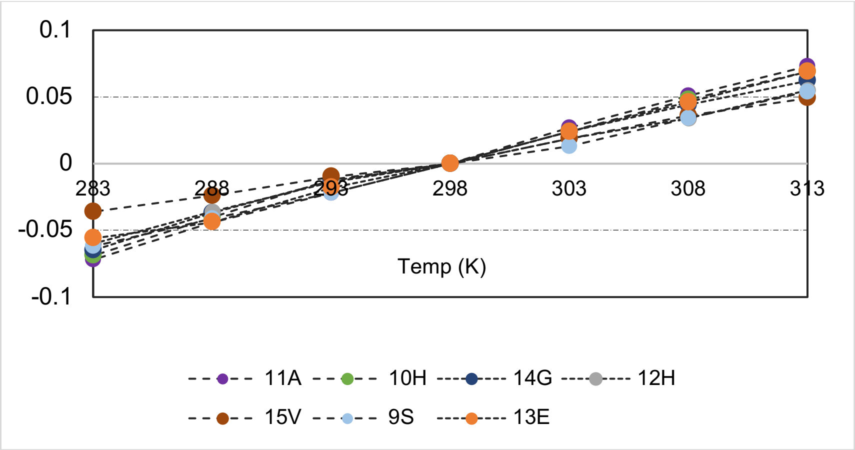

Figure S34: Δδ of backbone amide N-H of histidine-mutant α-Syn$_{15}$H without salt at pH 2.3. Top, 2D-8L; bottom, 9S-15V.

Table S18: Linear-fitting data of Δδ with temperature without salt.

AA	Slope	Intercept	R^2
2D	4.79E-03	-1.431	0.999
3V	2.80E-03	-0.837	0.993
4F	3.65E-03	-1.087	0.999
5M	3.85E-03	-1.144	0.997
6H	4.49E-03	-1.334	0.996
7G	4.56E-03	-1.359	0.999
8L	4.46E-03	-1.328	0.998
9S	3.81E-03	-1.138	0.998
10H	4.47E-03	-1.330	0.993
11A	4.81E-03	-1.433	0.999
12H	3.70E-03	-1.103	0.995
13E	4.26E-03	-1.268	0.996
14G	4.14E-03	-1.232	0.994
15V	2.89E-03	-0.855	0.994

2a): Δδ of amide N–H groups in triple histidine-mutant α-Syn_{15}H in the presence of NaCl.

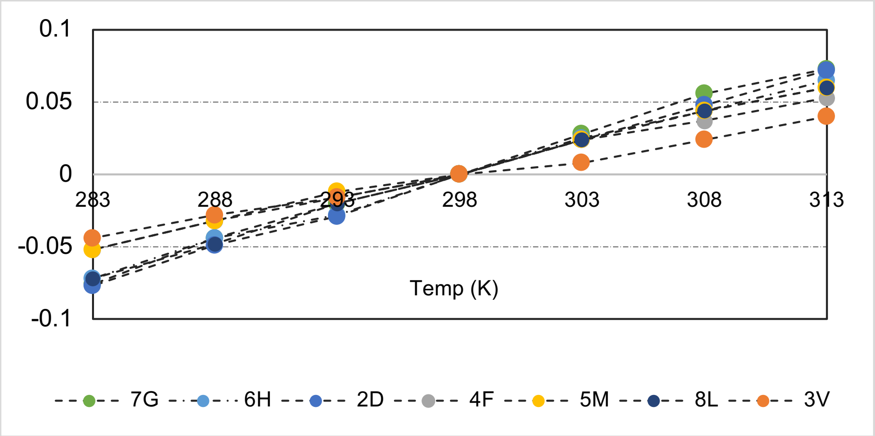

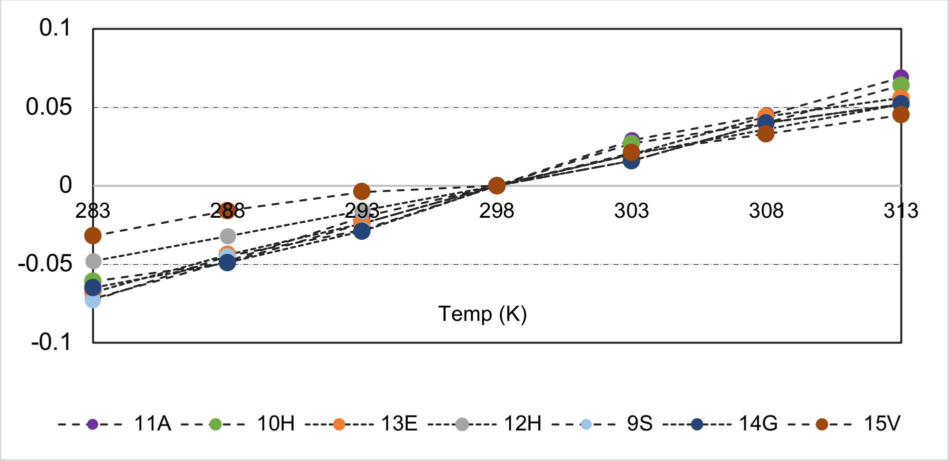

Figure S35: Δδ of backbone amide N-H of histidine-mutant α-Syn_{15}H with NaCl at pH 2.3. Top, 2D-8L; bottom, 9S-15V.

Table S19: Linear-fitting data of Δδ with temperature with NaCl.

AA	Slope	Intercept	R^2
2D	4.96E-03	-1.479	0.999
3V	2.71E-03	-0.811	0.996
4F	3.52E-03	-1.047	0.997
5M	3.74E-03	-1.111	0.997
6H	4.57E-03	-1.364	0.997
7G	4.96E-03	-1.477	0.996
8L	4.46E-03	-1.330	0.995
9S	4.21E-03	-1.259	0.992
10H	4.31E-03	-1.286	0.993
11A	4.70E-03	-1.400	0.997
12H	3.37E-03	-1.003	0.999
13E	4.23E-03	-1.262	0.996
14G	4.10E-03	-1.227	0.993
15V	2.53E-03	-0.747	0.987

2a): Δδ of amide N–H groups in triple histidine-mutant α-Syn$_{15}$H in the presence of $NaClO_4$

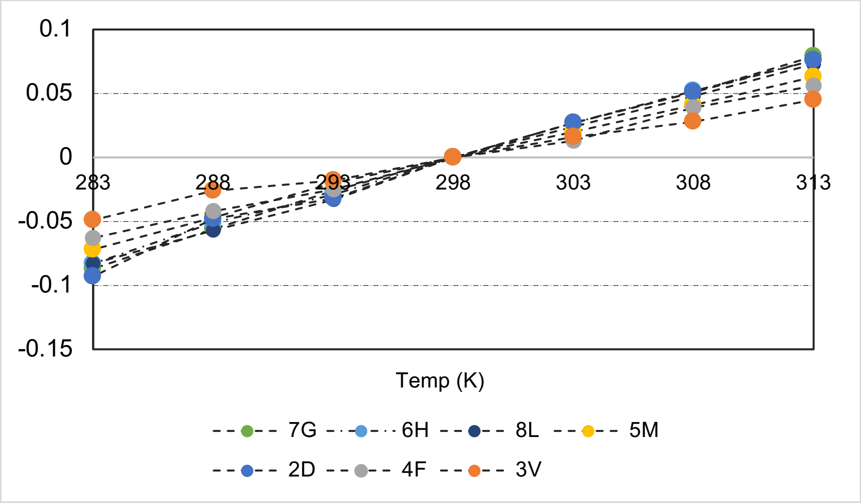

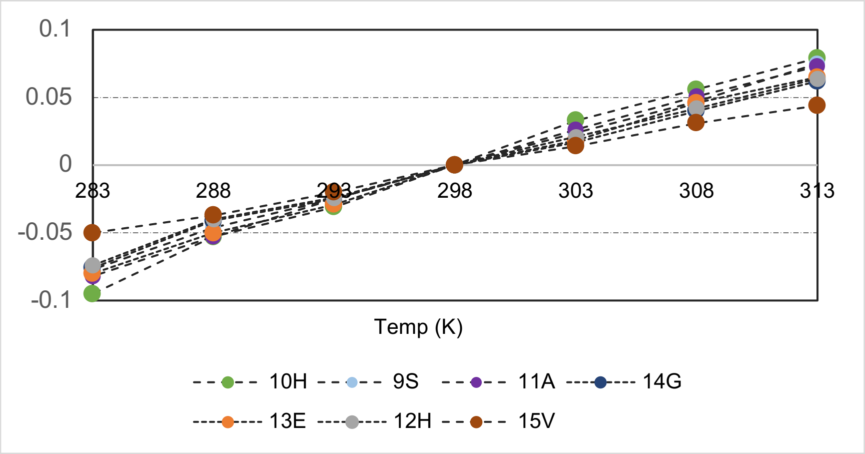

Figure S36: Δδ of backbone amide N-H of histidine-mutant α-Syn$_{15}$ with $NaClO_4$ at pH 2.3 up (2D-8L) down: (9S-15V)

Table S20: Linear-fitting data of Δδ with temperature with $NaClO_4$.

AA	Slope	Intercept	R^2
2D	5.46E-03	-1.629	0.991
3V	3.03E-03	-0.903	0.993
4F	3.98E-03	-1.189	0.997
5M	4.44E-03	-1.324	0.997
6H	5.28E-03	-1.574	0.999
7G	5.46E-03	-1.630	0.999
8L	5.25E-03	-1.569	0.998
9S	4.94E-03	-1.471	0.998
10H	5.74E-03	-1.713	0.993
11A	5.17E-03	-1.543	0.998
12H	4.45E-03	-1.328	0.995
13E	4.81E-03	-1.439	0.997
14G	4.41E-03	-1.319	0.993
15V	3.23E-03	-0.965	0.997

3.4 Peptide conformations define from *J*-coupling constants

To investigate if anion affinity affected the backbone conformation, TROSY NMR was used to measure the coupling constant of each N–H signal to determine the φ angle between the N–H and C_α–H of each residue.[1] The histidine mutant α-Syn_{15}H was selected because of its excellent signal anisotropy. Three distinct conditions were selected: no salt, and 90 equiv. NaCl or $NaClO_4$ (all data collected at 25°C). The maximum error of J-coupling constant is ± 0.005 ppm.

According to the relationship between backbone φ angles and coupling constant:[2]

$$J(\varphi) = 7.97 \cos^2 (\varphi-60) -1.26 \cos (\varphi-60) + 0.63$$

The average φ angles were calculated from its related coupling constants. Coupling constants (J) and the calculated φ angles are shown below.

Table S21: *J*-coupling constant of histidine mutant with 90 equiv. NaCl or $NaClO_4$.

	Coupling constant *J* (Hz)						
	**2D**	**3V**	**4F**	**5M**	**6H**	**7G**	**8L**
No salt	7.23	6.86	7.42	7.70	6.65	6.30	7.14
NaCl	7.43	7.42	7.42	7.91	6.93	6.72	7.00
$NaClO_4$	7.39	8.12	7.35	7.42	6.37	6.79	7.91
	**9S**	**10H**	**11A**	**12H**	**13E**	**14G**	**15V**
No salt	6.76	7.79	5.67	7.35	7.17	6.17	8.23
NaCl	6.65	7.42	5.67	7.42	7.07	6.51	8.19
$NaClO_4$	6.79	7.70	5.60	7.70	6.37	6.09	8.75

Table S22: Backbone φ dihedral angle of histidine mutant with no salt, 90 equiv. NaCl or $NaClO_4$.

	φ dihedral angle (°)						
	**2D**	**3V**	**4F**	**5M**	**6H**	**7G**	**8L**
No salt	-146.6	-144.0	-147.9	-150.0	-142.5	-140.2	-145.9
NaCl	-148.0	-147.9	-147.9	-151.6	-144.4	-143.0	-144.9
$NaClO_4$	-147.7	-153.3	-147.4	-147.9	-140.6	-143.5	-151.6
	**9S**	**10H**	**11A**	**12H**	**13E**	**14G**	**15V**
No salt	-143.3	-150.7	-136.1	-147.4	-146.1	-139.3	-154.2
NaCl	-142.5	-147.9	-136.1	-147.9	-145.4	-141.6	-153.9
$NaClO_4$	-143.5	-150.0	-135.6	-150.0	-140.6	-138.8	-159.0

4. Aggregation studies

4.1 Aggregation of arginine mutant with salts

In the aggregation studies, the concentration of the triple arginine mutant (α-SynR) was 2 mM, pD 5.2 in 50 mM sodium acetate buffer. One equiv. of the external reference sodium ethyl sulfate was added to each (as 2.6 μL of a 600 mM mother solution in D_2O). The ratio of peptide to reference was calculated based on the integral of peptide methyl group from 3V, 8L, and 15V.

a) Aggregation induced by $NaClO_4$.

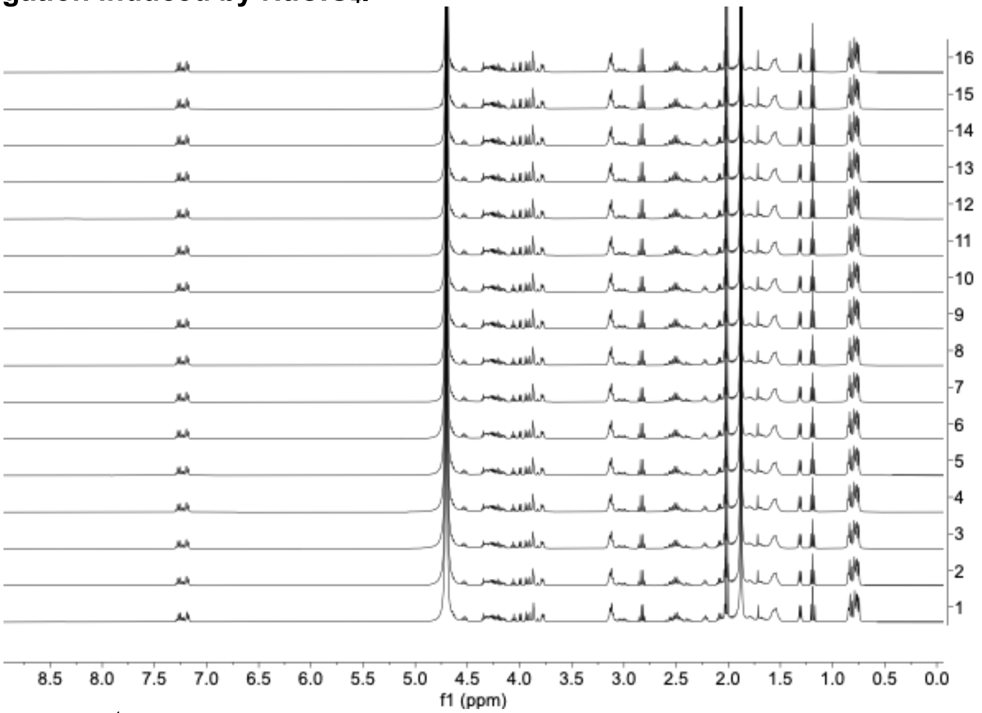

Figure S37: ^{1}H NMR spectra over time of arginine mutant(α-SynR) with 10 equiv. $NaClO_4$ at pD 5.2.

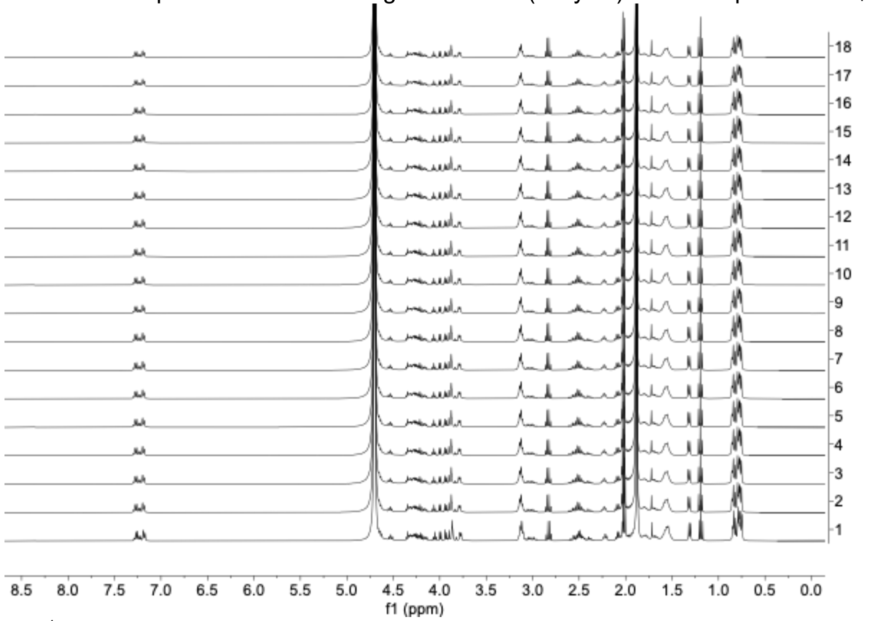

Figure S38: ^{1}H NMR spectra over time of arginine mutant (α-SynR) with 20 equiv. $NaClO_4$ at pD 5.2.

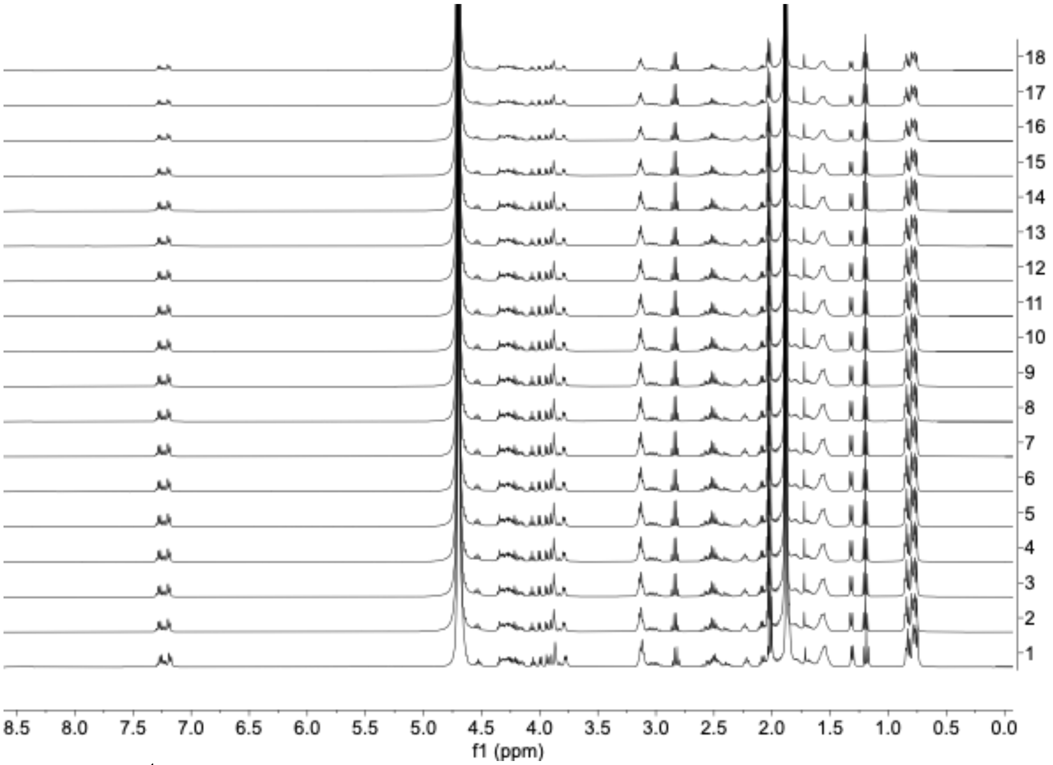

Figure S39: ^1H NMR spectra over time of arginine mutant (α-SynR) with 30 equiv. $NaClO_4$ at pD 5.2.

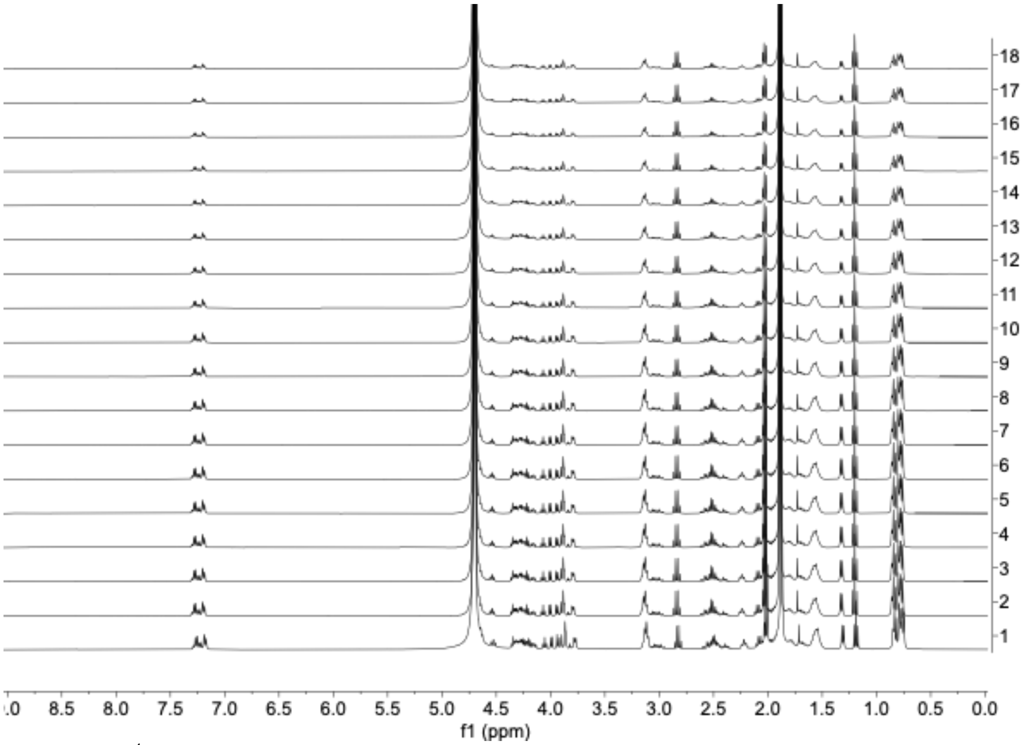

Figure S40: ^1H NMR spectra over time of arginine mutant (α-SynR) with 40 equiv. $NaClO_4$ at pD 5.2.

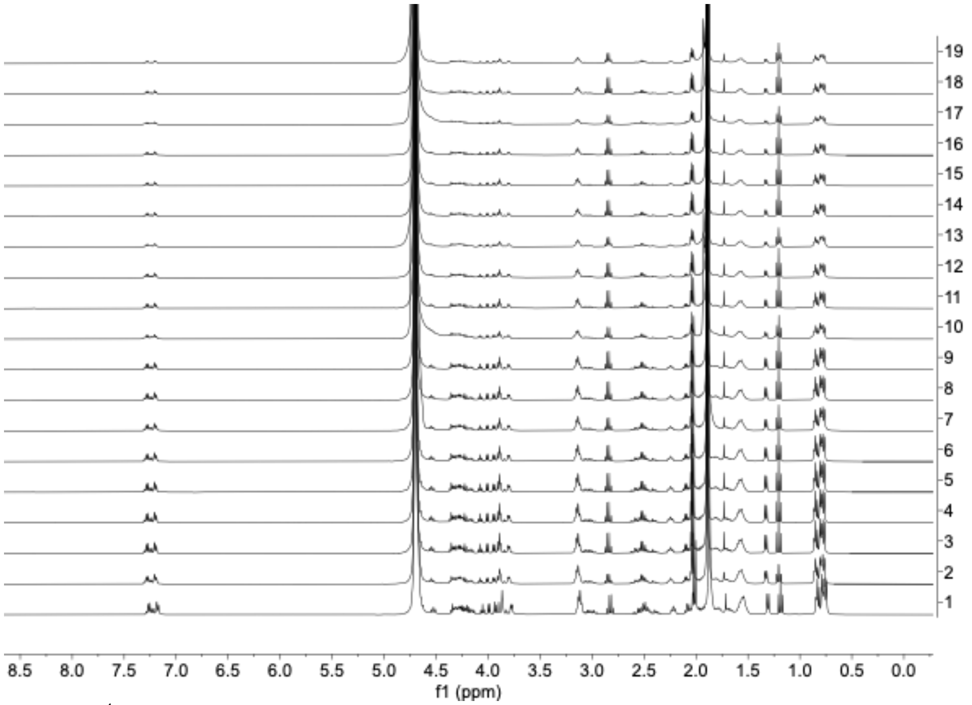

Figure S41: ^{1}H NMR spectra over time of arginine mutant (α-SynR) with 60 equiv. $NaClO_4$ at pD 5.2.

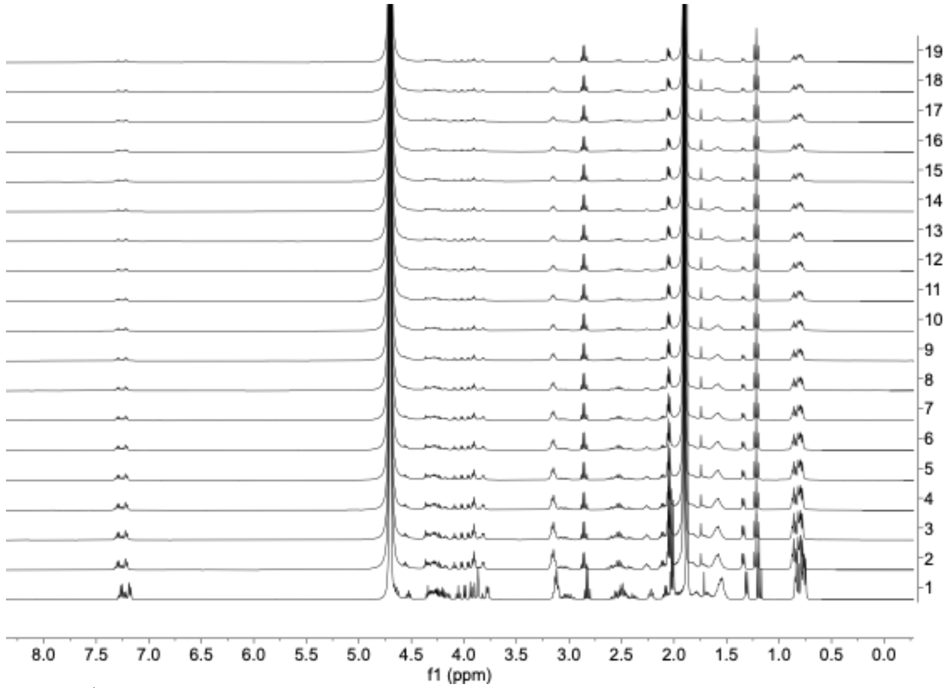

Figure S42: ^{1}H NMR spectra over time of arginine mutant (α-SynR) with 100 equiv. $NaClO_4$ at pD 5.2.

Table S23: Arginine triple mutant peptide (α-SynR) aggregation data with varying concentrations of $NaClO_4$.

Time (h)	Mole fraction lost (10 eq.)	error	Mole fraction lost (20 eq.)	error	Mole fraction lost (30 eq.)	error
0	0.00	-	0.00		0.00	
1	0.00	-	0.03	0.013	0.02	0.003
2	0.00	-	0.04	0.019	0.04	0.009
3	0.01	-	0.05	0.011	0.05	0.001
4	0.01	-	0.05	0.012	0.06	0.008
5	0.01	-	0.06	0.015	0.06	0.005
6	0.01	-	0.06	0.014	0.07	0.007
7	0.01	-	0.07	0.018	0.08	0.008
8	0.01	-	0.08	0.016	0.10	0.009
9	0.01	-	0.09	0.016	0.12	0.001
10	0.02	-	0.11	0.027	0.15	0.010
11	0.01	-	0.12	0.022	0.19	0.022
12	0.02	-	0.14	0.030	0.28	0.037
14	0.02	-	0.18	0.037	0.43	0.004
16	0.02	-	0.24	0.055	0.48	0.028
20	0.01	-	0.27	0.021	0.50	0.023
22	0.01	-	0.31	0.041	0.52	0.021
**Time (h)**	**40 equiv.**	**error**	**60 eq**	**error**	**100 eq**	**error**
0	0.00	0.000	0.00	0.000	0.00	0.001
1	0.01	0.000	0.05	0.014	0.08	0.000
2	0.03	0.012	0.08	0.017	0.19	0.005
3	0.05	0.007	0.11	0.020	0.30	0.006
4	0.06	0.005	0.14	0.018	0.38	0.003
5	0.10	0.005	0.19	0.021	0.46	0.004
6	0.13	0.010	0.23	0.013	0.51	0.005
7	0.17	0.004	0.32	0.009	0.56	0.010
8	0.24	0.014	0.41	0.039	0.61	0.004
9	0.31	0.013	0.48	0.043	0.63	0.005
10	0.39	0.022	0.55	0.033	0.65	0.004
11	0.45	0.014	0.60	0.035	0.67	0.003
12	0.49	0.008	0.62	0.029	0.68	0.005
14	0.54	0.008	0.64	0.017	0.68	0.007
16	0.56	0.025	0.65	0.014	0.69	0.005
20	0.58	0.032	0.66	0.002	0.70	0.001
22	0.59	0.023	0.66	0.008	0.71	0.004

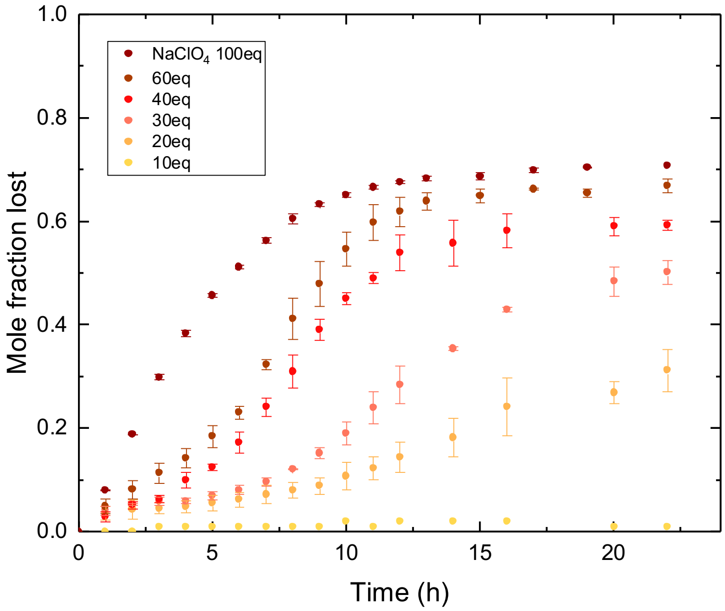

Figure S43: Mole fraction of triple arginine mutant (α-SynR) lost in the presence of varying concentrations of $NaClO_4$ at pD 5.2.

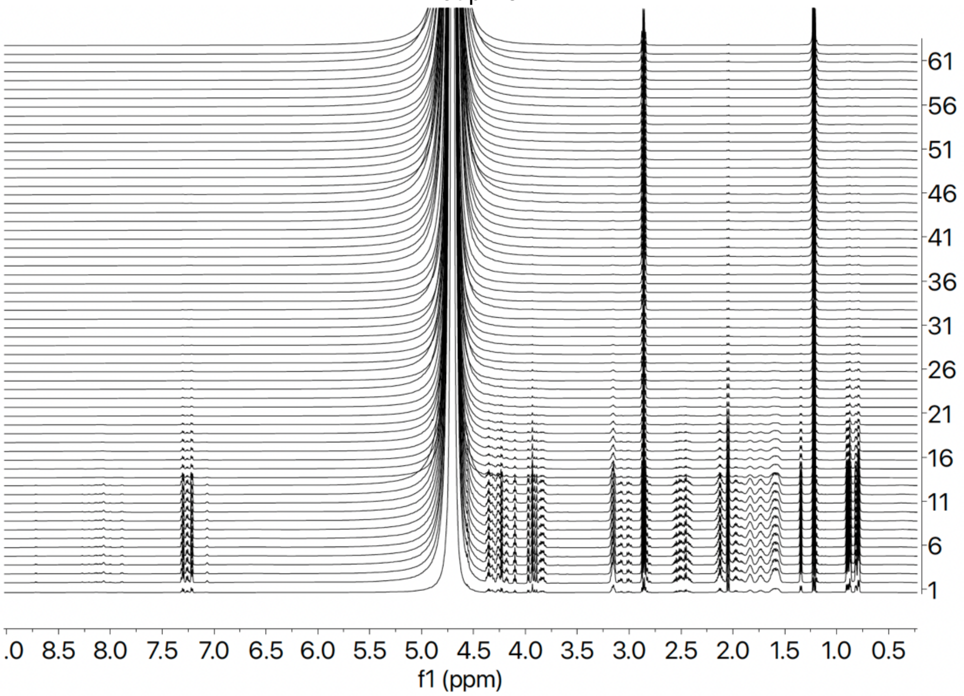

Figure S44: ^1H NMR spectra over time of arginine mutant (α-SynR) with 100 equiv. $NaClO_4$ at pD 2.3.

Table S24: Arginine triple mutant peptide (α-SynR) aggregation data with 100 equiv. $NaClO_4$ at pD 2.3

Time (h)	Mole fraction lost (100 eq.)	error
0.0	0.00	0.000
0.5	0.00	0.001
1.1	0.00	0.001
1.2	0.01	0.007
1.4	0.02	0.002
1.5	0.04	0.005
1.7	0.08	0.007
1.8	0.12	0.008
1.9	0.16	0.003
2.0	0.20	0.012
2.1	0.24	0.010
2.1	0.28	0.016
2.2	0.33	0.018
2.3	0.39	0.019
2.4	0.46	0.019
2.5	0.52	0.019
2.5	0.59	0.016
2.6	0.65	0.012
2.7	0.71	0.006
2.8	0.75	0.025
2.9	0.78	0.009
2.9	0.79	0.007
3.0	0.80	0.003
3.2	0.82	0.002
3.3	0.82	0.005
3.6	0.84	0.006
3.9	0.84	0.007

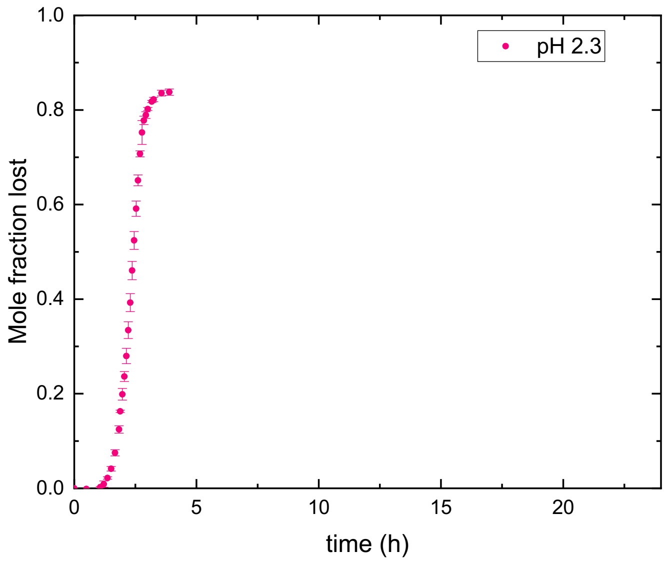

Figure S45: Mole fraction of triple arginine mutant (α-SynR) lost in the presence of 100 equiv. $NaClO_4$ at pD 2.3.

b) Aggregation induced by NaI

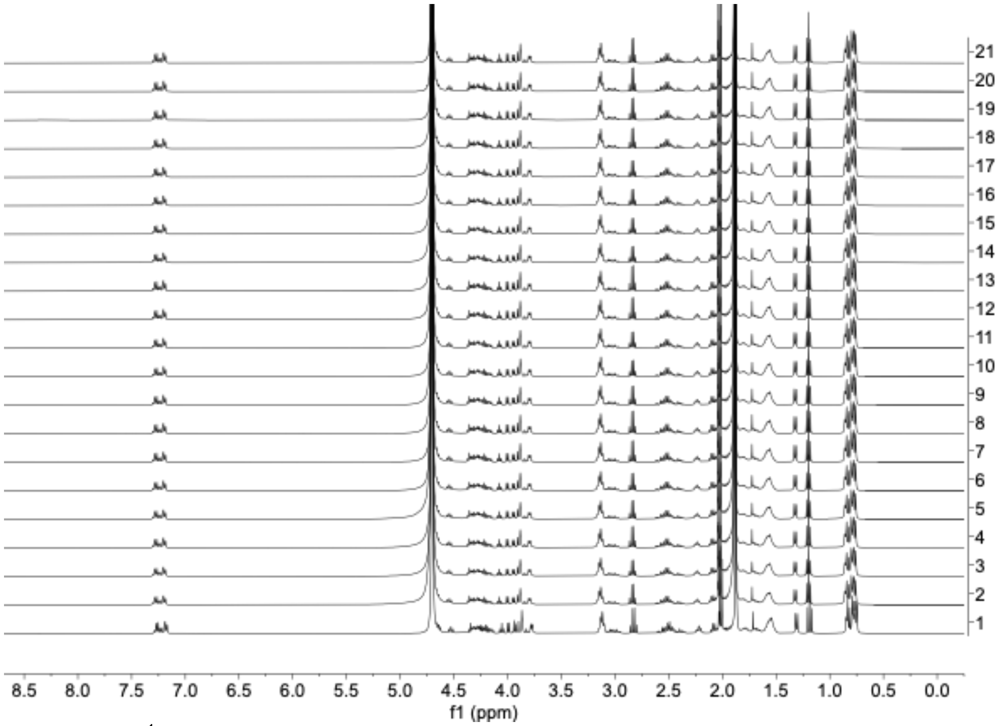

Figure S46: ^{1}H NMR spectra over time of arginine mutant (α-SynR) with 30 equiv. NaI at pD 5.2.

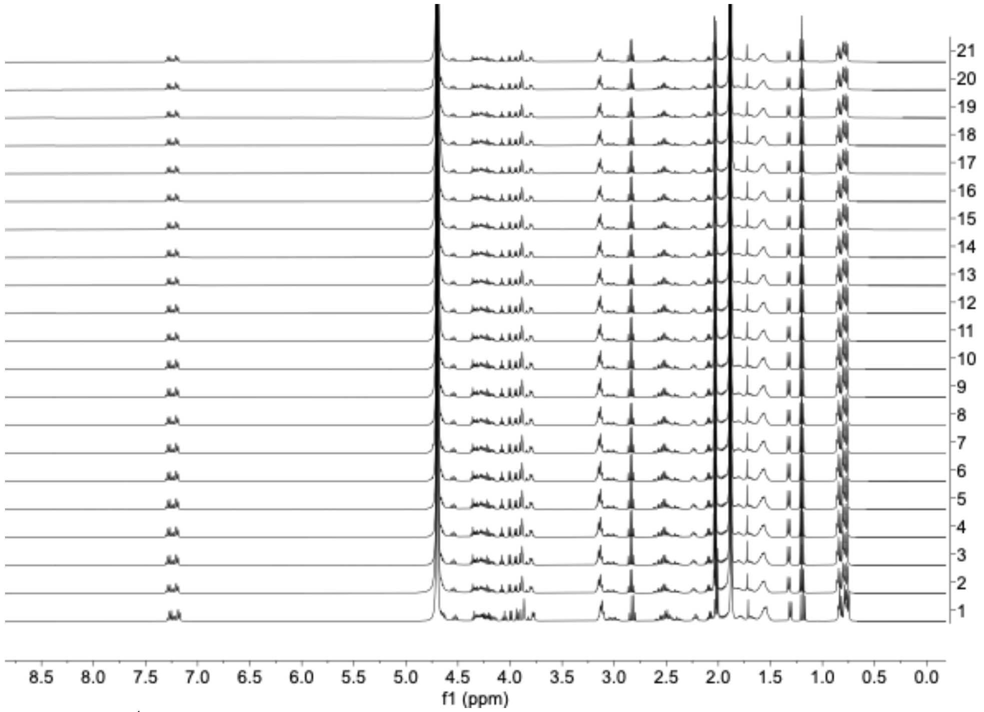

Figure S47: ^{1}H NMR spectra over time of arginine mutant (α-SynR) with 40 equiv. NaI at pD 5.2.

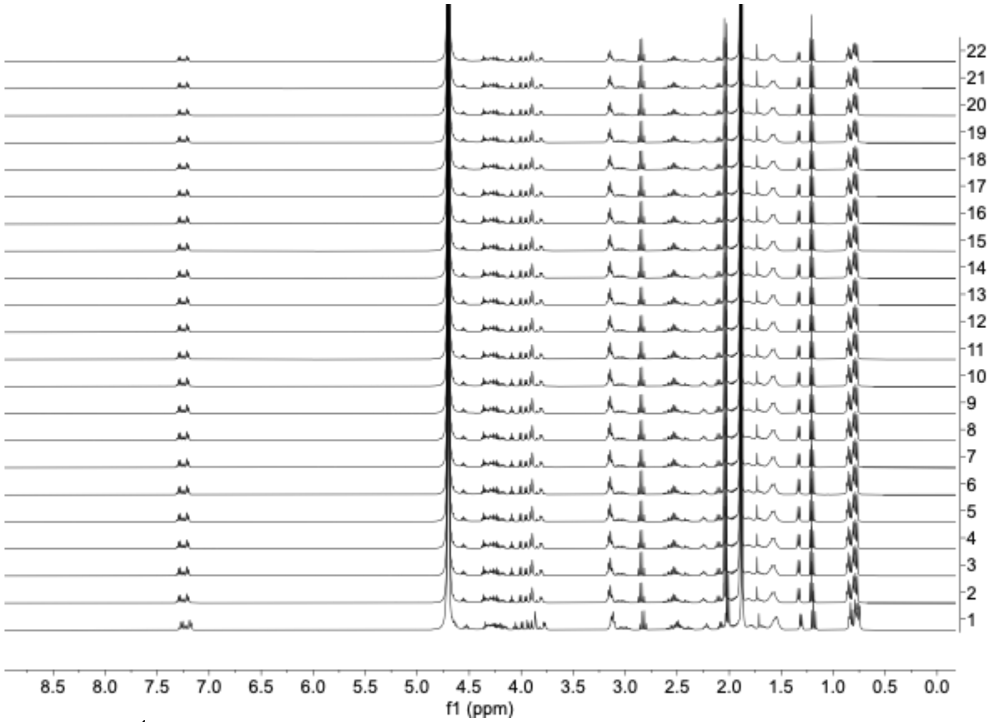

Figure S58: ^{1}H NMR spectra over time of arginine mutant (α-SynR) with 60 equiv. NaI at pD 5.2.

Table S25: Arginine triple mutant peptide (α-SynR) aggregation over time with varying concentrations of NaI.

Time (h)	Mole fraction lost (30 eq.)	error	Mole fraction lost (40 eq.)	error	Mole fraction lost (60 eq.)	error
0	0.00	-	0.00	0.008	0.00	0.000
1	0.02	-	0.02	0.002	0.01	0.007
2	0.01	-	0.02	0.006	0.02	0.007
3	0.02	-	0.04	0.009	0.02	0.007
4	0.02	-	0.04	0.013	0.03	0.007
5	0.02	-	0.05	0.013	0.04	0.000
6	0.02	-	0.06	0.011	0.06	0.007
7	0.02	-	0.08	0.012	0.11	0.030
8	0.02	-	0.09	0.019	0.14	0.011
9	0.02	-	0.12	0.018	0.18	0.028
10	0.02	-	0.14	0.027	0.22	0.031
11	0.02	-	0.17	0.029	0.26	0.039
12	0.02	-	0.19	0.025	0.30	0.046
13	0.03	-	0.21	0.034	0.34	0.015
14	0.03	-	0.22	0.037	0.37	0.015
15	0.03	-	0.24	0.031	0.38	0.009
16	0.03	-	0.25	0.028	0.39	0.016
17	0.04	-	0.27	0.034	0.40	0.011
18	0.04	-	0.27	0.035	0.40	0.009
19	0.04	-	0.28	0.033	0.40	0.001
20	0.04	-	0.29	0.030	0.40	0.007
21	0.05	-	0.29	0.028	0.40	0.006
22	0.05	-	0.30	0.025		

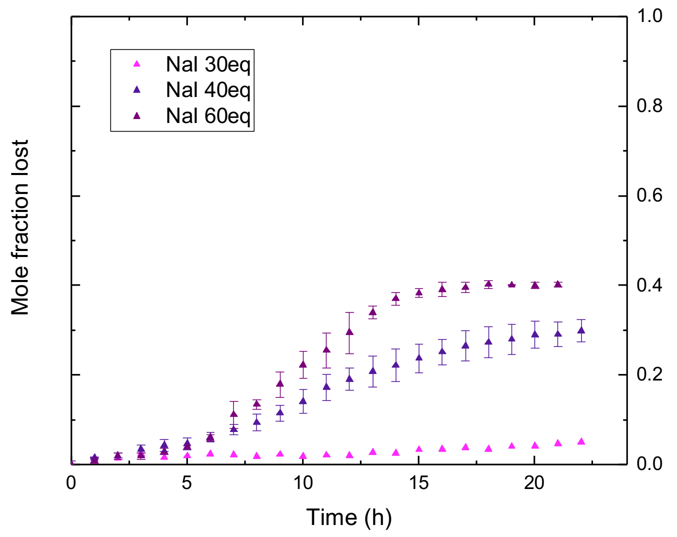

Figure S49: Mole fraction of triple arginine mutant (α-SynR) lost in the presence of varying concentrations of NaI at pD 5.2.

c) Aggregation induced by $NaReO_4$

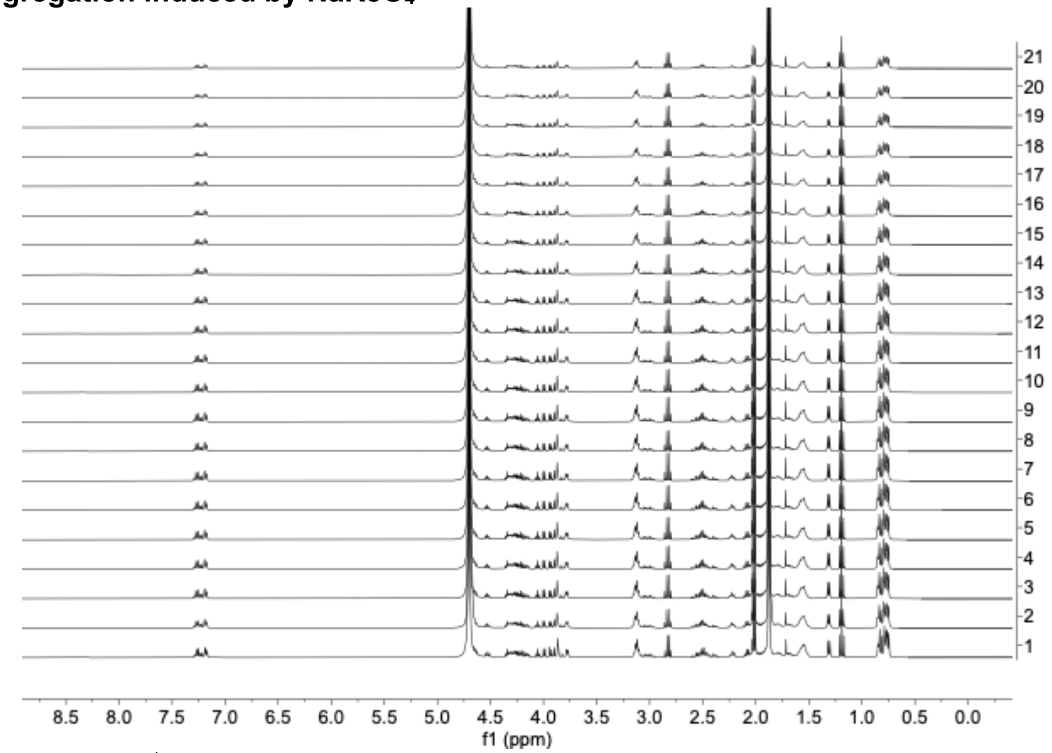

Figure S50: ^{1}H NMR spectra over time of arginine mutant (α-SynR) with 10 equiv. $NaReO_4$ at pD 5.2.

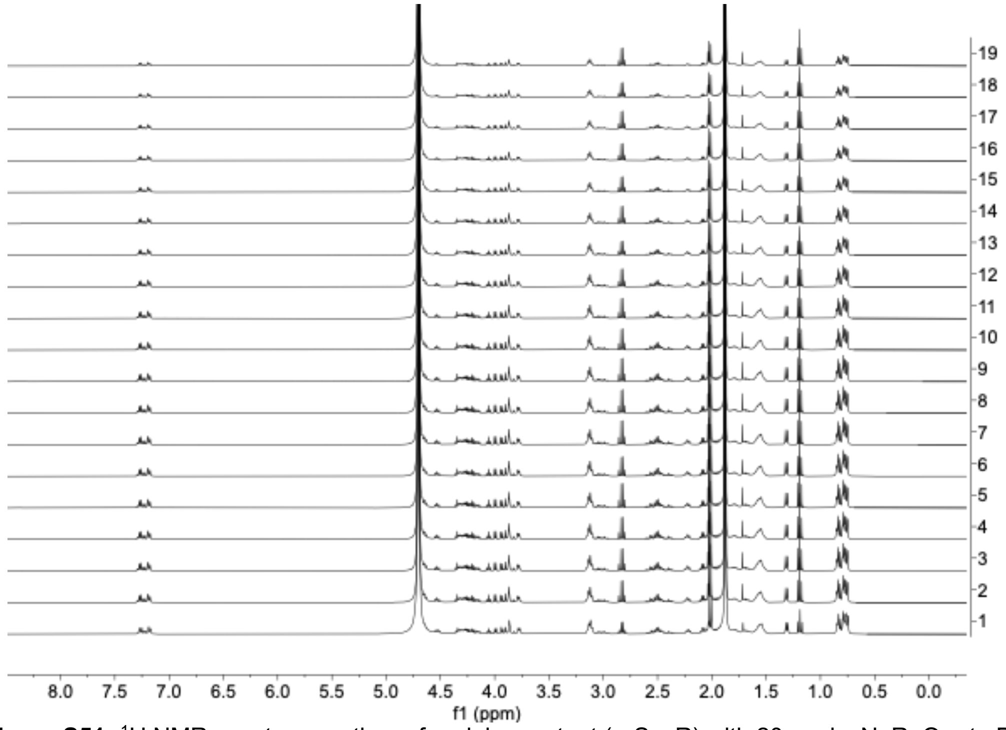

Figure S51: ^{1}H NMR spectra over time of arginine mutant (α-SynR) with 20 equiv. $NaReO_4$ at pD 5.2.

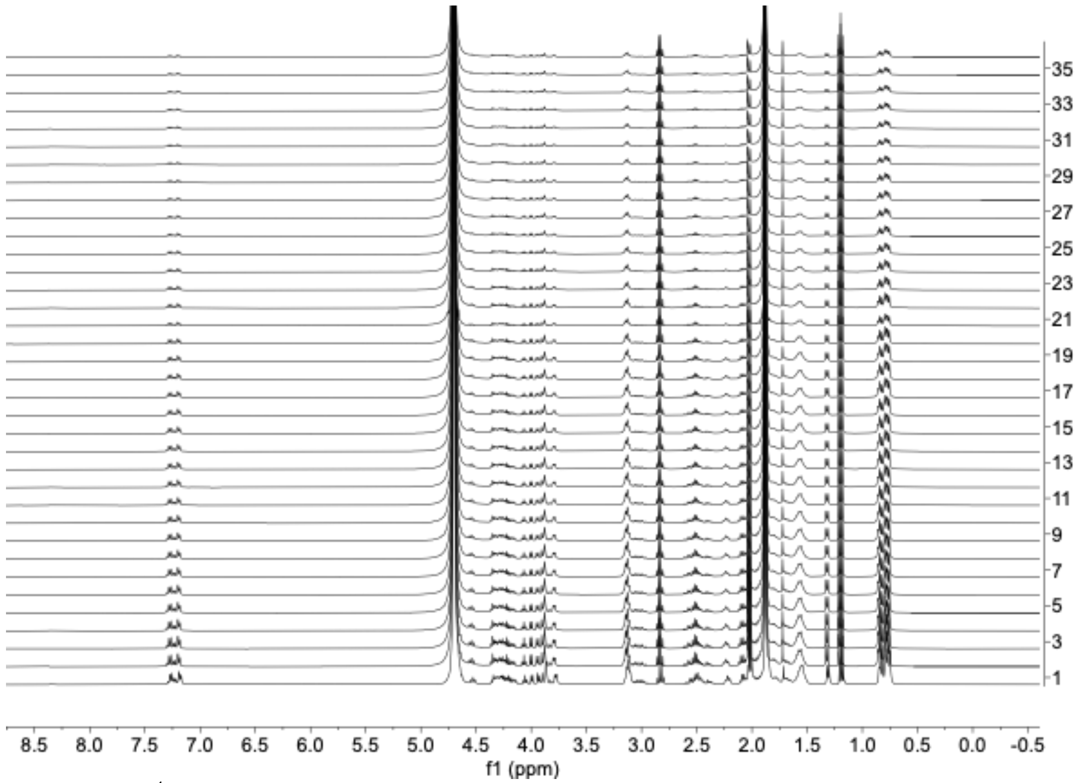

Figure S52:^{1}H NMR spectra over time of arginine mutant (α-SynR) with 30 equiv. $NaReO_4$ at pD 5.2.

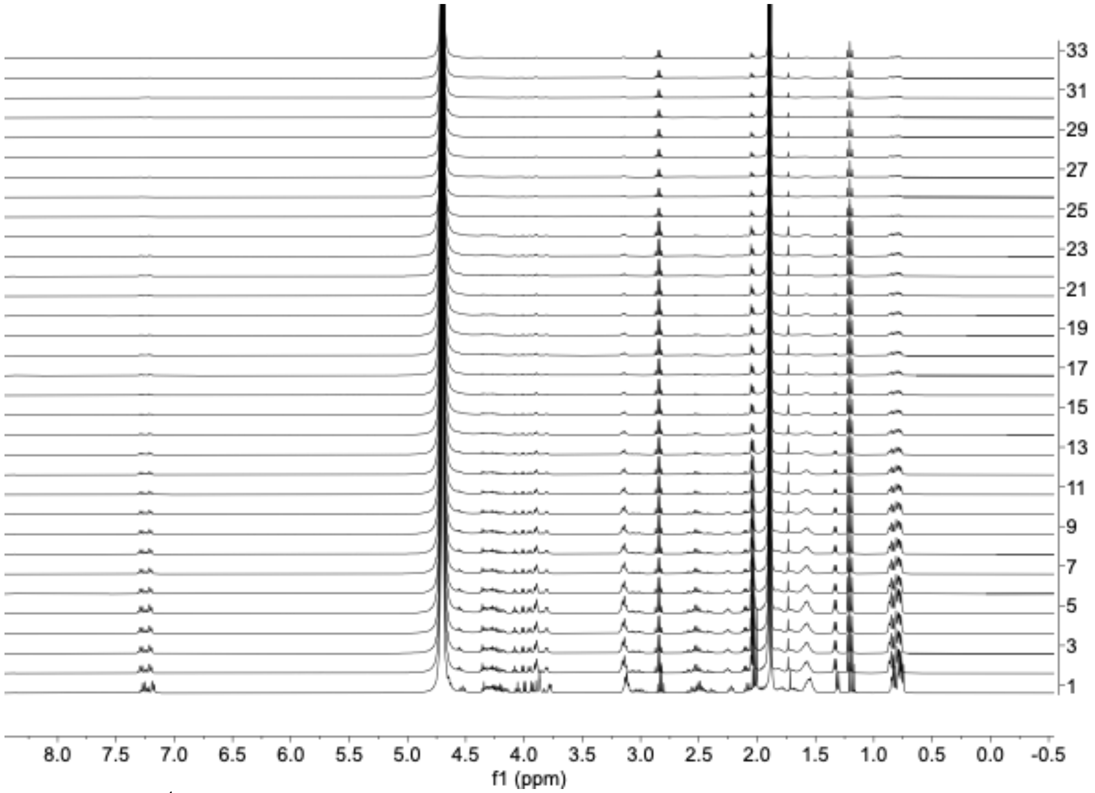

Figure S53: ^{1}H NMR spectra over time of arginine mutant (α-SynR) with 60 equiv. $NaReO_4$ at pD 5.2.

Table S26-a: Arginine triple mutant peptide (α-SynR) aggregation with varying concentrations of $NaReO_4$.

Time (h)	Mole fraction lost (10 eq.)	error	Mole fraction lost (20 eq.)	error
0	0	0.000	0.00	0.000
1	0.04	0.014	0.03	0.002
2	0.06	0.019	0.06	0.002
3	0.07	0.026	0.10	0.013
4	0.08	0.028	0.14	0.000
5	0.08	0.025	0.18	0.001
6	0.08	0.020	0.22	0.001
7	0.09	0.017	0.27	0.036
8	0.11	0.022	0.32	0.039
9	0.12	0.009	0.36	0.004
10	0.15	0.010	0.40	0.005
11	0.18	0.026	0.45	0.005
12	0.2	0.027	0.49	0.005
14	0.26	0.030	0.52	0.006
16	0.35	0.005	0.55	0.006
18	0.45	0.004	0.59	0.006
20	0.51	0.001	0.62	0.006
22	0.56	0.010	0.64	0.006

Table S26-b: Arginine triple mutant peptide (α-SynR) aggregation with varying concentrations of $NaReO_4$.

Time (h)	Mole fraction lost (30 eq.)	error	Time(h)	Mole fraction lost (60 eq.)	error
0.00	0.00	0.000	0.00	0	0
0.10	0.04	0.003	0.10	0.12	0.01
0.15	0.06	0.017	0.17	0.16	0.005
0.20	0.10	0.022	0.25	0.20	0.005
0.27	0.14	0.002	0.33	0.25	0.005
0.32	0.18	0.012	0.42	0.30	0.005
0.35	0.22	0.003	0.50	0.35	0.008
0.40	0.25	0.006	0.58	0.44	0.009
0.45	0.29	0.006	0.67	0.49	0.005
0.58	0.31	0.008	0.75	0.54	0.01
0.63	0.34	0.009	0.83	0.61	0.005
0.70	0.36	0.005	0.92	0.69	0.005
0.75	0.38	0.000	1.00	0.69	0.005
0.80	0.40	0.007	1.17	0.74	0.005
0.85	0.41	0.001	1.33	0.76	0.008
0.90	0.43	0.004	1.50	0.78	0.005
0.95	0.44	0.008	1.67	0.79	0.005
1.00	0.45	0.005	1.83	0.79	0.005
1.50	0.50	0.014	2	0.79	0.015
2	0.56	0.007	3	0.80	0.005
3	0.59	0.026	4	0.80	0.005
4	0.63	0.015	5	0.80	0.005
5	0.67	0.004	6	0.81	0.005
6	0.69	0.014	7	0.81	0.005
7	0.71	0.018	8	0.81	0.005
8	0.73	0.017	9	0.812	0.005
9	0.74	0.017	10	0.81	0.005
10	0.76	0.018	11	0.81	0.005
11	0.77	0.014	12	0.81	0.005
12	0.78	0.014	13	0.81	0.005
13	0.78	0.007	14	0.81	0.005
14	0.79	0.011	15	0.81	0.005
15	0.80	0.010	20	0.81	0.005
16	0.80	0.004			
17	0.81	0.002			
18	0.81	0.007			
20	0.82	0.007			
22	0.82	0.000			

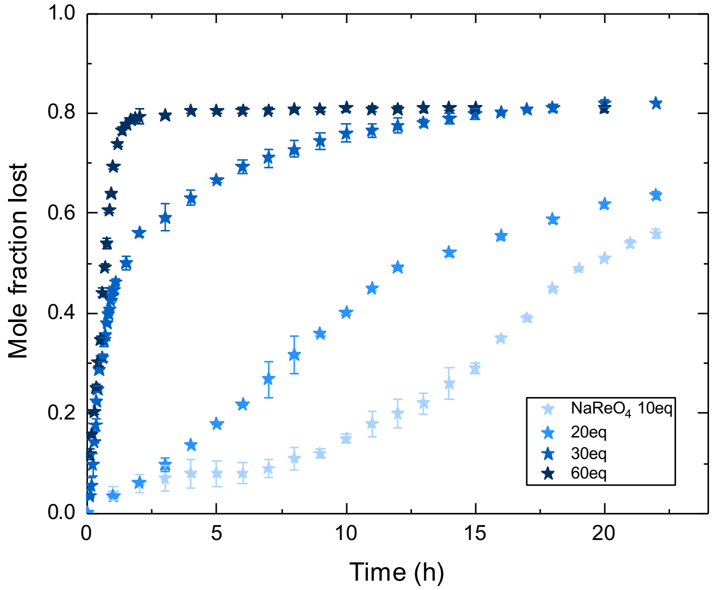

Figure S54: Mole fraction of triple arginine mutant (α-SynR) lost in the presence of varying concentrations of $NaReO_4$ at pD 5.2.

d) Aggregation induced by $NaPF_6$.

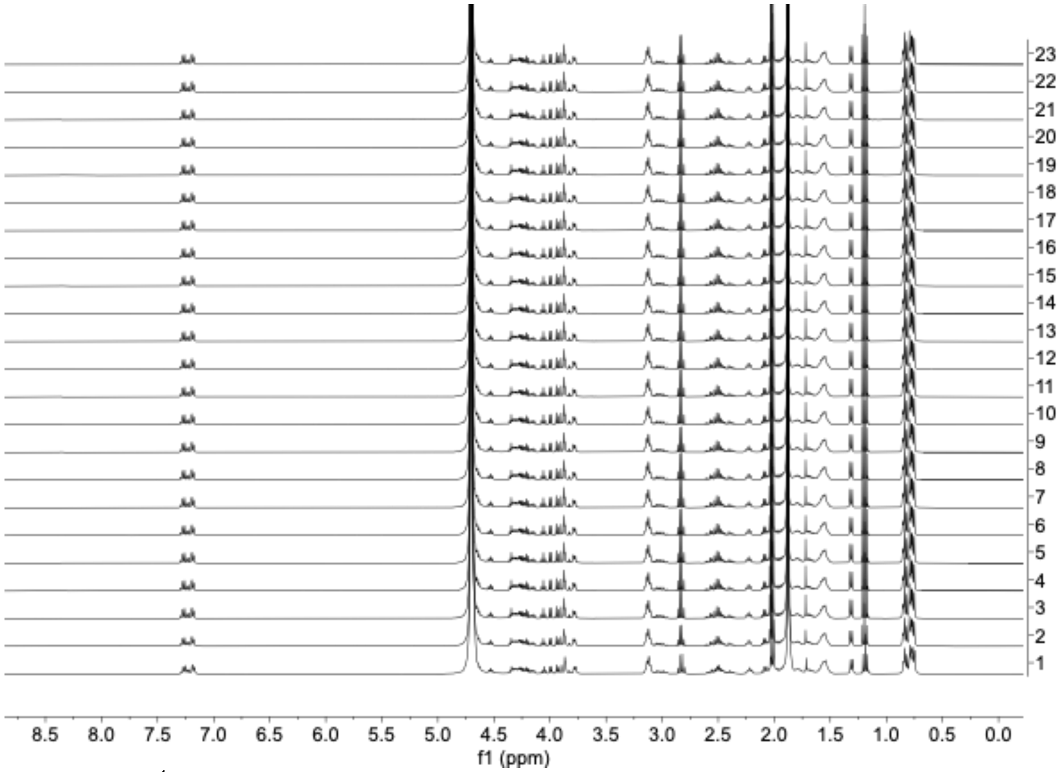

Figure S55: ^{1}H NMR spectra over time of arginine mutant (α-SynR) with 20 equiv. $NaPF_6$ at pD 5.2.

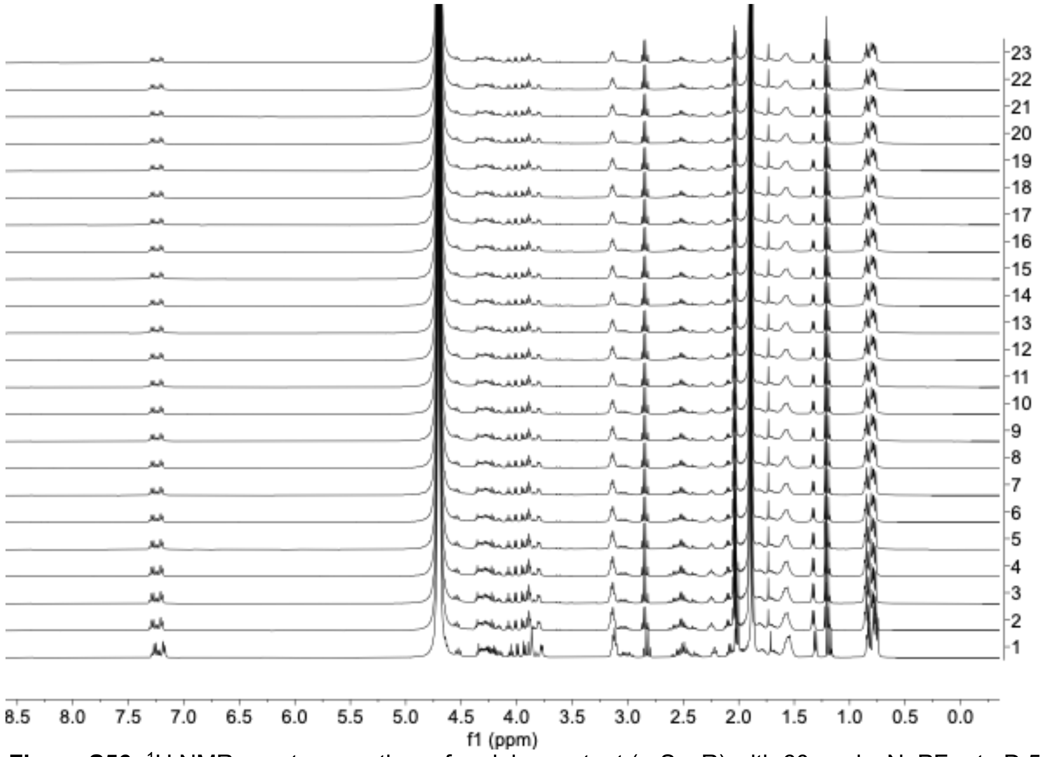

Figure S56: ^{1}H NMR spectra over time of arginine mutant (α-SynR) with 60 equiv. $NaPF_6$ at pD 5.2.

Table S27: Arginine triple mutant peptide (α-SynR) aggregation with varying concentrations of $NaPF_6$.

Time (h)	Mole fraction lost (20 eq.)	error	Mole fraction lost (60 eq.)	error
0	0	0	0	0
1	0.04	0	0.10	0.004
2	0.04	0	0.14	0.006
3	0.04	0	0.19	0.001
4	0.04	0	0.24	0.003
5	0.04	0	0.30	0.025
6	0.04	0	0.32	0.018
7	0.04	0	0.33	0.023
8	0.04	0	0.35	0.020
9	0.04	0	0.36	0.023
10	0.04	0	0.37	0.026
11	0.05	0	0.37	0.024
12	0.05	0	0.38	0.024
13	0.05	0	0.39	0.024
14	0.05	0	0.40	0.016
15	0.05	0	0.40	0.015
16	0.05	0	0.41	0.015
17	0.05	0	0.41	0.014
18	0.05	0	0.42	0.018
19	0.05	0	0.43	0.016
20	0.05	0	0.43	0.021
21	0.05	0	0.44	0.016
22	0.05	0	0.45	0.021

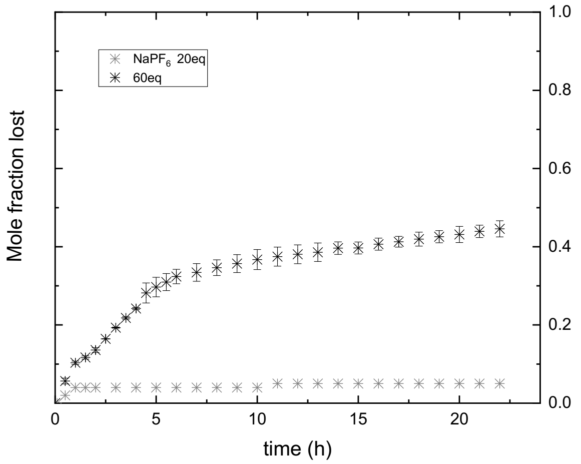

Figure S57: Mole fraction of triple arginine mutant (α-SynR) lost in the presence of varying concentrations of $NaPF_6$ at pD 5.2.

4.2 Aggregation curve fitting

In protein aggregation studies, particularly those involving amyloid fibril formation, the most common model used is a sigmoidal, nucleation-polymerization one (Figure S58). This characteristic curve consists of three distinct phases: a lag phase, an exponential growth phase, and a plateau phase. The general form of the sigmoid function is given by:[3-5]

$$f(x) = \frac{P}{1 + \exp\left(-k(t - t_{max})\right)} + b$$

where b represents the initially measured population percentage, P is the corresponding maximum value at the plateau region, k defines the maximum rate and t_{max} is the midpoint corresponding to time to maximal growth (at the midpoint between the lower and upper asymptotes).[6]

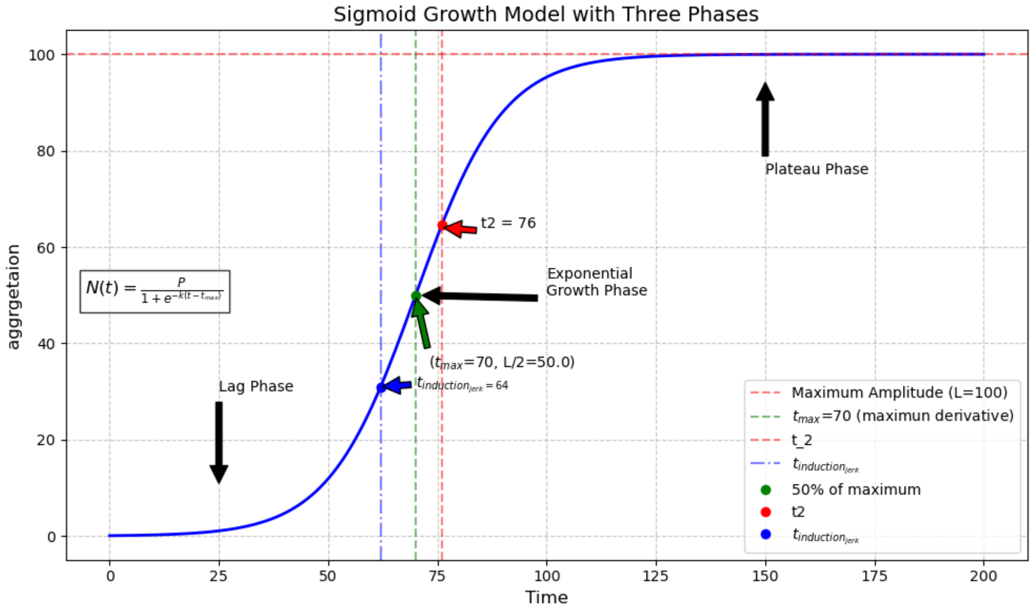

Figure S58: Sigmoid growth model with three phases

In the lag phase, primary nucleation initiates fibrillation by forming aggregates directly from monomers. Upon fibril formation, secondary processes accelerate aggregation by generating new fibril. Ultimately, equilibrium is reached, the fibril concentrations reaches a steady state, and the plateau phase is reached.

We attempted to fit our observed aggregation data to the sigmoidal growth model. The data for the aggregation of α-SynR induced by different concentrations of $NaClO_4$ at pD = 5.2. The resulting fits are shown in Figure S59. Table S28 and 29 shows the corresponding obtained parameters. Fits and the corresponding obtained parameters for the other anions follow.

a) Aggregation fitting of α-SynR with $NaClO_4$

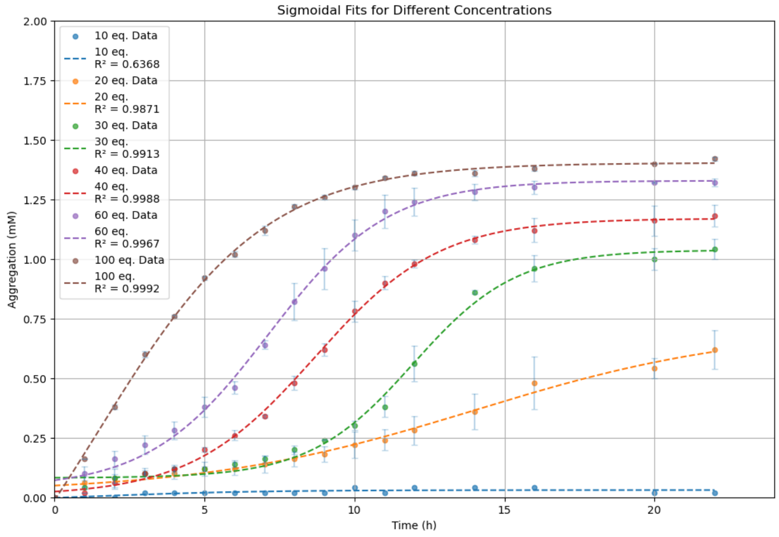

Figure S59: Sigmoid fits for the aggregation of α-SynR induced by different concentrations of $NaClO_4$ at pH = 5.2.

Table S28: Obtained fitting parameters for the sigmoid fitting of the aggregation data for α-SynR in the presence of different concentrations of $NaClO_4$ (pD = 5.2).

ClO_4^- conc. (mM)	Maximum (*P*, mM)[a]	*k* (h^{-1})	Midpoint (t_{max}, h)	Base Level (*b*, mM)	R^2
**20**[b]	–	–	–	–	–
**40**	0.69	0.22	14.03	0.02	0.9871
**60**	1.00	0.51	11.86	0.05	0.9893
**80**	1.16	0.48	8.68	0.01	0.9988
**120**	1.29	0.50	7.13	0.04	0.9967
**200**	1.42	0.55	3.69	-0.05	0.9898

[a] Effective concentration loss of both NMR silent soluble aggregates and precipitate.
[b] No fitting attempted as only marginal amounts of aggregation were detected.

Table S29: k_1, k_2 calculation and time parameters from data fitting

ClO_4^- conc. (mM)	k_1 (h^{-1})	k_2 (mM^{-1} h^{-1})	t_1 (jerk, h)	t_1 ((piecewise,h)	t_{max} (h)	k_{max} (mM^{-1} h^{-1})	t_2 (hrs)
**20**[a]	–	–	–		–	–	–
**40**	0.02	0.02	-11.03	4.84	14.03	0.04	38.91
**60**	0.01	0.11	6.38	5.61	11.86	0.13	17.34
**80**	0.03	0.11	3.47	3.68	8.68	0.14	13.90
**120**	0.03	0.13	2.57	2.96	7.13	0.16	11.68
**200**	0.07	0.11	-0.89	1.32	3.69	0.19	8.27

[a] No fitting attempted as only marginal amounts of aggregation were detected.

The calculation of k_1, k_2, $k_{induction(piecewise)}$ and $k_{induction(jerk)}$ is based on the formula:[6]

$$t_{max} = \ln(\frac{k_2[A_0]}{k_1})/(k_1 + k_2[A_0])$$

$$\left(\frac{dy}{dt}\right) \max = \frac{(k_1 + k_2[A_0])^2}{4k_2}$$

$$t_1 = t_{induction(jerk)} = \ln(2-\sqrt{3})\frac{k_2[A_0]}{k_1} \Big/ (k_1 + k_2[A_0])$$

$$t_2 = \ln(2+\sqrt{3})\frac{k_2[A_0]}{k_1} \Big/ (k_1 + k_2[A_0])$$

$$[A_0] = 2\,mM$$

b) Aggregation fitting of α-SynR with NaI

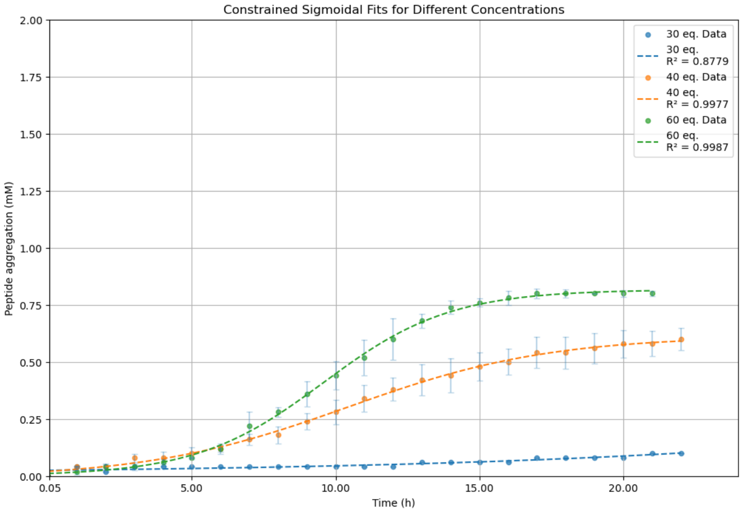

Figure S60: Sigmoid fits for different concentration of NaI aggregations

Table S30: Obtained fitting parameters for the sigmoid fitting of the aggregation data for α-SynR in the presence of different concentrations of NaI (pD = 5.2).

**I⁻ conc.** (mM)	**Maximum (*P*, mM)a**	***k* (h^{-1})**	**Midpoint (t_{max}, h)**	**Base Level (*b*, mM)**	**R^2**
**60**[b]	–	–	–	–	–
**80**	0.62	0.29	10.42	-0.01	0.9977
**120**	0.82	0.45	9.57	0.00	0.9987

[a] Effective concentration loss of both NMR silent soluble aggregates and precipitate.
[b] No fitting attempted as only marginal amounts of aggregation were detected.

Table S31: k_1, k_2 calculation and time parameters from fitting of the aggregation for α-SynR with different concentrations of NaI (pD = 5.2).

I^- conc. (mM)	k_1 (h^{-1})	k_2 ($mM^{-1}\ h^{-1}$)	t_1 (jerk, h)	t_1 (piecewise,	t_{max} (hrs)	k_{max} ($mM^{-1}\ h^{-1}$)	t_2 (hrs)
**20**[a]	–	–	–		–	–	–
**80**	0.02	0.02	-12.24	3.56	10.42	0.04	33.08
**120**	0.03	0.06	0.74	3.59	9.57	0.09	18.39

[a] No fitting attempted as only marginal amounts of aggregation were detected

c) Aggregation fitting of α-SynR with $NaReO_4$

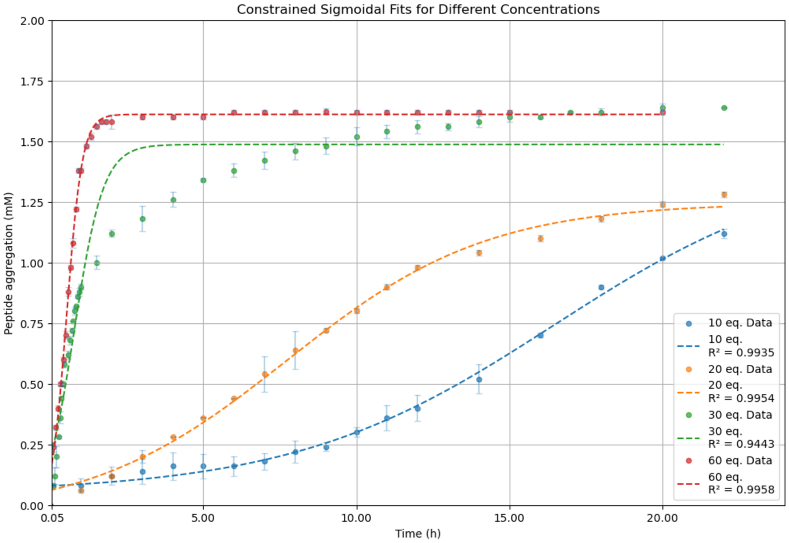

Figure S61: Sigmoid fits for different concentration of $NaReO_4$ aggregations

Table S32: Obtained fitting parameters for the sigmoid fitting of the aggregation data for α-SynR in the presence of different concentrations of $NaReO_4$ (pD = 5.2).

ReO_4^- conc. (mM)	Maximum (P, mM)[a]	k (h^{-1})	Midpoint (t_{max}, h)	Base Level (b, mM)	R^2
**20**	1.38	0.23	16.46	0.05	0.9935
**40**	1.30	0.31	7.75	-0.05	0.9954
**60**	1.54	2.11	0.78	-0.05	0.9443
**120**	1.66	3.92	0.53	-0.05	0.9958

[a] Effective concentration loss of both NMR silent soluble aggregates and precipitate.

Table 33: k_1, k_2 calculation and time parameters from fitting of the aggregation for α-SynR with different concentrations of $NaReO_4$ (pD = 5.2).

ReO_4^- conc. (mM)	k_1 (h^{-1})	k_2 ($mM^{-1}\ h^{-1}$)	t_1 (jerk, h)	t_1 (piecewise, h)	t_{max} (h)	k_{max} ($mM^{-1}\ h^{-1}$)	t_2 (hrs)
**20**	0.01	0.07	7.68	7.31	16.45	0.081	25.23
**40**	0.04	0.06	-0.94	2.81	7.74	0.10	16.44
**60**	0.33	0.41	-0.36	0.27	0.78	0.81	1.92
**80**	0.52	1.03	0.02	0.19	0.53	1.62	1.03

d) Aggregation fitting of α-SynR with $NaPF_6$

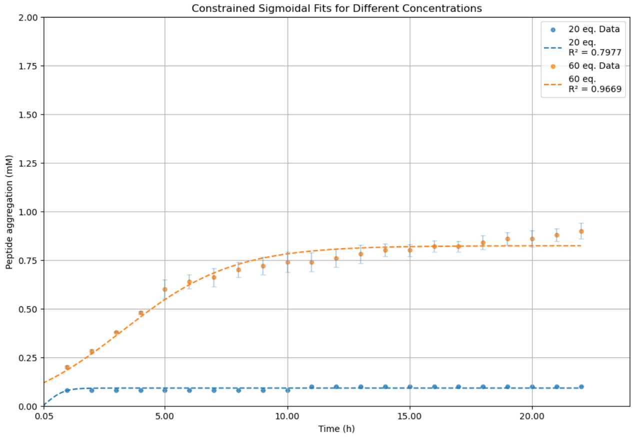

Figure S62: Sigmoid fits for different concentration of $NaPF_6$ aggregations

Table S34: Obtained fitting parameters for the sigmoid fitting of the aggregation data for α-SynR in the presence of different concentrations of $NaPF_6$ (pD = 5.2).

**PF_6^- conc.** (mM)	**Maximum (*P*, mM)**[a]	***k* (h^{-1})**	**Midpoint (t_{max}, h)**	**Base Level (*b*, mM)**	**R^2**
**20**[b]	–	–	–	–	–
**120**	0.87	0.44	3.27	-0.05	0.9669

[a] Effective concentration loss of both NMR silent soluble aggregates and precipitate.
[b] No fitting attempted as only marginal amounts of aggregation were detected.

Table S35: k_1, k_2 calculation and time parameters from fitting of the aggregation for α-SynR with different concentrations of $NaPF_6$ (pD = 5.2).

**PF_6^- conc.** (mM)	**k_1** (h^{-1})	**k_2** (mM^{-1} h^{-1})	**t_1** (jerk, h)	**t_1** (piecewise, h)	**t_{max}** (h)	**k_{max}** (mM^{-1} h^{-1})	**t_2** (h)
**20**[a]	–	–	–		–	–	–
**120**	0.05	0.03	-8.21	1.10	3.27	0.10	38.91

[a] No fitting attempted as only marginal amounts of aggregation were detected.

e) Aggregation fitting of α-SynR with 100 equiv. $NaClO_4$ at pH 2.3

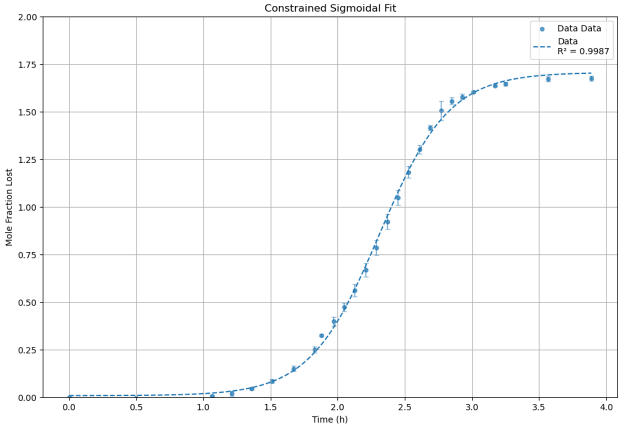

Figure S63: Sigmoid fits for 100 equiv. $NaClO_4$ aggregations at pH 2.3

Table S36: Obtained fitting parameters for the sigmoid fitting of the aggregation data for α-SynR in the presence of 100 equiv. $NaClO_4$ (pD = 2.3).

ClO_4^- conc. (mM)	Maximum (P, mM)[a]	k (h^{-1})	Midpoint (t_{max}, h)	Base Level (b, mM)	R^2
**200**	1.70	3.86	2.31	0.01	0.9987

[a] Effective concentration loss of both NMR silent soluble aggregates and precipitate.

Table S37: k_1, k_2 calculation and time parameters from fitting of the aggregation for α-SynR with 100 equiv. $NaClO_4$ (pD = 2.3).

ClO_4^- conc. (mM)	k_1 (h^{-1})	k_2 (mM^{-1} h^{-1})	t_1 (jerk, h)	t_1 (piecewise, h)	t_{max} (h)	k_{max} (mM^{-1} h^{-1})	t_2 (h)
**200**	0.002	1.64	1.91	1.71	2.31	1.64	2.71

4.3 Elemental analysis of arginine mutant aggregation particles

Elemental analysis was conducted using X-ray Energy Dispersive Spectroscopy (X-EDS) with an Oxford Instruments system and AZtec software. Initial spectroscopic examination was performed on particles obtained from aggregation experiment.

The sample under investigation was the triple arginine mutant peptide (α-SynR), aggregated with 60 equiv. sodium perrhenate ($NaReO_4$). The analysis was conducted both by examining specific points and specific area within the sample. Data was reference to that from the background.

a) Point elemental analysis

A selected point location is shown in Figure S64, and the corresponding elemental analysis results displayed in Figure S65. A summary of the elemental analysis data is provided in the following table.

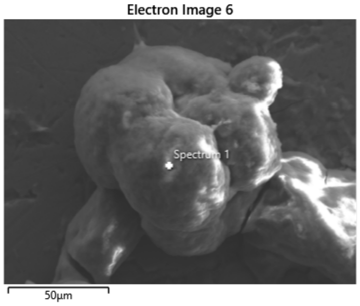

Figure S64: Microscopy image of arginine α-Syn_{15}/$NaReO_4$ precipitate, showing the point of analysis.

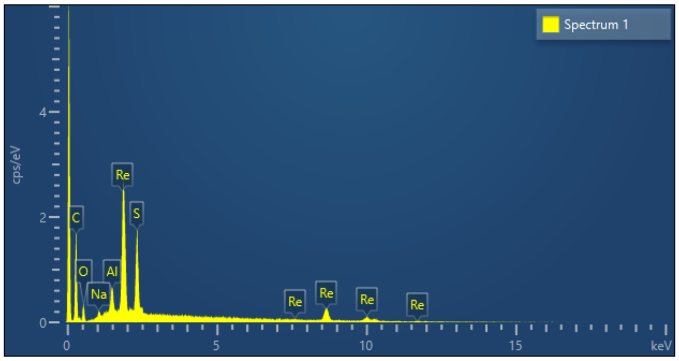

Figure S65: Elemental analysis of arginine (α-SynR)/$NaReO_4$ precipitate from point analysis.

Table S38: Elemental analysis data of arginine (α-SynR)/$NaReO_4$ precipitate using point analysis.

Element	Line Type	Apparent Conc[a]	Wt%	Wt% Sigma	Atomic %
C	K series	15.13	67.85	0.76	83.60
O	K series	5.08	13.20	0.62	12.21
Na	K series	0.91	0.53	0.07	0.34
Al	K series	1.97	1.01	0.07	0.55
S	K series	8.49	4.99	0.14	2.30
Re	L series	18.95	12.43	0.49	0.99
Total:			100.00		100.00

[a] Ratio of the measured concentration of an element within a sample based on the X-ray peak intensity in the EDS spectrum.

b) Area elemental analysis

Within the same sample (triple arginine mutant (α-SynR)/$NaReO_4$), an area was selected for elemental analysis (Figure S66). The colored maps in Figure S67 show the element distribution in the selected area for respectively carbon (red), oxygen (green), aluminum (orange), sulfur (azure), nitrogen (green), sodium (light green) and rhenium (pink). Figure S68 shows the sum spectrum. Table S39 shows the average elemental analysis obtained.

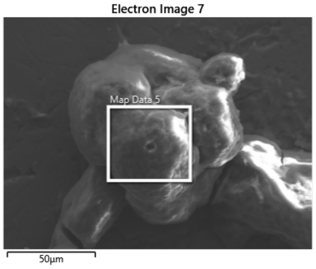

Figure S66: Area of particle probed for elemental analysis.

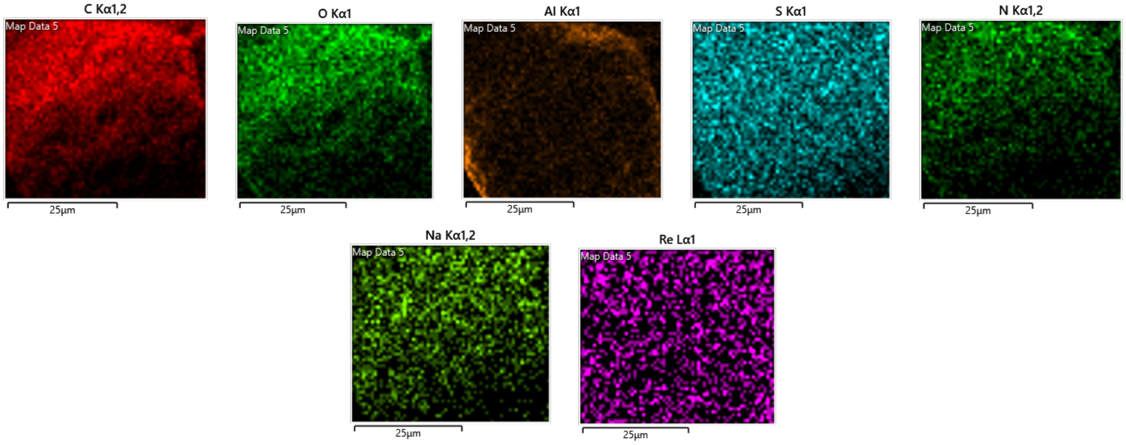

Figure S67: Elemental map data at selected area of the precipitate.

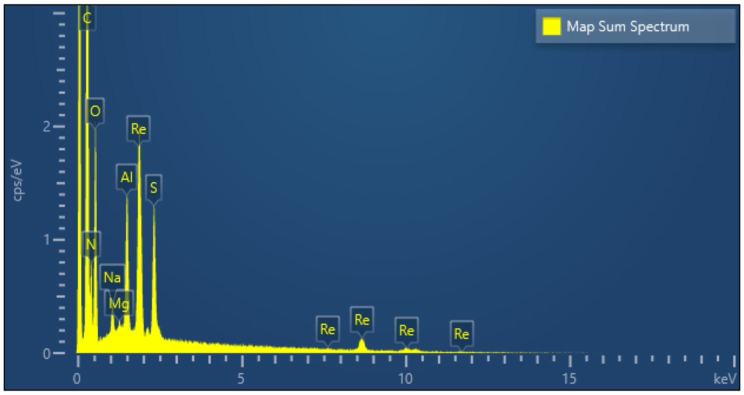

Figure S68: Elemental spectrum of selected area of particle of triple arginine mutant precipitated with $NaReO_4$.

Table S39: Elemental analysis table with $NaReO_4$ of selected area.

Element	Line Type	Apparent Conc[a]	Wt%	Wt% Sigma	Atomic %
C	K series	58.67	47.50	0.64	53.97
N	K series	39.55	25.36	0.91	24.71
O	K series	26.52	23.66	0.42	20.19
Na	K series	1.74	0.36	0.03	0.21
Mg	K series	0.28	0.06	0.01	0.03
Al	K series	5.02	0.82	0.02	0.41
S	K series	5.57	0.88	0.02	0.37
Re	L series	6.93	1.37	0.08	0.10
Total:			100.00		100.00

[a] Ratio of the measured concentration of an element within a sample based on the X-ray peak intensity in the EDS spectrum.

c) Background area elemental analysis

To obtain a reference, a background area was selected (S69). The colored maps in Figure S70 show the element distribution in the selected area for respectively: aluminum (orange), magnesium (orange), oxygen (green), and carbon (red). Figure S71 shows the sum spectrum. Table S40 shows the average elemental analysis.

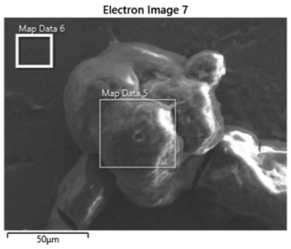

Figure S69: Area of particle probed for elemental analysis.

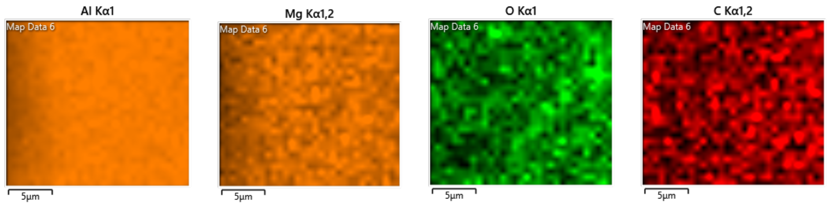

Figure S70: elemental map data at referenced background area.

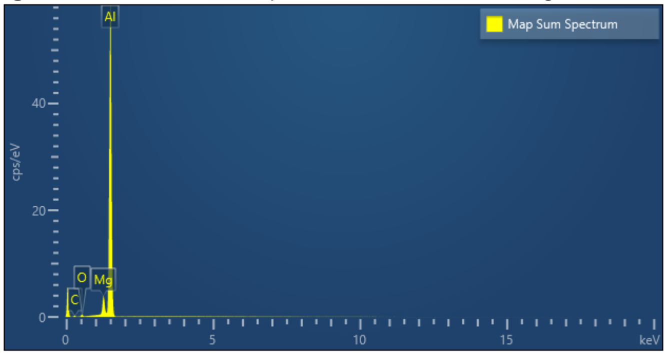

Figure S71: elemental spectrum of selected background area.

Table S40: Elemental analysis table with selected background area.

Element	Line Type	Apparent Conc[a]	Wt %	Wt % Sigma	Atomic %
C	K series	0.71	21.54	0.78	36.80
O	K series	1.82	6.08	0.21	7.80
Mg	K series	5.32	4.11	0.06	3.47
Al	K series	85.83	68.26	0.69	51.92
Total:			100.00		100.00

[a] Ratio of the measured concentration of an element within a sample based on the X-ray peak intensity in the EDS spectrum.

5. Circular Dichroism studies

CD spectra were obtained using a Jasco J-810 spectropolarimeter. The αSyn peptide derivatives were dissolved in a solution containing 10 mM phosphate buffer and the pH of the solution adjusted to 2.3. The sample volume was 200 µL containing 100 µM of each peptide. Wavelength scans ranging from 240 to 190 nm were conducted using a quartz cuvette with a path length of 0.1 cm and a slit width of 1 mm. Baseline was also collected, and all spectra were all baseline-subtracted.

Anion titrations were conducted on three derivatives using phosphate buffer at pH 2.3 and sodium acetate buffer at pH 5.2. Titration of αSynH at pH 5.2 was omitted due to its poor solubility under this condition. To avoid interference from the strong absorption of chloride ions in the far-UV region, titrations were performed only in the presence and absence of $NaClO_4$. Salt concentrations were tested at 0 mM, 60 mM, 120 mM, and 200 mM. All data is shown in Figures S72-S77).

5.1 Three peptide derivatives at pH 2.3

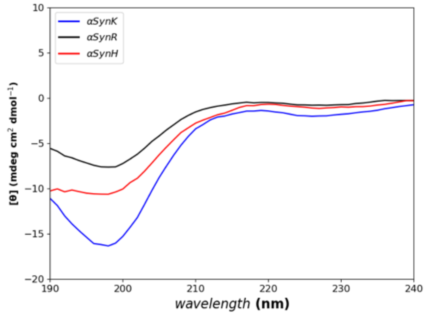

Figure S72: CD data of three peptides at phosphate pH 2.3

5.2 CD spectra in response to added anions

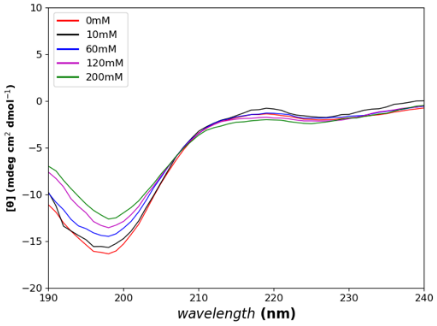

Figure S73: Anion titration of αSynK at phosphate pH 2.3

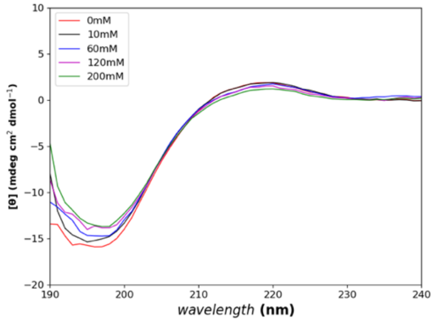

Figure S74: Anion titration of αSynK at sodium acetate pH 5.2

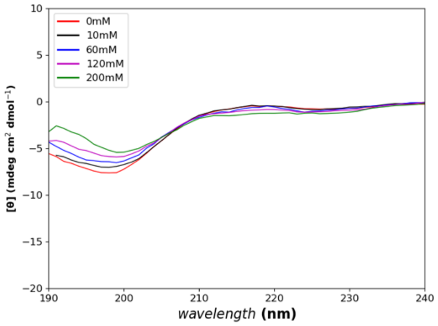

Figure S75: Anion titration of αSynR at phosphate pH 2.3

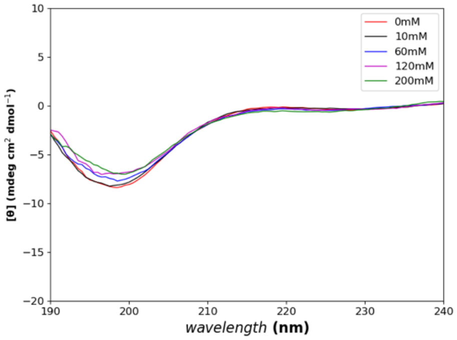

Figure S76: Anion titration of αSynR at sodium acetate pH 5.2

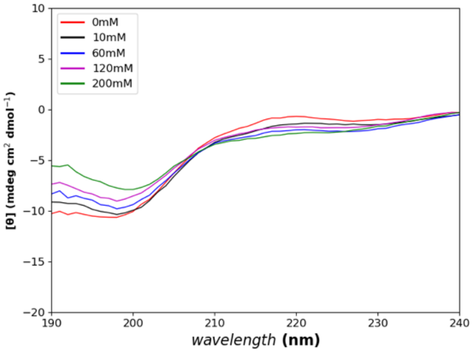

Figure S77: Anion titration of αSynH at phosphate pH 2.3**.**

6. Computational studies

The starting α-Syn_{15} structure was obtained by truncating the crystal structure of wild-type α-Syn bound to a membrane (1XQ8).[7] This truncated peptide was either used as a starting point for simulation and replica exchange, or pulled into the fully extended conformation (umbrella sampling and pulling along the z-axis) for anion binding studies in that conformation. In the case of the triple arginine or triple histidine mutants, the necessary residue changes were made before pulling into the extended conformation.

All simulations in this work were carried out using GROMACS 2016.3. For the replica exchange simulations, the peptides were modelled using the Amber03ws[8] all-atom force field, water was modeled using the TIP4P2005 potential, the ions modelled using the generalized Amber force field (GAFF)[9] and their partial charges obtained from AM1-BCC calculations. Each peptide was placed in a solvated periodic cubic box by itself or with 260 mM of either NaCl or $NaClO_4$. The peptide was in the +1 state (with one counter chloride or perchlorate anion added). The total simulation time was 200 ns and generated 100,000 timeframes. The equations of motion were integrated using a leapfrog algorithm with a time step of 2 fs. Thirty-four replicas were considered at temperatures ranging from 277.15 to 376.15K. These temperatures were assigned using the Patriksson and van der Spoel algorithm[10] to ensure maximal exchanges between neighboring temperatures. An exchange rate of 21% was obtained. All simulations were run in the isothermal-isobaric ensemble (25°C and 1 bar), with the temperature and pressure maintained using the Nosé-Hoover thermostat,[11, 12] and the Parrinello-Rahman barostat.[13]

6.1 R_g characterization

Radius analysis was calculated from the replica exchange results, with errors calculated using the bootstrap method.[14]

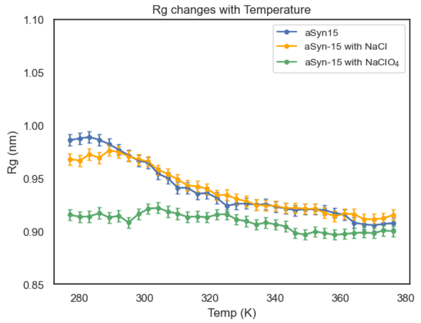

Figure S78: Radius of gyration (R_g) of wild-type α-Syn_{15} in the presence of NaCl or $NaClO_4$.

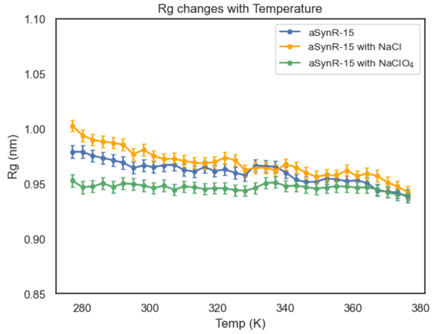

Figure S79: Radius of gyration (R_g) of the triple arginine mutant (α-SynR) in the presence of NaCl or $NaClO_4$.

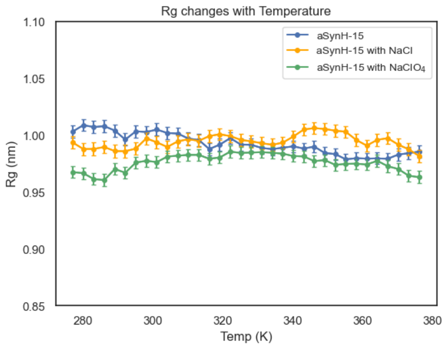

Figure S80: Radius of gyration of (R_g) triple histidine mutant (α-SynH) in the presence of NaCl or $NaClO_4$.

The calculated radius of gyration distribution of the three peptides derivatives at 298.15K are shown below in Figure S81-S83.

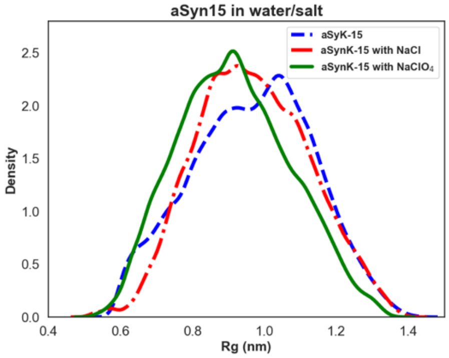

Figure 81: Radius of gyration (R_g) of wild type α-Syn_{15} in the presence of NaCl or $NaClO_4$ at 298.15K.

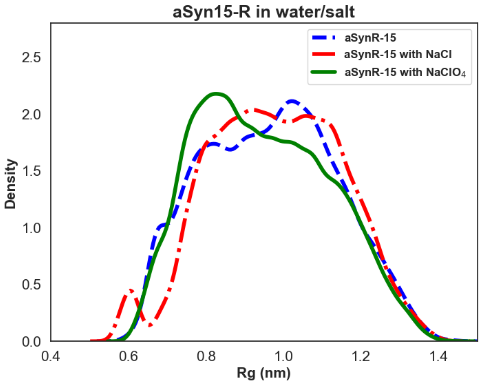

Figure S82: Radius of gyration (R_g) of the triple arginine mutant of (α-SynR) in the presence of NaCl or $NaClO_4$ at 298.15K.

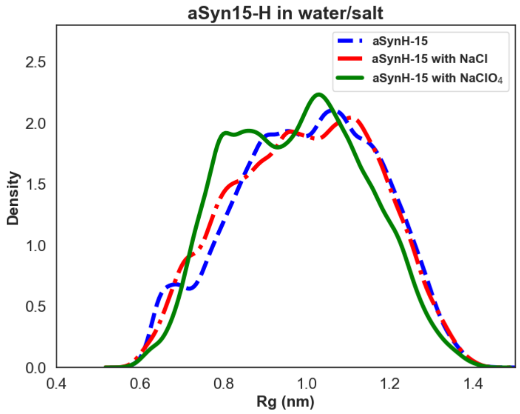

Figure S83: Radius of gyration (R_g) of the triple histidine mutant of (α-SynH) in the presence of NaCl or $NaClO_4$ at 298.15K.

6.2 RMSD characterization

The root mean square deviation (RMSD) of the peptide backbone was calculated to monitor conformational stability throughout the molecular dynamic simulation. The reference structure and corresponds to the initial structure before simulation.

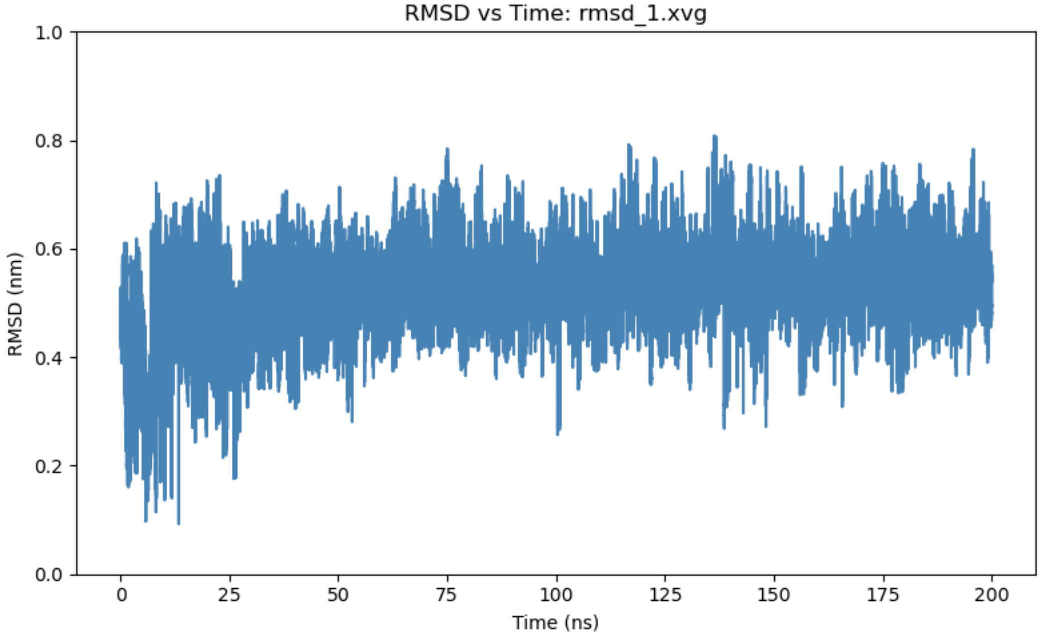

Figure S84: RMSD of wild-type α-Syn_{15} backbone over time.

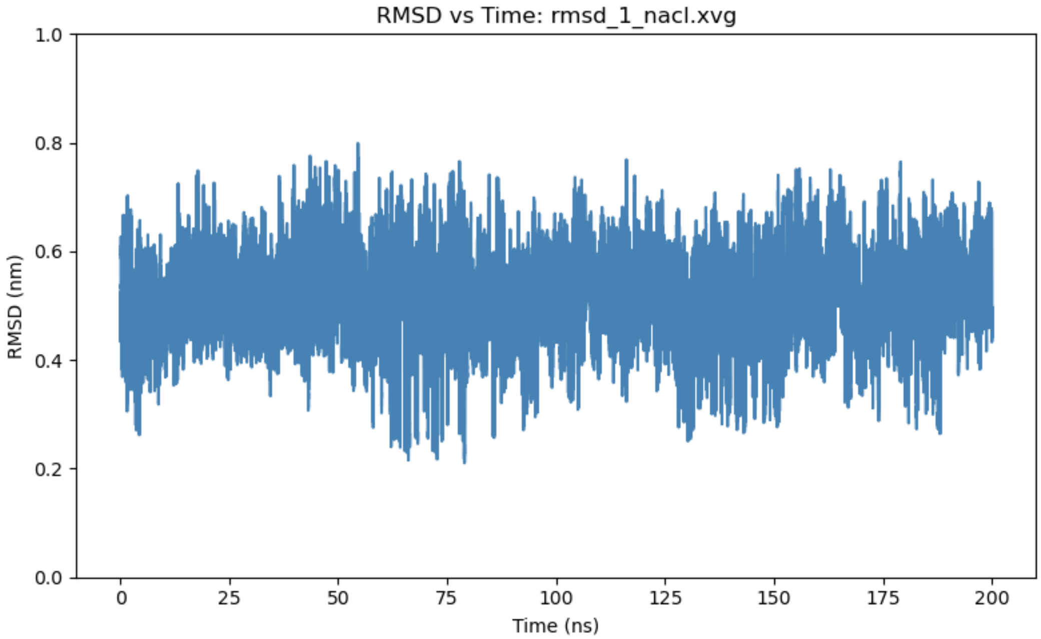

Figure S85: RMSD of wild-type α-Syn$_{15}$ backbone over time in the presence of NaCl.

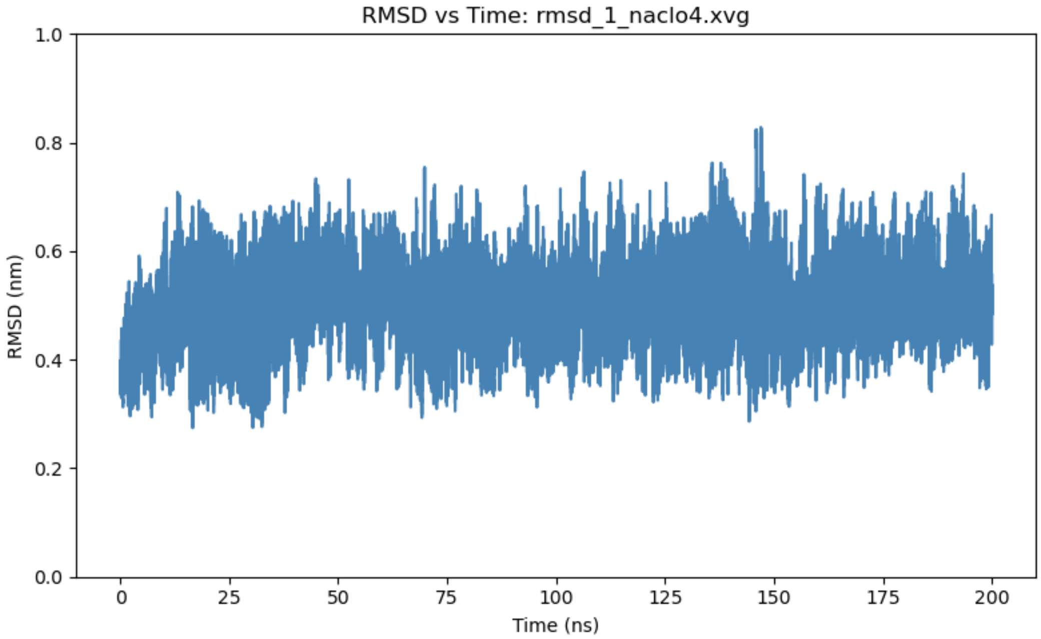

Figure S86: RMSD of wild-type α-Syn$_{15}$ backbone over time in the presence of $NaClO_4$.

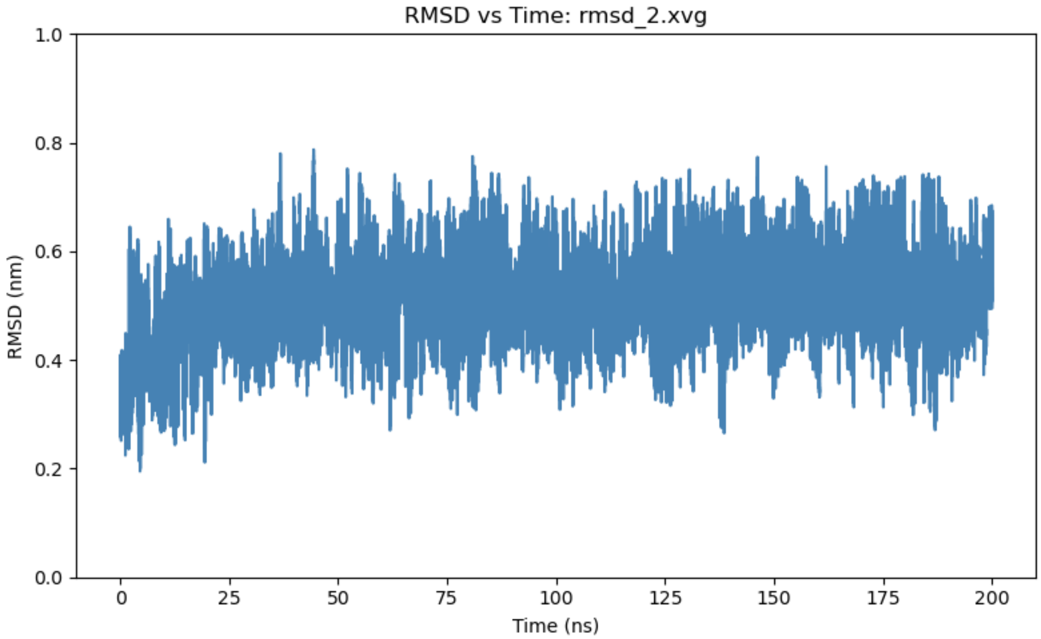

Figure S87: RMSD of triple arginine mutant (α-SynR) backbone over time.

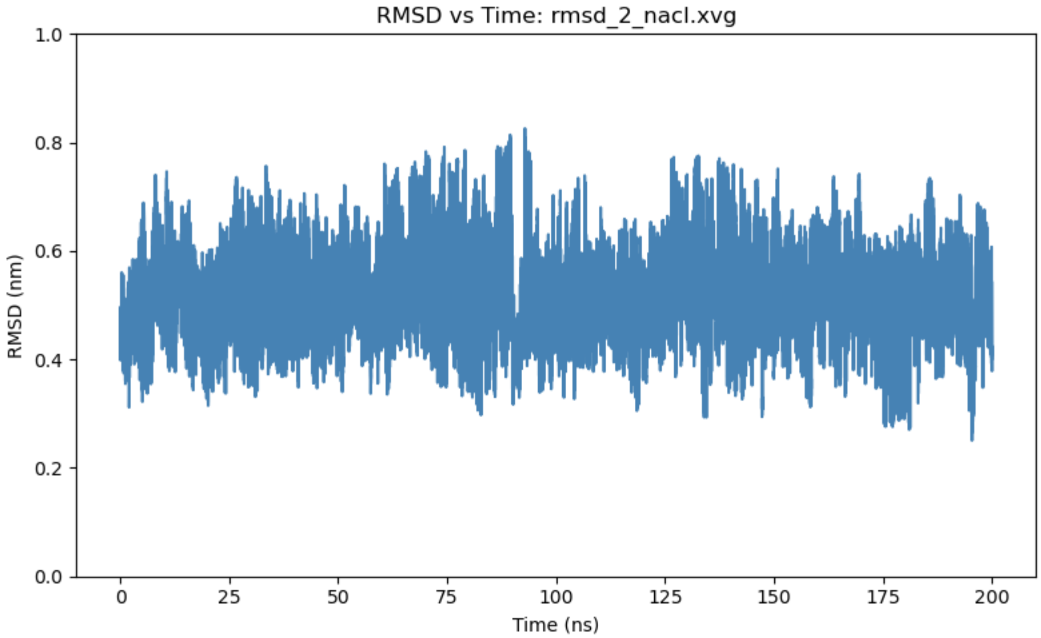

Figure S88: RMSD of triple arginine mutant (α-SynR) backbone over time in the presence of NaCl.

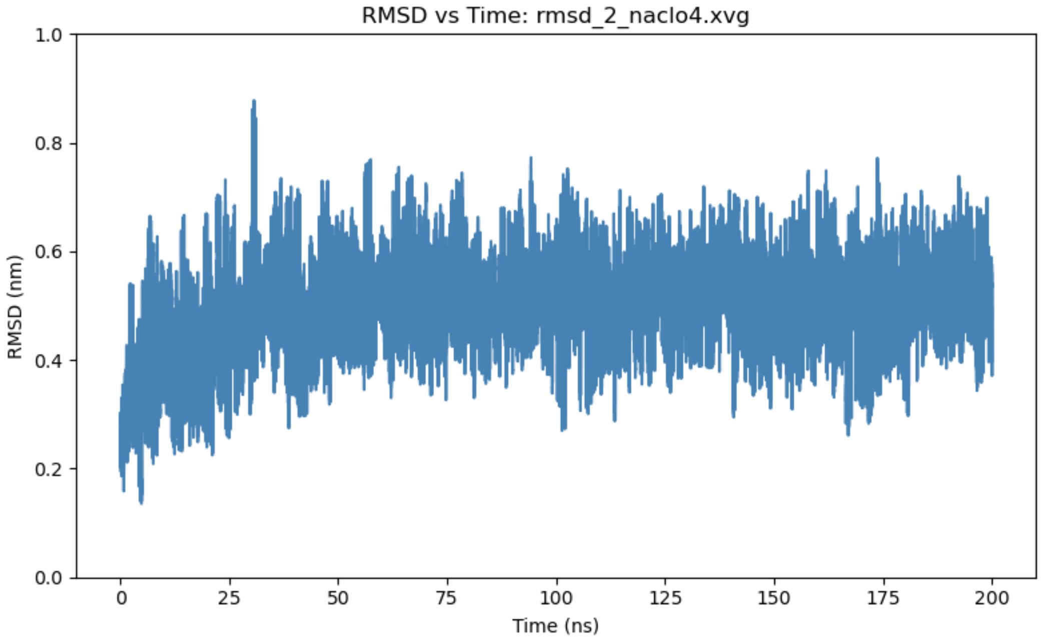

Figure S89: RMSD of triple arginine mutant (α-SynR) backbone over time in the presence of $NaClO_4$.

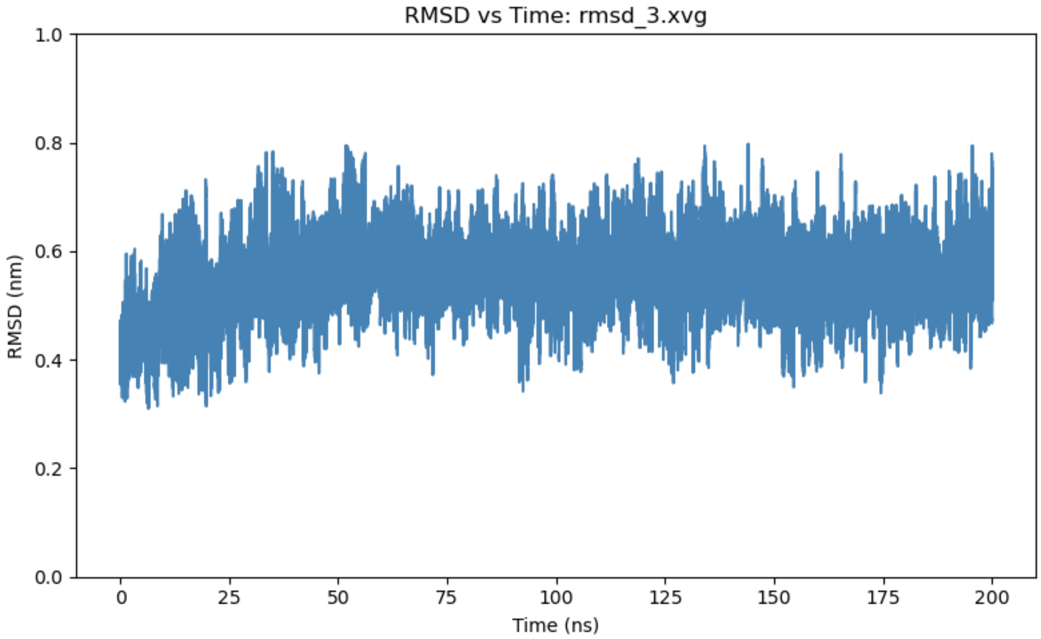

Figure S90: RMSD of the triple histidine mutant of (α-SynH) backbone over time.

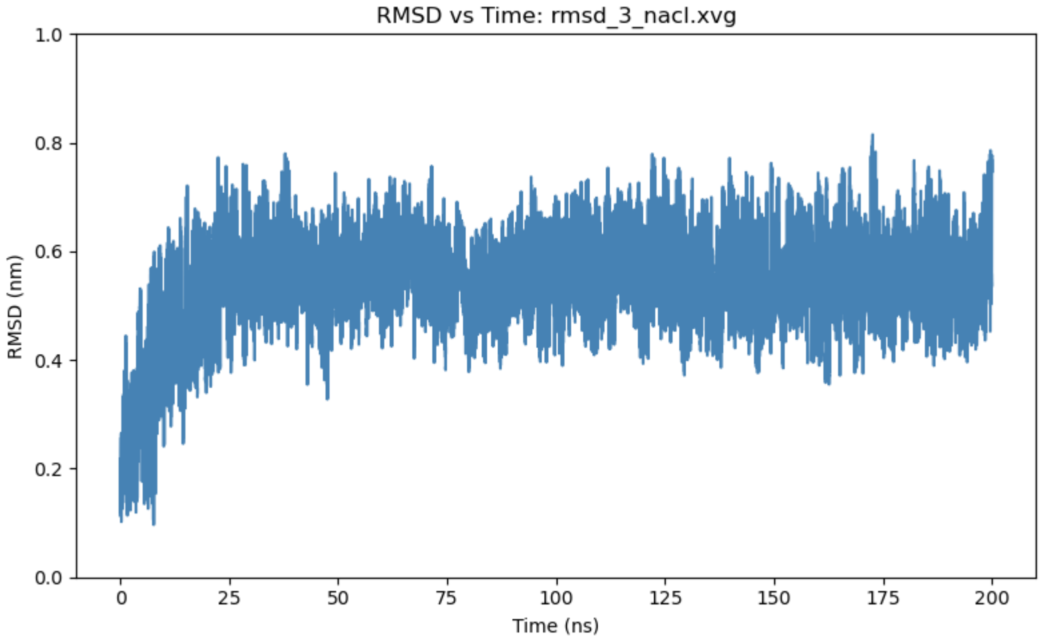

Figure S91: RMSD of the triple histidine mutant of (α-SynH) backbone over time in the presence of NaCl.

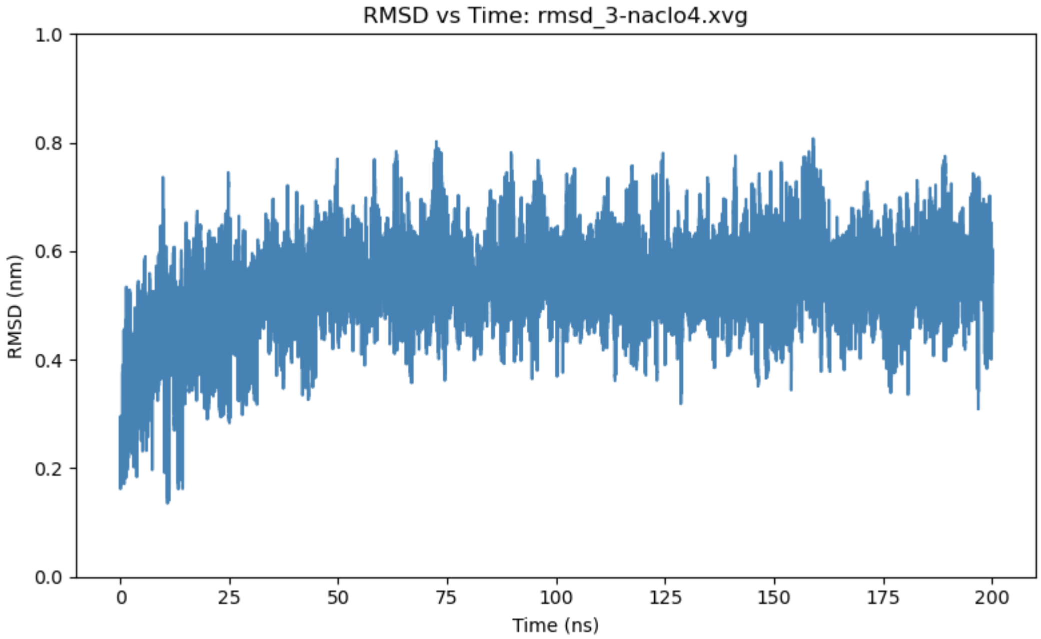

Figure S92: RMSD of the triple histidine mutant of (α-SynH) backbone over time in the presence of $NaClO_4$.

6.3 Anion binding visualization

For the simulations involving anion accumulation, separate (50 ns) MD simulations were carried out (all other parameters listed above were otherwise the same). We used TRAVIS to calculate the spatial distribution function of anions around the peptides and visualized using VMD,[15] and rendered with POV-Ray 3.7.[16, 17] In each case shown below, the isovalue was set to eight times the bulk ion density.

Three peptide derivatives were investigated: wild-type α-Syn$_{15}$, and the triple arginine and histidine mutants. Peptides were examined in both an extended and a relaxed conformation. For the former, derivatives were stretched to their extended conformation using a pulling simulation and then held in the fixed conformation during the simulation. The relaxed conformation was selected from the replica exchange calculations (Section 6.1) using the Rg value with the highest probability.[8]

a) Anion accumulation on wild-type α-Syn$_{15}$

Wild-type α-Syn$_{15}$ with $NaClO_4$ and NaCl. The peptide is modeled in an extended (Figure S84 and S85) and compressed (Figure S86 and S87) conformation.

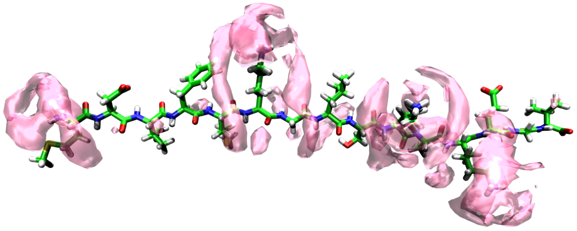

Figure S93: Visualization of ClO_4^- accumulation around the wild-type α-Syn$_{15}$ peptide in the extended conformation.

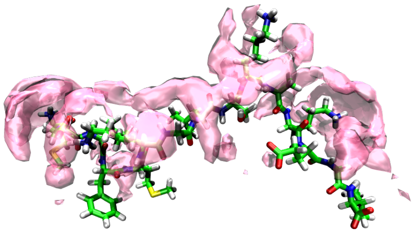

Figure S94: Visualization of ClO_4^- accumulation around the wild-type α-Syn$_{15}$ peptide in the compressed conformation.

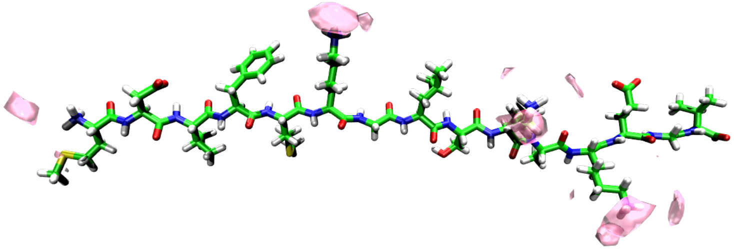

Figure S95: Visualization of Cl^- accumulation around the wild-type α-Syn$_{15}$ peptide in the extended confirmation.

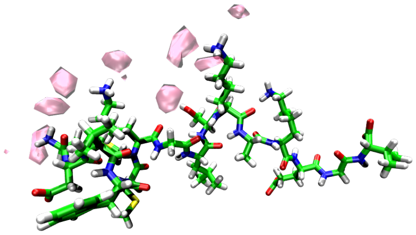

Figure S96: Visualization of Cl^- accumulation around the wild-type α-Syn$_{15}$ peptide in the compressed conformation.

b) Anion accumulation on arginine mutant α-SynR

Similarly, the triple arginine mutant α-SynR with $NaClO_4$ and NaCl was analyzed. The peptide was modeled in an extended (Figure S88 and S89) and compressed (Figure S90 and S91) conformation.

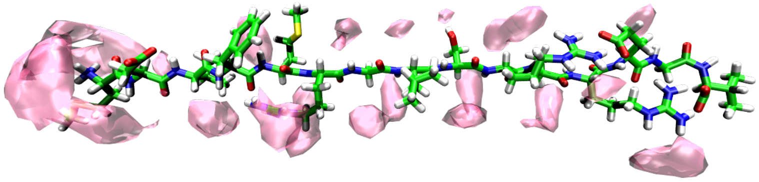

Figure S97: Visualization of ClO_4^- accumulation around the arginine mutant of α-Syn in the extended confirmation.

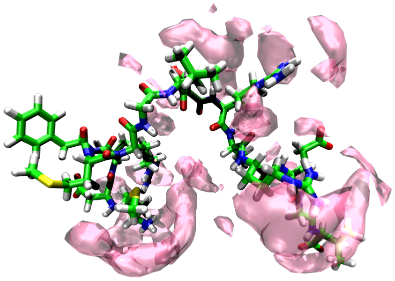

Figure S98: Visualization of ClO_4^- accumulation around the arginine mutant of α-Syn in the compressed confirmation.

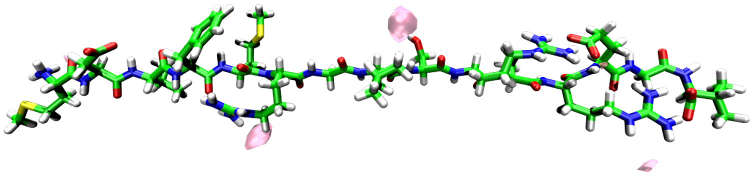

Figure S99: Visualization of Cl^- around the arginine mutant of α-Syn in the extended confirmation.

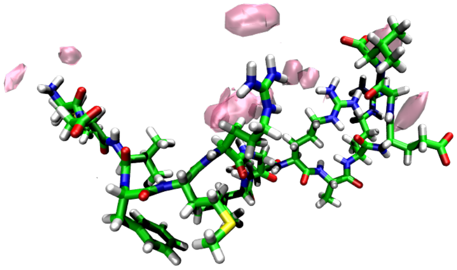

Figure S100: Visualization of Cl^- accumulation around the arginine mutant of α-Syn in the compressed confirmation.

c) Anion accumulation on arginine mutant α-SynH

Similarly, the triple histidine mutant α-SynH with $NaClO_4$ and NaCl was analyzed. The peptide is modeled in an extended (Figure S92 and S93) and compressed (Figure S94 and S96) conformation.

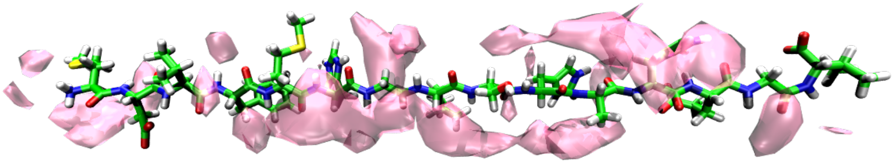

Figure S101: Visualization of ClO_4^- accumulation around the histidine mutant of α-Syn in the extended confirmation.

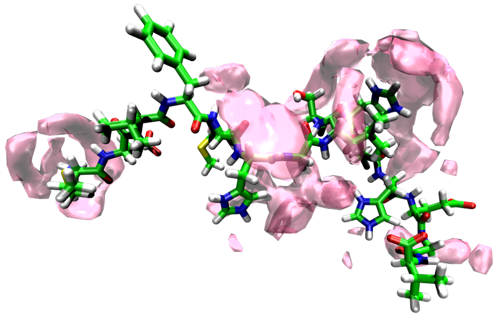

Figure S102: Visualization of ClO_4^- accumulation around the histidine mutant of α-Syn in the compressed confirmation.

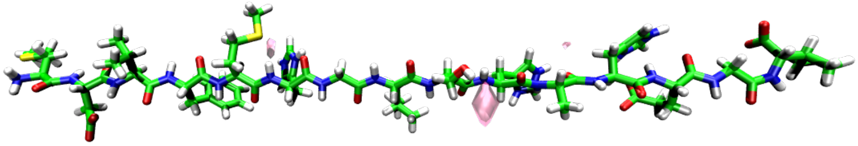

Figure S103: Visualization of Cl^- accumulation around the histidine mutant of α-Syn in the extended confirmation.

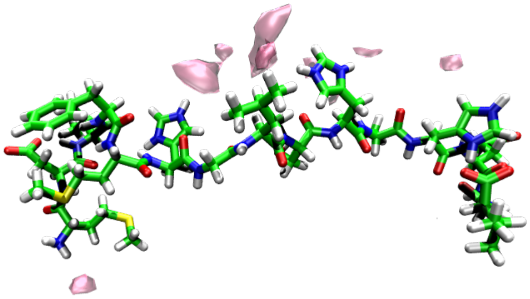

Figure S104: Visualization of Cl^- accumulation around the histidine mutant of α-Syn in the compressed confirmation.

8. References

(1) Maltsev, A. S.; Ying, J.; Bax, A. Impact of N-terminal acetylation of α-synuclein on its random coil and lipid binding properties. *Biochemistry* **2012**, *51* (25), 5004-5013. DOI: 10.1021/bi300642h.

(2) Minch, M. J. Orientational dependence of vicinal proton-proton NMR coupling constants: The Karplus relationship. *Concepts in Magnetic Resonance* **1994**, *6* (1), 41-56. DOI: https://doi.org/10.1002/cmr.1820060104.

(3) Zapadka, K. L.; Becher, F. J.; Uddin, S.; Varley, P. G.; Bishop, S.; Gomes Dos Santos, A. L.; Jackson, S. E. A pH-Induced Switch in Human Glucagon-like Peptide-1 Aggregation Kinetics. *J Am Chem Soc* **2016**, *138* (50), 16259-16265. DOI: 10.1021/jacs.6b05025.

(4) Morris, A. M.; Watzky, M. A.; Agar, J. N.; Finke, R. G. Fitting Neurological Protein Aggregation Kinetic Data via a 2-Step, Minimal/"Ockham's Razor" Model: The Finke−Watzky Mechanism of Nucleation Followed by Autocatalytic Surface Growth. *Biochemistry* **2008**, *47* (8), 2413-2427. DOI: 10.1021/bi701899y.

(5) Nielsen, L.; Khurana, R.; Coats, A.; Frokjaer, S.; Brange, J.; Vyas, S.; Uversky, V. N.; Fink, A. L. Effect of Environmental Factors on the Kinetics of Insulin Fibril Formation: Elucidation of the Molecular Mechanism. *Biochemistry* **2001**, *40* (20), 6036-6046. DOI: 10.1021/bi002555c.

(6) Bentea, L.; Watzky, M. A.; Finke, R. G. Sigmoidal Nucleation and Growth Curves Across Nature Fit by the Finke–Watzky Model of Slow Continuous Nucleation and Autocatalytic Growth: Explicit Formulas for the Lag and Growth Times Plus Other Key Insights. *J. Phys. Chem. C* **2017**, *121* (9), 5302-5312. DOI: 10.1021/acs.jpcc.6b12021.

(7) Ulmer, T. S.; Bax, A.; Cole, N. B.; Nussbaum, R. L. Structure and Dynamics of Micelle-bound Human α-Synuclein. *J. Biol. Chem.* **2005**, *280* (10), 9595-9603. DOI: https://doi.org/10.1074/jbc.M411805200.

(8) Best, R. B.; Zheng, W.; Mittal, J. Balanced Protein-Water Interactions Improve Properties of Disordered Proteins and Non-Specific Protein Association. *J Chem Theory Comput* **2014**, *10* (11), 5113-5124. DOI: 10.1021/ct500569b.

(9) Wang, J.; Wolf, R. M.; Caldwell, J. W.; Kollman, P. A.; Case, D. A. Development and testing of a general amber force field. *J. Comput. Chem.* **2004**, *25* (9), 1157-1174. DOI: 10.1002/jcc.20035.

(10) Patriksson, A.; van der Spoel, D. A temperature predictor for parallel tempering simulations. *Phys. Chem. Chem. Phys.* **2008**, *10* (15), 2073-2077, 10.1039/B716554D. DOI:10.1039/B716554D.

(11) Nosé, S. A unified formulation of the constant temperature molecular dynamics methods. *J. Chem. Phys.* **1984**, *81* (1), 511-519. DOI: 10.1063/1.447334.

(12) Hoover, W. G. Canonical dynamics: Equilibrium phase-space distributions. *Physical Review A* **1985**, *31* (3), 1695-1697. DOI: 10.1103/PhysRevA.31.1695.

(13) Parrinello, M.; Rahman, A. Polymorphic transitions in single crystals: A new molecular dynamics method. *J. Appl. Phys.* **1981**, *52* (12), 7182-7190. DOI: 10.1063/1.328693.

(14) Efron, B., Tibshirani, R.J. *An Introduction to the Bootstrap* 1994.

(15) Humphrey, W.; Dalke, A.; Schulten, K. VMD: visual molecular dynamics. *J. Mol. Graphics* **1996**, *14* (1), 33-38.

(16) Plachetka, T. POV Ray: persistence of vision parallel raytracer. In *Proc. of Spring Conf. on Computer Graphics, Budmerice, Slovakia*, 1998; Vol. 123, p 129.

(17) Persistence of Vision Pty. Ltd. (2004), Persistence of Vision Raytracer (Version 3.6) http://www.povray.org/download/.
